# Synthetic Analogs of the Alkaloid Cassiarin A with Enhanced Antimalarial Activity

**DOI:** 10.3390/ph18071018

**Published:** 2025-07-09

**Authors:** Thomas Klaßmüller, Timo Reiß, Florian Lengauer, Che Julius Ngwa, Karin Bartel, Gabriele Pradel, Franz Bracher

**Affiliations:** 1 Department of Pharmacy, Center for Drug Research, Pharmaceutical Chemistry, Ludwig-Maximilians University, 81377 Munich, Germany; 2 Division of Cellular and Applied Infection Biology, RWTH Aachen University, 52074 Aachen, Germany; 3 Department of Pharmacy, Center for Drug Research, Pharmaceutical Biology, Ludwig-Maximilians University, 81377 Munich, Germany

**Keywords:** cassiarin A, alkaloid, synthesis, antimalarial activity, structure–activity relationships, *Plasmodium falciparum*, cytotoxicity

## Abstract

**Background:** Among the alkaloids from *Cassia siamea*, cassiarin A has outstanding antiprotozoal activity, but structure–activity relationships for this chemotype were only poorly understood until now. **Methods:** We worked out efficient approaches to hitherto underexplored analogs (12 examples) on three synthesis routes which mainly comprised variations in the methyl groups at C-2 and C-5. The new compounds were tested for antiprotozoal and cytotoxic activities. **Results:** Introduction of a (substituted) benzene ring at C-2 led to a significant enhancement of activity against *Plasmodium falciparum*, while modifications of the methyl group at C-5 and the phenolic group had detrimental effects. Two of the 2-phenyl analogs further showed a resistance index comparable to the one of the reference drug chloroquine. Although the novel derivatives did not show hemolytic effects, investigation on human endothelial (HUVEC) cells at relevant concentrations indicated strong cytotoxic effects on human cells. **Conclusions:** Systematic structure modifications of cassiarin A led to a significant enhancement of antiplasmodial activity, but the observed strong cytotoxicity to human cells renders this library of cassiarin A derivatives inadequate for drug development.

## 1. Introduction

The tropical disease malaria remains one of the most devastating health problems, with over 263 million cases and 597,000 deaths worldwide in the year 2023, particularly affecting children under the age of five in Africa [1]. Malaria is caused by unicellular parasites of the genus *Plasmodium*, which are transmitted through the bite of an infected female *Anopheles* mosquito. Among the five human-pathogenic *Plasmodium* species, *P. falciparum* is the predominant variant in the WHO African region, where approximately 95% of global cases and deaths occur and is therefore responsible for most of the malaria deaths. The asexual stages of the parasites are responsible for the clinical manifestation of the disease, which can be categorized into uncomplicated malaria, characterized by symptoms such as fever, chills, and headaches, and severe malaria, which presents more serious symptoms including coma, severe anemia, organ dysfunction, and ultimately death [2]. Global efforts to eradicate malaria are repeatedly undermined by the parasites’ rapid development of resistance to commonly used drugs including artemisinin combination therapies (ACTs) [3]. To date, the two malaria vaccines RTS,S and R21 are recommended by the WHO for the use in endemic areas, preferably in regions with a high transmission rate. The vaccines are currently used in 17 countries for routine childhood immunization and additional countries have already expressed their plans to introduce the vaccines [1]. Nevertheless, the development of further vaccines and new drugs is essential in combating malaria, with compounds based on natural active substances historically playing a significant role in this endeavour.

Rational therapy of malaria is one of the outstanding topics in history of medicine, since already in the 17th century the antimalarial activity of the extracts of bark of the *Cinchona* trees was discovered in South America and this drug was brought to Europe in the same century. In 1820, Pelletier isolated quinine (**1**) as the active principle, and the elucidation of quinine’s structure in 1911 can be regarded as the starting point of a rational development of antimalarials by means of organic synthesis. One of the first hallmarks in this regard was the synthesis of chloroquine (**2**) by Andersag in 1934. Chloroquine is an antimalarial drug that works by accumulating in the acidic food vacuole of malaria parasites, where it inhibits heme detoxification, leading to parasite death. However, increasing resistance has emerged due to mutations in the *pfcrt* (Plasmodium falciparum chloroquine resistance transporter) gene. These mutations lead to an altered transporter protein of the food vacuole membrane, resulting in enhanced drug efflux, which in consequence impairs the effectiveness of chloroquine [4,5,6]. As a result, chloroquine is no longer recommended as a first-line treatment in many malaria-endemic regions, prompting the need for alternative therapies.

**Figure 1:**
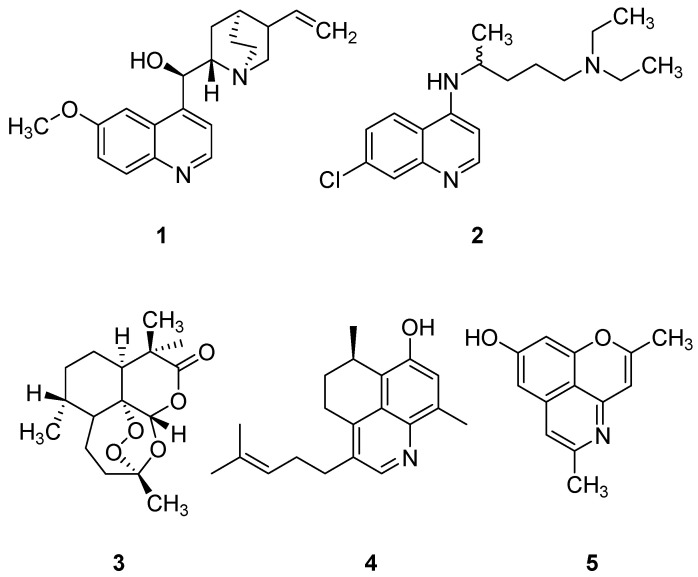
Structures of antimalarial compounds: quinine (**1**), chloroquine (**2**), artemisinin (**3**), microthecaline A (**4**), and cassiarin A (**5**).

Due to the emergence of resistances of *Plasmodium* species against chloroquine and related antimalarial drugs, the development of novel antimalarials, ideally with novel molecular modes of action, is an ongoing challenge for drug research. Here again, natural products played an outstanding role, and a major development was the introduction of artemisinin (**3**) (isolated from the Chinese plant *Artemisia annua*) and semisynthetic analogs thereof, like artesunate and artemether (Figure 1) [7]. Since, not surprisingly, resistances have also been described for artemisinin and its derivatives [8], the development of next-generation antimalarials is an urgent and permanent need. Once again, natural products as a source of lead structures with outstanding molecular diversity can inspire drug development [9]. Among them, fused quinoline derivatives like microthecaline A (**4**) [10] as well as cassiarin A (**5**) [11] (Figure 1) are very attractive due to their unique structures.

In 2007, the Morita group reported on the isolation of cassiarin A (**5**) (together with the related cassiarin B) from the leaves of *Cassia siamea* (Leguminosae), which have been widely used in traditional medicine for the treatment of malaria [11]. This alkaloid has an unprecedented tricyclic skeleton consisting of a 3-methylisoquinolin-6-ol coupled with a 2-methyl-4*H*-pyran ring and exhibits potent antiplasmodial activity. A couple of further cassiarin alkaloids (cassiarins C-E [12], cassiarin F [13], cassiarins G, H, J, and K [14]) as well as siamaalkaloids A-C [15] and an *O*-methyl derivative of **5** [16]) have been isolated from the same plant in the following, but none of them reached the antiplasmodial potency of cassiarin A (**5**). Further, cassiarin A showed vasorelaxant effects in isolated rat arteries [17]. Moderate cytotoxicity and binding to DNA and topoisomerase II has been demonstrated for several derivatives of cassiarin A (**5**) obtained by functionalization of the phenolic hydroxy group [18]. Different molecular targets have been claimed for cassiarin A (**5**) based on docking analyses, such as tryptophanyl-tRNA synthetase and 1-deoxy-D-xylose-5-phosphate reductoisomerase proteins [19], and for other cassiarins (D, E) the enzymes pteridine reductase 1 and *N-*myristoyl transferase were claimed as targets [20].

Since 2008, several total syntheses of cassiarin A (**5**) have been published, including a semisynthetic conversion of the natural product barakol [21] and diverse fully synthetic approaches [22,23,24,25]. But only few of these publications dealt with the synthesis of analogs and SAR studies concerning antimalarial activity. Variations in the cassiarin A molecule have been performed in the form of *N*-alkyl derivatives [21] and alkylation/acylation and deletion of the phenolic hydroxy group [18,24], ending up with the preliminary evidence that both the unsubstituted pyridine nitrogen and the free phenolic hydroxy group are essential for antimalarial activity. The importance of the unsaturated pyran ring was recognized early due to the poor antimalarial activity of cassiarin C, the dihydropyran analog of cassiarin A [12]. In contrast, *O*-methylation enhanced the vasorelaxant activity [24]. Only Yao and Yao [23] performed some modifications of the methyl group at the pyridine ring (three examples), but did not report on biological activities. The methyl group at the pyran ring has, to the best of our knowledge, not yet been subject to systematic modifications.

This prompted us to investigate the impact of variations in both methyl groups of cassiarin A (**5**) on antimalarial activity.

## 2. Results

### 2.1. Syntheses of Cassiarin A Analogs

**Figure 2:**
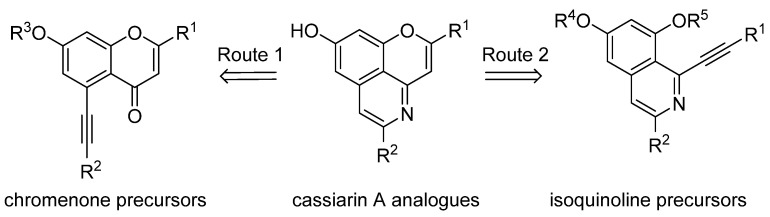
Routes for the construction of the pyrano[2,3,4-*ij*]isoquinoline core of cassiarin A analogs.

In order to obtain a diverse collection of cassiarin A analogs by total synthesis, we utilized and optimized two established approaches to the backbone of the alkaloid. In Route 1 (Figure 2) we started, inspired by the work of Yao [23] and Morita [24] from substituted chromenones, which were cyclized to the target pyrano[2,3,4-*ij*]isoquinolines via 5-alkynylchromenones under insertion of ammonia. Route 2, inspired by the work of the Honda [22] and Vaquero groups [25] in this field, included cyclization of alkynes as well, but utilized 8-oxygenated isoquinoline intermediates. In both cases, the residue replacing the respective methyl group of cassiarin A (**5**) was to be introduced by readily available terminal alkynes via a Sonogashira coupling, a reaction which has meanwhile found broad application in natural products synthesis [26].

**Scheme 1:**
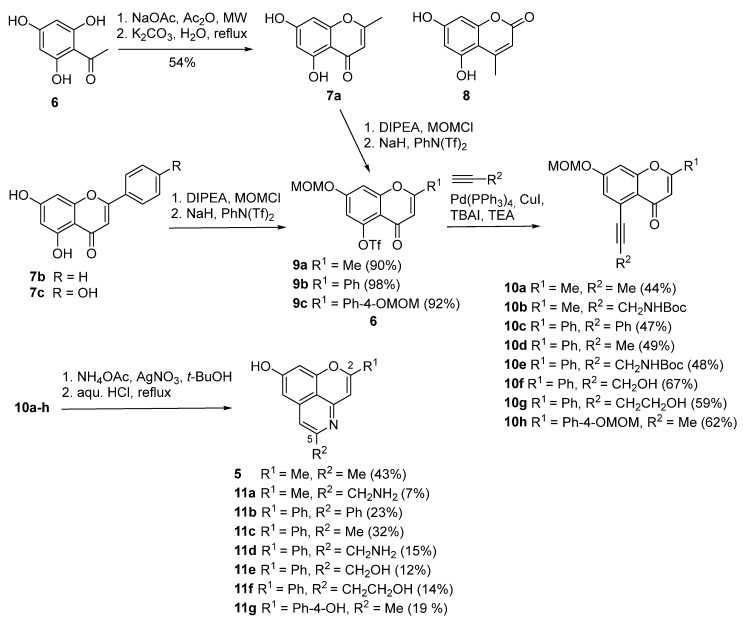
Route 1 to cassiarin A (**5**) and analogs starting from chromenones.

For Route 1, well-known 2-substituted 5,7-dihydroxychromen-4-ones were required as starting materials (Scheme 1). For the total synthesis of cassiarin A (**5**), this was noreugenin (**7a**), a natural product from *Aloe arborescens* (Amaryllidaceae), further we used commercially available 2-phenyl analog chrysin (**7b**) and 2-(4-hydroxyphenyl) analog apigenin (**7c**). Noreugenin (**7a**) was synthesized from 2,4,6-trihydroxyacetophenone (**6**) and acetic anhydride in an Allan–Robinson condensation. However, this reaction is hampered by a competing Kostanecki reaction giving coumarin **8 [27]**. We found that microwave irradiation (200 W) significantly accelerates the conversion, whereby at temperatures up to 100 °C almost equivalent amounts of **7a** and **8** were formed. Fortunately, at 180 °C, noreugenin (**7a**) was obtained as the only product in 54% yield over 2 steps (condensation and base-mediated deacylation of phenol acetates).

The dihydroxychromenones **7a**–**c** were protected regioselectively at 7-OH with MOM chloride/DIPEA, followed by triflation at 5-OH with *N*-phenyl-bis(trifluoromethanesulfonimide)/NaH following Morita’s protocol [24] to give the triflate intermediates **9a**–**c** in excellent yields. Subsequent Sonogashira coupling with five different terminal alkynes (containing methyl, phenyl, hydroxymethyl, 2-hydroxyethyl and Boc-protected aminomethyl residues) provided alkynylchromenes **10a**–**h** in yields ranging from 44% to 67%. For the final cyclization step generating the pyridine subunit of the target compounds we worked out a novel, shorter protocol. In a previous study, Yao and Yao [23] first generated and isolated isochromenylium salt intermediates, which then were cyclized with ammonia, whereas Morita [24] first converted the propynyl residue into an acetonyl group and then cyclized with an ammonium salt. Inspired by work of Reddy et al. [28] we worked out a one-pot procedure for cyclization and MOM deprotection to yield the target compounds cassiarin A (**5**) and **11a**–**g**. For the synthesis of cassiarin A (**5**), alkyne intermediate **10a** was treated under Reddy’s conditions (AgNO_3_, ammonium acetate, *tert*-butanol) for cyclization. The crude fluorescent product was separated from the inorganic components by extraction with dichloromethane/2-propanol (4:1) and after evaporation, the MOM deprotection was performed with the crude intermediate using hydrochloric acid/methanol. Without this separation step, the yield dropped drastically. Alkaloid cassiarin A (**5**) was obtained in 43% yield over both steps. The analogs **11a**–**g** were obtained in the same manner, albeit the yields were in most cases significantly lower.

**Scheme 2:**
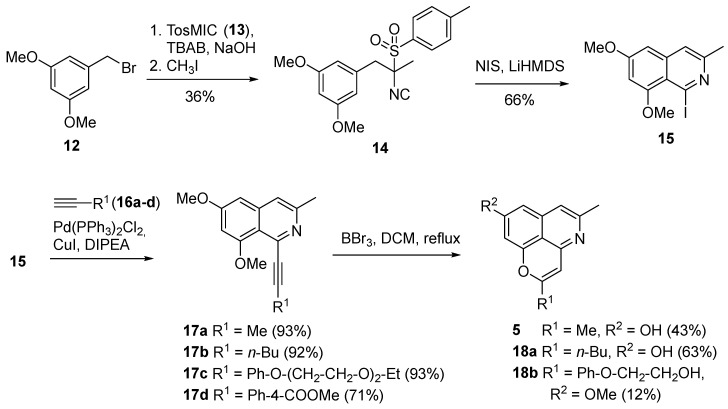
Route 2 to cassiarin A analogs via 1-iodoisoquinoline **15**.

**Scheme 3:**
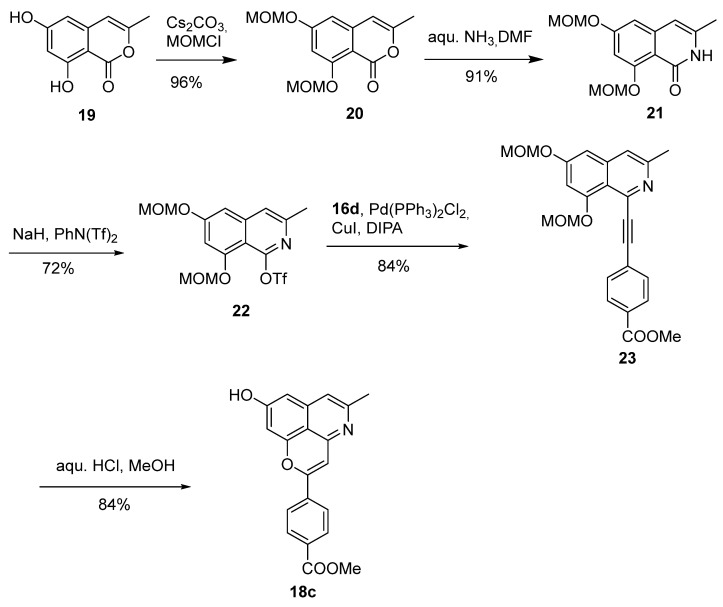
Route 3 to cassiarin A analog **18c** via isoquinoline triflate **22**.

In Route 1 the substituent at the pyran ring was introduced at a very early stage. Thus, we worked out a complementary approach to cassiarin A analogs with modifications of the methyl residue at the pyran ring which allows a late-stage introduction of diverse substituents at C-2 (Figure 2, Route 2). For this purpose, 3-methylisoquinoline intermediates bearing a leaving group (iodo, triflate) at C-1 were required. The required 1-iodoisoquinolines can be prepared starting from TosMIC (van Leusen reagent, **13**) and benzyl halides. Following this concept, the Vaquero group [25] developed a total synthesis of cassiarin A (**5**) utilizing 3,5-bis(methoxymethoxy)benzyl bromide. We found that this benzyl bromide is laborious to prepare and very unstable. Therefore, we decided to use less sensitive protective groups for the phenolic groups and ended up with cheap 3,5-dimethoxybenzyl bromide (**12**) (Scheme 2). Following Vaquero’s strategy, TosMIC (**13**) was deprotonated under phase-transfer conditions and successively alkylated with **12** and with iodomethane to give isonitrile **14** in 36% yield. Iodocyclization using *N*-iodosuccinimide and LiHMDS as a base yielded the desired 1-iodoisoquinoline **15** in 66% yield. Subsequent Sonogashira couplings with propyne (for the synthesis of cassiarin A (**5**)), 1-hexyne and 4-substituted phenylacetylenes **16d** and **16c** (prepared from 4-iodophenol by etherification and Sonogashira coupling [29]) gave the 1-alkynylisoquinolines **17a**–**d** in high yields. Cyclization to the tricyclic target compounds could not be performed under Vaquero’s conditions (hydrochloric acid) [25] due to the higher stability of the methoxy protective groups compared to the MOM group. Fortunately, treatment of intermediates **17a** and **17b** with BBr_3_ in refluxing dichloromethane gave the target compounds cassiarin A (**5**) and **18a** in 43% and 63% yields. To our surprise, arylacetylene **17c** gave a cyclized product **18b** in only 12% yield, in which one methoxy group was untouched, and part of the diethyleneglycol ether side chain was cleaved off. As an alternative *O*-demethylation reagent, we examined L-Selectride [30], but again, the only identifiable product was compound **18b**, and the yield was even lower (5%). Benzoic ester **17d** gave only a complex mixture of unidentified products under the cyclization conditions with BBr_3_, and the desired product **18c** (see Scheme 3) could not be detected.

Since obviously the BBr_3_-mediated cyclization conditions are too harsh for arylacetylene intermediates bearing ester groups, we came back to MOM protective groups for the synthesis of arene-substituted cassiarin A analog **18c** (see Scheme 3), but we avoided the use of unstable 3,5-bis(methoxymethoxy)benzyl bromide (*vide supra*) [25]. Thus, we selected isoquinoline triflate **22**, containing MOM-protected phenolic groups, as building block for the Sonogashira coupling. In contrast to Honda’s very lengthy protocol [22], we prepared the isoquinolone precursor **19** of this intermediate following Kendall’s protocol [31] starting from 3,5-dimethoxybenzaldehyde (6 steps, 36% overall yield).

MOM protection of the phenolic groups of **19**, followed by conversion of the resulting coumarine **20** into isoquinolone **21** and triflation according to Honda’s protocol gave isoquinoline triflate **22** in 63% yield over 3 steps. Sonogashira coupling of triflate **22** with phenylacetylene **16d** proceeded well (84% yield), and to our pleasure subsequent simultaneous MOM deprotection and cyclization mediated by treatment with hydrochloric acid provided the previously unavailable (see Route 2) target compound **18c** in 84% yield (Scheme 3).

**Scheme 4:**
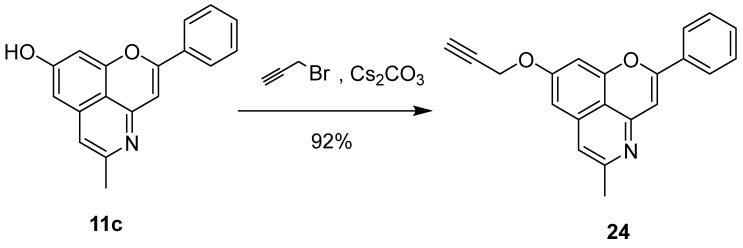
Synthesis of propargyl ether **24**.

Finally, a propargyl ether analog **24** was prepared by high-yielding etherification of the phenolic group of **11c**. This compound was designed as a potential “clickable” cassiarin A analog, in case that etherification would not eliminate antiplasmodial activity (Scheme 4).

### 2.2. Antiplasmodial Activities of Cassiarin A and Analogs

We investigated whether the 12 cassiarin-type compounds exhibited antiplasmodial activities against the chloroquine-sensitive *Plasmodium falciparum* strain 3D7 using the Malstat assay, which measures the plasmodial lactate dehydrogenase activity as a parameter for parasite viability. Overall, all compounds except propargyl ether **24** demonstrated inhibitory effects on intraerythrocytic replication of *P. falciparum*, as determined by IC_50_ values, though the required concentrations varied significantly (Table 1). Among the compounds tested, **11c**, **11d**, **11g**, and especially **18c** demonstrated the most potent inhibitory effects, with IC_50_ values ranging from 0.2 ± 0.05 µM to 0.6 ± 0.11 µM. In addition, **11b**, **11f**, **11e**, **18b**, and **18a** showed a strong inhibitory effect with IC_50_ values ranging from 1.7 ± 0.20 µM to 2.2 ± 0.58 µM. In contrast, the lead structure, alkaloid cassiarin A (**5**), had a significantly higher IC_50_ value of 9.6 ± 0.63 µM indicating lower antiplasmodial activity. Aminomethyl compound **11a** exhibited very poor potency with an IC_50_ value of 31.9 ± 11.47 µM. In accordance with previous investigations on cassiarin ethers [16], methoxy derivative **18b** showed a significant, and propargyl ether **24** complete loss of antiplasmodial activity. Additionally, the IC_90_ values for all compounds were calculated to provide a more comprehensive assessment of their inhibitory effects (Table 1).

The four most active compounds (**11c**, **11d**, **11g**, and **18c**) as well as lead compound cassiarin A (**5**), were tested against the chloroquine-resistant *P. falciparum* strain Dd2 (Table 2). All five compounds exhibited a reduced inhibitory effect compared to their efficacy against the chloroquine-sensitive strain 3D7. As expected, chloroquine showed a significantly reduced antiplasmodial effect, with the highest resistance index (RI) of 5.3 among the tested compounds (Table 3). The two compounds **11c** and **11g** showed a significantly increased RI of 4.3 and 4.8, respectively, and thus similarly high as for chloroquine. In contrast, the compounds **5**, **11d**, and **18c** exhibited a considerably lower RI, ranging from 1.4 to 2.0.

### 2.3. Human Cell Toxicity Testing of Cassiarin A and Analogs

**Figure 3:**
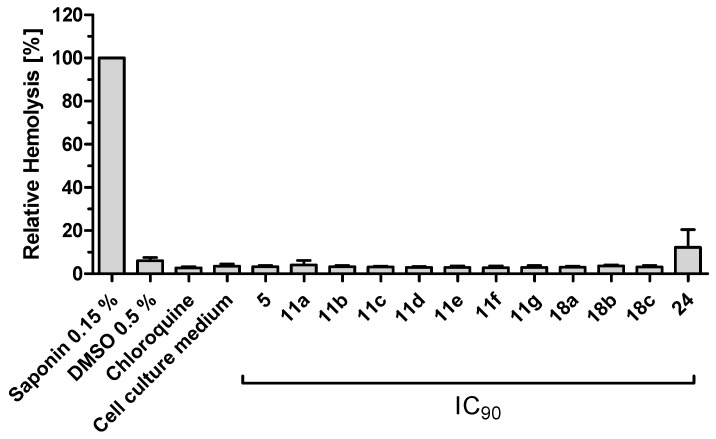
Hemolytic effect of cassiarin A and analogs. Human erythrocytes at a hematocrit of 5% were incubated with the compounds at IC_90_ concentrations for 48 h at 37 °C. Hemoglobin levels in the supernatant were subsequently measured at OD_405_ and normalized to 0.15% *w*/*v* saponin (set to 100%), which served as positive control. For the negative controls, erythrocytes were incubated with 0.5% *v*/*v* DMSO, chloroquine (IC_90_), or cell culture medium. Hemolysis was measured from three biological independent experiments (n = 3; technical triplicates; mean ± SD).

Cassiarin A (**5**) and analogs were subjected to cell toxicity assays. First, we tested potential hemolytic effects of the 12 compounds using the hemolysis assay (Figure 3). The solvent dimethyl sulfoxide (DMSO; at 0.5% *v*/*v*), chloroquine (at IC_90_ concentration) and culture media served as negative controls, the detergent saponin (at 0.15%) served as positive control in the assays. Human erythrocytes were adjusted to a hematocrit of 5% and incubated with each compound at IC_90_ concentration. After a 48 h incubation period, hemoglobin levels in the supernatant were measured spectrophotometrically and compared to controls. No increase in hemoglobin levels was observed for most of the compounds, except for **24**. Noteworthy, for **24**, a concentration of 100 µM was used, as no IC_50_ nor IC_90_ values could be determined in the Malstat assay.

**Figure 4:**
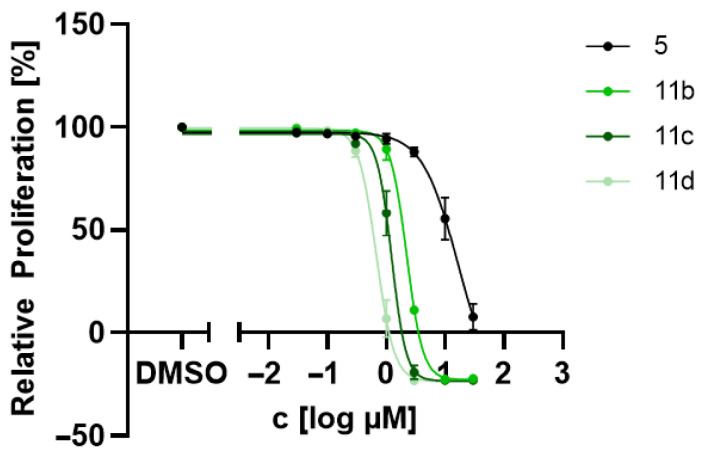
Cytotoxic effects of cassiarin A (**5**) and analogs **11b**, **11c**, and **11d** on proliferation of HUVEC assessed by CellTiter-Blue^TM^ assay. IC_50_ values were determined using the log(inhibitor) vs. response—variable slope (four parameters) function of GraphPad Prism (10.4.0). Data are shown as mean ± SD.

Further, we selected four compounds, cassiarin A (**5**), **11b**, **11c** and **11d**, for testing on the human epithelial cell line HUVEC (human umbilical vein endothelial cells) to assess potential cytotoxicity. Unfortunately, the obtained IC_50_ values (16.4 µM for **5**, 2.22 µM for **11b**, 1.23 µM for **11c**, 0.68 µM for **11d**) were in the same concentration range (**11b**, **11d**) or only factor 2 (cassiarin A) or factor 3 (**11c**) higher than the IC_50_ values of these compounds against *P. falciparum* 3D7 cells, indicating significant cytotoxicity at the respective IC_50_ concentrations (Figure 4). In contrast, for chloroquine, a selectivity index (=IC_50_ (HUVEC)/IC_50_ (3D7)) of 1987 has been reported [32]. Hence, despite enhanced anti-malarial activity, the potential therapeutic window is too small for further development of this compound class as drug candidates.

## 3. Discussion

The rapid development of resistance to drugs, even with the use of combination therapies, poses a significant challenge in the fight against malaria. Consequently, it is crucial to develop new classes of active substances. The natural product cassiarin A (**5**), extracted from leaves of *Cassia siamea*, has demonstrated strong antiplasmodial activity, prompting us to investigate its structure–activity relationships using novel analogs synthesized on three different synthetic routes. Cassiarin A and its analogs were first tested for their antiplasmodial activities, using the chloroquine-sensitive *P. falciparum* strain 3D7. Especially, changes in the methyl groups at C-2 and C-5 significantly influenced the antiplasmodial effect. The introduction of a phenyl group at C-2 (compound **11c**) led to a marked increase in antiplasmodial activity compared to cassiarin A, and 4-substituted phenyl substituents (phenol **11g**, ester **18c**) showed activities in the same range. Aromatic residues were found to have a significantly better impact than alkyl residues (*n*-butyl analog **18a**). On the other side, variations in the methyl group at C-5 led to a significant (compound **11a**) or (in combination with a 5-phenyl residue) to a marked decrease in activity (compounds **11d**–**f**). Further, *O*-alkylation of the phenolic group was found to be detrimental to antiplasmodial activity.

The four most effective compounds, **11c**, **11d**, **11g**, and **18c**, were further tested against the chloroquine-resistant strain Dd2. This strain exhibits resistance to chloroquine due to eight point mutations *pfcrt*, which results in reduced accumulation of chloroquine in the food vacuole as described above. Our results indicated a resistance index (RI) of 5.3 for chloroquine between 3d7 and Dd2. Compounds **11c** and **11g** had a similarly high RI of 4.3 and 4.8, respectively, while cassiarin A had a lower RI of 1.4, and both **11d** and **18c** exhibited RIs of 2.0, indicating better preservation of the antiplasmodial effect.

Further, cassiarin A (**5**) and analogs were subjected to cell toxicity assays. In a hemolysis assay, the 12 compounds did not show noteworthy hemolytic activity. For obtaining a better insight into the toxicity profile, we also tested the most active compounds on a healthy human epithelial cell line. A literature search revealed that in previous investigations on antiplasmodial cassiarin derivatives this aspect was either ignored, or only tumour cell lines (MCF7 [24], P388 [11]) were used, resulting in acceptable values. Unfortunately, in a test for potential cytotoxicity on the human cell line HUVEC (human umbilical vein endothelial cells), the selected compounds cassiarin A (**5**) **11b**, **11c**, and **11d** showed significant cytotoxicity at the respective IC_50_ concentrations. To assess cytotoxicity in more detail, additional tests on cytotoxicity towards blood cells (e.g., peripheral blood mononuclear cells) could be performed. Furthermore, the toxicity on liver cells and blood half-life times could provide additional information. These tests were not performed on the basis that for the present data we do not estimate cassiarin A and analogs as promising drug candidates.

## 4. Materials and Methods

### 4.1. Chemistry

#### 4.1.1. General Reagent and Analytical Information

Solvents were purified according to standard procedures or purchased in high purity from commercial sources. Reagents were purchased from BLD Pharm (Kaiserslautern, Germany), Sigma Aldrich (Schnelldorf, Germany), or TCI (Eschborn, Germany). IR spectra were recorded on a Perkin Elmer (Waltham, MA, USA) FTIR Paragon 1000 spectrometer, NMR spectra on a Jeol (Tokyo, Japan) JNMR-GX 400 (400 MHz), Jeol JNMR-GX 500 (500 MHz), Avance III HD Bruker Bio-Spin (400 MHz) (Bruker, Billerica, MA, USA), or Avance III HD Bruker BioSpin (500 MHz) spectrometer. Signal assignments were carried out based on ^1^H, ^13^C, DEPT, HMQC, HMBC, and COSY spectra. Chemical shifts are reported in ppm (parts per million) and coupling constants (J) in Hertz. High resolution mass spectra were recorded by means of electrospray ionization (ESI) using a Thermo Finnigan (San Josa, CA, USA) LTQ FT Ultra spectrometer or by electron impact (EI) ionization at 70 eV on a Jeol GCmate II or a Finnigan MAT 95 spectrometer. Separations of crude products by flash column chromatography (FCC) were performed on Merck silica gel 60. Melting points were determined on a Büchi melting point B-540 apparatus and are uncorrected. HPLC purities were determined using an HP Agilent 1100 HPLC apparatus (Agilent, Waldbronn, Germany) with a diode array detector (detection wavelengths 210 nm and 254 nm) with an Agilent Zorbax Eclipse plus C18 column (150 × 4.6 mm; 5 μm) using acetonitrile/water in different proportions as the mobile phase.

#### 4.1.2. Syntheses

##### General Procedure A (Synthesis of 5-Alkynyl-4*H*-chromen-4-ones Via Sonogashira Reaction)

The required triflate (**9a**–**c**, 2.00 mmol), tetrabutylammonium iodide (2.22 g, 6.00 mmol), copper(I)iodide (114 mg, 0.600 mmol) and tetrakis(triphenylphosphane)palladium(0) (231 mg, 0.200 mmol) were placed in a Schlenk flask under nitrogen and the flask closed with a rubber septum. Then, a solution of the corresponding alkyne (2.40 mmol) in DMF/triethylamine (2:1) (15 mL) was added and the mixture was stirred at ambient temperature for 16 h. Then, satd. ammonium chloride solution (80 mL) was added, followed by extraction with ethyl acetate (5 × 30 mL). The combined organic layers were washed with brine (2 × 25 mL), dried over sodium sulphate and concentrated in vacuo. The crude product was purified by FCC with the indicated eluent.

##### General Procedure B (Construction of the Cassiarin A Backbone)

The required alkyne (**10a**–**h**, 0.50 mmol), silver nitrate (8.5 mg, 0.050 mmol) and ammonium acetate (58 mg, 0.75 mmol) were suspended in 3 mL *tert*-butanol and the mixture stirred for 16 h at 40 °C. Water (15 mL) was added, followed by extraction with 2 × 10 mL dichloromethane/2-propanol (4:1). The combined organic layers were dried over sodium sulphate and concentrated in vacuo. The residue was treated with 10 mL of a 1:1 mixture of methanol and 6M hydrochloric acid and refluxed for 5 h. After cooling and neutralization with 2 M sodium hydroxide solution the mixture was extracted with dichloromethane/2-propanol (4:1, 6 × 5 mL). The combined organic layers were dried over sodium sulphate and concentrated in vacuo. The crude product was purified by FCC with the indicated eluent.

##### General Procedure C (Synthesis of 1-Alkynylisoquinolines Via Sonogashira Reaction)

1-Iodo-6,8-dimethoxy-3-methylisoquinoline (**15**; 200 mg, 0.608 mmol), bis(triphenylphosphane)palladium(II) dichloride (21.3 mg, 0.0304 mmol) and copper(I)iodide (11.6 mg, 0.0608 mmol) were placed in a 50 mL Schlenk flask and nitrogen and the flask closed with a rubber septum. Then, a solution of the corresponding alkyne (**16a**–**d**, 1.22 mmol) in diisopropylamine (3 mL) was added and the mixture was stirred at ambient temperature for 18 h. Then, satd. ammonium chloride solution (25 mL) was added, followed by extraction with dichloromethane (3 × 10 mL). The combined organic layers were washed with brine (2 × 10 mL), dried over sodium sulphate and concentrated in vacuo. The crude product was purified by FCC with the indicated eluent.

*Cassiarin A (**5**).* **Method 1:** Prepared according to General Procedure B using 129 mg (0.500 mmol) 7-(methoxymethoxy)-2-methyl-5-(prop-1-yn-1-yl)-4*H*-chromen-4-one (**10a**). Purification by FSC (dichloromethane/methanol 10:1). Yield: 0.45 g (0.21 mmol, 42%) yellow solid.

**Method 2:** A solution of 6,8-dimethoxy-3-methyl-1-(prop-1-yn-1-yl)isoquinoline (**17a**) (121 mg, 0.500 mmol) in 7 mL dichloromethane in a Schlenk flask under nitrogen was treated slowly Via a syringe with boron tribromide (1.00 g, 4.00 mmol) and the solution then heated under reflux for 6 h. After cooling to ambient temperature 6 mL methanol and 5 mL aqueous ammonia (10%) was added, and the mixture was stirred vigorously for 15 min. The mixture was evaporated to dryness in vacuo and the residue was purified by FSC (dichloromethane/methanol 10:1). Yield: 46 mg (0.215 mmol, 43%).

^1^H-NMR (400 MHz, (CD_3_)_2_SO): *δ* 11.01 (br s, 1 H, OH), 6.96 (s, 1H, 6-H), 6.68 (d, *J* = 2.0 Hz, 1H, 7-H or 9-H), 6.67 (d, *J* = 2.0 Hz, 1H, 7-H or 9-H), 6.35 (s, 1H, 3-H), 2.36 (s, 3H, 5-CH_3_), 2.29 (s, 3H, 2-CH_3_). ^13^C-NMR (101 MHz, (CD_3_)_2_SO) *δ* 162.9 (C-2), 155.2 (C-8), 148.9 (C-5), 147.2 (C-9a), 137.8 (C-6a), 113.2 (C-6), 110.2 (C-3a^1^), 102.3 (C-7, C-3), 99.8 (C-9), 21.5 (5-CH_3_), 19.8 (2-CH_3_). IR (ATR): *ṽ* (cm^−1^) 2759, 1652, 1606, 1431, 1392, 1367, 1291, 1188, 1168, 1134, 1083, 919, 831, 816. HRMS (EI): calcd. for [C_13_H_11_NO_2_]^+•^: 213.0790, found: 213.0775. M.p.: 254 °C (decomp.) (published: 240 °C, decomp. [22]). HPLC purity: >99% (210 nm), >99% (254 nm).

*5,7-Dihydroxy-2-methyl-4H-chromen-4-one (noreugenine) (**7a**).* 2,4,6-Trihydroxyacetophenone (**6**; 2.02 g, 12.0 mmol) and sodium acetate (12.8 g, 144 mmol) were mixed with 6 mL acetic anhydride in a microwave vessel. The vessel was closed with a septum and irradiated for 40 min (p_max_ = 8 bar, P = 200 W, T = 180 °C). Then, the mixture was treated with 100 mL water and extracted with 4 × 25 mL ethyl acetate. The combined organic layers were washed with 25 mL brine, dried over sodium sulphate and evaporated. The residue was suspended in 90 mL water and treated with potassium carbonate (4.98 g, 30.1 mmol) under reflux for 3 h. After cooling, the mixture was neutralized with hydrochloric acid and extracted with 3 × 25 mL ethyl acetate. The combined organic layers were washed with 25 mL brine, dried over sodium sulphate and evaporated. The residue was purified by FCC (isohexane/ethyl acetate 7:3). Yield: 1.25 g (6.51 mmol, 54%) pale yellow solid. ^1^H-NMR (400 MHz, (CD_3_)_2_SO) *δ* 12.81 (s, 1H, 7-OH), 10.78 (s, 1H, 5-OH), 6.29 (d, *J* = 2.1 Hz, 1H, 8-H), 6.15 (d, *J* = 2.1 Hz, 1H, 6-H), 6.13 (d, *J* = 0.9 Hz, 1H, 3-H), 2.32 (d, *J* = 0.8 Hz, 3H, CH_3_). ^13^C-NMR (101 MHz, (CD_3_)_2_SO) *δ* 181.8 (C-4), 167.6 (C-2), 164.1 (C-7), 161.5 (C-5), 157.8 (C-8a), 108.0 (C-3), 103.4 (C-4a), 98.8 (C-6), 93.7 (C-8), 19.9 (CH_3_). IR (ATR): *ṽ* (cm^−1^) 3086, 1765, 1613, 1366, 1189, 1101, 1068, 1022, 828, 723. HRMS (EI): calcd. for [C_10_H_8_O_4_]^+•^: 192.0417), found: 192.0415. M.p.: 280 °C.

*7-(Methoxymethoxy)-2-methyl-4-oxo-4H-chromen-5-yl trifluoromethanesulfonate (**9a**).* To an ice-cooled solution of 5-hydroxy-7-(methoxymethoxy)-2-methyl-4*H*-chromen-4-one (1.57 g, 6.65 mmol) in 35 mL THF was added 399 mg (9.98 mmol) sodium hydride (60% dispersion in paraffin) and the mixture was stirred at 0 °C for 15 min. To the resulting mixture was added dropwise a solution of 3.56 g (9.98 mmol) *N*-phenyl-bis-(trifluoromethanesulfonimide) in 10 mL THF and the mixture was stirred at ambient temperature for 2 h. Then, a satd. solution of ammonium chloride (50 mL) was added under vigorous stirring, followed by extraction with 3 × 30 mL ethyl acetate. The combined organic layers were washed with 25 mL brine, dried over sodium sulphate and evaporated. The residue was purified by FCC (isohexane/ethyl acetate 5:1). Yield: 2.29 g (6.22 mmol, 94%) pale yellow solid. ^1^H-NMR (400 MHz, CDCl_3_) *δ* 7.11 (d, *J* = 2.1 Hz, 1H), 6.85 (d, *J* = 1.8 Hz, 1H), 6.10 (s, 1H), 5.27 (s, 2H), 3.51 (s, 3H), 2.35 (s, 3H). ^13^C-NMR (101 MHz, CDCl_3_) *δ* 178.5 (C-4), 167.6 (C-2), 163.2 (C-7), 158.0 (C-8a), 146.7 (C-5), 117.9 (CF_3_), 112.3 (C-4a), 108.5 (C-3), 104.9 (C-6), 99.9 (C8), 94.2 (OCH_2_O), 56.1 (OCH_3_), 19.5 (CH_3_). IR (ATR): *ṽ* (cm^−1^) 2560, 1632, 1556, 1424, 1398, 1336, 1211, 1182, 1138, 1019, 1003, 864, 820. HRMS (EI): calcd. for [C_13_H_11_F_3_O_7_S]^+•^: 368.0172, found: 368.0171. M.p.: 112 °C).

*7-(Methoxymethoxy)-4-oxo-2-phenyl-4H-chromen-5-yl trifluoromethanosulfonate (**9b**).* A solution of chrysine (**7b**; 4.00 g, 15.7 mmol) in 90 mL DMF was treated with DIPEA (4.68 g, 36.2 mmol) and stirred at 0 °C for 15 min. Then, chloromethyl methyl ether (1.52 g, 1.43 mL, 18.9 mmol) was slowly added by a syringe and the solution stirred at ambient temperature for 7 h. Then, 150 mL water was added, followed by extraction with 3 × 100 mL ethyl acetate. The combined organic layers were washed with 2 × 100 mL brine, dried over sodium sulphate and evaporated. The residue was dissolved in 50 mL THF, cooled to 0 °C, and treated with 942 mg (23.5 mmol) sodium hydride (60% dispersion in paraffin) and stirred at 0 °C for 15 min. To the resulting mixture was added dropwise a solution of 8.41 g (23.5 mmol) *N*-phenyl-bis-(trifluoromethanesulfonimide) in 20 mL THF and the mixture was stirred at ambient temperature for 3 h. Then, a satd. solution of ammonium chloride (150 mL) was added under vigorous stirring, followed by extraction with 3 × 100 mL ethyl acetate. The combined organic layers were washed with 2 × 70 mL brine, dried over sodium sulphate and evaporated. The residue was purified by FCC (isohexane/ethyl acetate 5:1). Yield: 6.60 g (15.4 mmol, 98%) yellow solid. ^1^H-NMR (400 MHz, (CD_3_)_2_SO) *δ* 8.12 (dt, *J* = 7.5, 1.1 Hz, 2H, 2′-H, 6′H), 7.68–7.54 (m, 4H, 8-H, 3′-H, 4′-H, 5′-H), 7.17–7.11 (m, 1H, 6-H), 7.01 (s, 1H, 3-H), 5.43 (s, 2H, CH_2_), 3.45 (s, 3H, CH_3_). ^13^C-NMR (101 MHz, (CD_3_)_2_SO) *δ* 175.4 (C-4), 162.4 (C-2), 160.8 (C-7), 158.2 (C-8a), 147.2 (C-5), 132.6 (C-4′), 130.8 (C-1′), 129.6 (C-3′, C-5′), 126.9 (C-2′, C-6′), 117.2 (CF_3_), 111.9 (C-4a), 109.9 (C-6), 108.1 (C-3), 105.3 (C-8), 95.1 (CH_2_), 56.8 (CH_3_). IR (ATR): *ṽ* (cm^−1^) 2925, 1637, 1609, 1427, 1346, 1216, 1147, 1023, 974, 927, 846, 819, 772. HRMS (EI): calcd. for [C_21_H_28_N_2_O_2_Si]^+•^: 430.0329, found: 430.0328. M.p.: 120 °C.

*5-Hydroxy-7-(methoxymethoxy)-2-(4-(methoxymethoxy)phenyl)-4H-chromen-4-one (**Bis-MOM-7c**).* To an ice-cooled solution of apigenin (**7c**; 4.05 g, 15.0 mmol) in 50 mL DMF was added DIPEA (5.88 g, 45.0 mmol). The solution was stirred for 10 min, then 2.85 mL (3.02 g, 37.5 mmol) chloromethyl methyl ether was added, the flask was closed and the mixture stirred at 0 °C for 2 h. Then, 60 mL 1M hydrochloric acid was added slowly under ice-cooling and the mixture extracted with 3 × 50 mL ethyl acetate. The combined organic layers were washed with 30 mL brine, died over sodium sulphate and evaporated. The residue was purified by FCC (isohexane/ethyl acetate 3:1). Yield: 5.32 g (14.9 mmol, 99%) colourless solid. ^1^H-NMR (400 MHz, (CD_3_)_2_SO) *δ* 12.89 (s, 1H, OH), 8.12–8.03 (m, 2H, 2′-H, 6′-H), 7.24–7.16 (m, 2H, 3′-H, 5′-H), 6.96 (s, 1H, 3-H), 6.85 (d, *J* = 2.2 Hz, 1H, 8-H), 6.46 (d, *J* = 2.1 Hz, 1H, 6-H), 5.33 (s, 2H, CH_2_), 5.32 (s, 2H, CH_2_), 3.42 (s, 3H, CH_3_), 3.41 (s, 3H, CH_3_). ^13^C-NMR (101 MHz, (CD_3_)_2_SO) *δ* 182.1 (C-4), 163.6 (C-2), 162.5 (C-7), 161.1 (C-5), 159.9 (C-8a), 157.0 (C-4′), 128.4 (C-2′, C-6′), 123.7 (C-1′), 116.4 (C-3′, C-5′), 105.3 (C-4a), 104.0 (C-3), 99.5 (C-6), 94.6 (C-8), 93.9 (CH_2_), 93.7 (CH_2_), 56.1 (CH_3_), 55.9 (CH_3_). IR (ATR): *ṽ* (cm^−1^) 2956, 1643, 1612, 1513, 1424, 1375, 1207, 1150, 1139, 1088, 1078, 1010, 999, 986, 927, 858, 819. HRMS (EI): calcd. for [C_19_H_18_O_7_]^+•^: 358.1047, found: 358.1048. M.p.: 105 °C.

*7-(Methoxymethoxy)-2-(4-(methoxymethoxy)phenyl)-4-oxo-4H-chromen-5-yl trifluoromethanesulfonate (**9c**).* Prepared in the same manner as described above for compound **9b** starting from 5-hydroxy to 7-(methoxymethoxy)-2-(4-(methoxymethoxy)phenyl)-4*H*-chromen-4-one (**Bis-MOM-7c***,* 2.00 g, 5.57 mmol) using 34 mg (8.36 mmol) sodium hydride (60% dispersion in paraffin) and 2.99 g (8.36 mmol) *N*-phenyl-bis-(trifluoromethanesulfonimide). The residue was purified by FCC (isohexane/ethyl acetate 5:1). Yield: 2.55 g (5.20 mmol, 93%) colourless solid. ^1^H-NMR (400 MHz, (CD_3_)_2_SO) *δ* 8.12–8.02 (m, 2H, 2′-H, 6′-H), 7.56 (d, *J* = 2.3 Hz, 1H, 8-H), 7.24–7.16 (m, 2H, 3′-H, 5′-H), 7.11 (d, *J* = 2.3 Hz, 1H, 6-H), 6.91 (s, 1H, 3-H), 5.43 (s, 2H, OCH_2_O), 5.31 (s, 2H, OCH_2_O), 3.44 (s, 3H, OCH_3_), 3.41 (s, 3H, OCH_3_). ^13^C-NMR (126 MHz, (CD_3_)_2_SO) *δ* 174.7 (C-4), 161.9 (C-2), 160.1 (C-7), 159.8 (C-4′), 157.6 (C-8a), 146.7 (C-5), 128.3 (C-2′, C-6′), 123.4 (C-1′), 116.4 (C-3′, C-5′), 111.3 (C-4a), 109.3 (C-6), 106.4 (C-3), 104.8 (C-8), 94.6 (OCH_2_O), 93.7 (OCH_2_O), 56.3 (OCH_3_), 55.9 (OCH_3_). IR (ATR): *ṽ* (cm^−1^) 3092, 2967, 2843, 2785, 1968, 1789, 1325, 1222, 1104. 1050, 981. HRMS (EI): calcd. for [C_20_H_17_F_3_O_9_S]^+•^: 490.0613, found: 491.0609. M.p.: 131 °C.

*7-(Methoxymethoxy)-2-methyl-5-(prop-1-yn-1-yl)-4H-chromen-4-one (**10a**).* Prepared according to General Procedure A using 737 mg (2.00 mmol) 7-(methoxymethoxy)-2-methyl-4-oxo-4*H*-chromen-5-yl trifluormethanesulfonate (**9a**) and propyne (1 M in DMF, 2.4 mL, 2.4 mmol). Purification by FSC (isohexane/ethyl acetate 2:1). Yield: 225 mg (0.871 mmol, 44%) orange-red solid. ^1^H-NMR (400 MHz, CDCl_3_) *δ* 7.10 (d, *J* = 2.5 Hz, 1H, 6-H), 6.94 (d, *J* = 2.5 Hz, 1H, 8-H), 6.04 (d, *J* = 0.9 Hz, 1H, 3-H), 5.23 (s, 2H, CH_2_), 3.49 (s, 3H, OCH_3_), 2.29 (d, *J* = 0.7 Hz, 3H, CH_3_), 2.17 (s, 3H, 3′-H). ^13^C-NMR (101 MHz, CDCl_3_) *δ* 177.2 (C-4), 164.3 (C-2), 159.9 (C-7), 158.7 (C-8a), 124.5 (C-5), 120.9 (C-6), 118.7 (C-4a), 111.4 (C-3), 103.5 (C-8), 94.4 (CH_2_), 92.8 (C-2′), 78.8 (C-1′), 56.5 (OCH_3_), 20.2 (CH_3_), 5.3 (C-3′). IR (ATR): *ṽ* (cm^−1^) 1654, 1601, 1391, 1338, 1156, 1078. HRMS (EI): calcd. for [C_15_H_14_O_4_]^+•^: 258.0887, found: 58.0888. M.p.: 152 °C (decomp.).

*tert-Butyl (3-(7-(methoxymethoxy)-2-methyl-4-oxo-4H-chromen-5-yl)prop-2-in-1-yl)carbamate (**10b**).* Prepared according to General Procedure A using 737 mg (2.00 mmol) 7-(methoxymethoxy)-2-methyl-4-oxo-4*H*-chromen-5-yl trifluormethanesulfonate (**9a**) and 372 mg (2.40 mmol) *N*-(*tert*-butoxycarbonyl)propargylamine. Purification by FSC (isohexane/ethyl acetate 1:1) gave an impure product (512 mg, 1.37 mmol, 69%, black gum), which was directly converted into free amine **11a**.

*7-(Methoxymethoxy)-2-phenyl-5-(phenylethynyl)-4H-chromen-4-one (**10c**).* Prepared according to General Procedure A using 861 mg (2.00 mmol) 7-(methoxymethoxy)-4-oxo-2-phenyl-4*H*-chromen-5-yl trifluoromethanesulfonate (**9b**) and 0.264 mL (245 mg, 2.40 mmol) phenylacetylene. Purification by FSC (isohexane/ethyl acetate 3:1). Yield: 358 mg (0.936 mmol, 47%) yellow solid. ^1^H-NMR (400 MHz, CDCl_3_) *δ* 7.95–7.86 (m, 2H, 2′-H, 6′-H), 7.74–7.67 (m, 2H, 4″-H, 8″-H), 7.52 (dd, *J* = 5.3, 2.0 Hz, 3H, 3′-H, 4′-H, 5′-H), 7.42–7.31 (m, 3H, 5″-H, 6″-H, 7″-H), 7.26 (d, *J* = 2.3 Hz, 1H, 6-H), 7.17 (d, *J* = 2.4 Hz, 1H, 8-H), 6.74 (s, 1H, 3-H), 5.30 (s, 2H, CH_2_), 3.53 (s, 3H, OCH_3_). ^13^C-NMR (101 MHz, CDCl_3_) *δ* 177.2 (C-4), 161.8 (C-2), 160.1 (C-7), 158.4 (C-8a), 132.1 (C-4″, C-8″), 131.5 (C-1′), 131.5 (C-4′), 129.0 (C-3′, C-5′), 128.7 (C-6″), 128.3 (C-5″, C-7″), 126.1 (C-2′, C-6′), 123.6 (C-5), 123.4 (C-3″), 120.6 (C-6), 118.9 (C-4a), 108.4 (C-3), 104.0 (C-8), 95.3 (C-2″), 94.4 (CH_2_), 88.6 (C-1″), 56.5 (OCH_3_). IR (ATR): *ṽ* (cm^−1^) 3056, 1646, 0595, 1374, 1346, 1229, 1151, 1071, 1016, 833, 760. HRMS (EI): calcd. for [C_25_H_18_O_4_]^+•^: 382.1200, found: 382.1198. M.p.: 141 °C.

*7-(Methoxymethoxy)-2-phenyl-5-(prop-1-yn-1-yl)-4H-chromen-4-one (**10d**).* Prepared according to General Procedure A using 861 mg (2.00 mmol) 7-(methoxymethoxy)-4-oxo-2-phenyl-4*H*-chromen-5-yl trifluoromethanesulfonate (**9a**) and propyne (1M in DMF, 2.4 mL, 2.4 mmol). Purification by FSC (isohexane/ethyl acetate 2:1). Yield: 313 mg (0.976 mmol, 49%) orange solid (plus recovered starting material, 323 mg, 0.751 mmol). ^1^H-NMR (400 MHz, (CD_3_)_2_SO) *δ* 8.13–8.04 (m, 2H, 2′-H, 6′-H), 7.60–7.56 (m, 3H, 3′-H, 4′-H, 5′-H), 7.35 (d, *J* = 2.4 Hz, 1H, 8-H), 7.10 (d, *J* = 2.4 Hz, 1H, 6-H), 6.88 (s, 1H, 3-H), 5.36 (s, 2H, CH_2_), 3.42 (s, 3H, OCH_3_), 2.11 (s, 3H, 3″-H). ^13^C-NMR (101 MHz, (CD_3_)_2_SO) *δ* 175.6 (C-4), 160.8 (C-2), 159.6 (C-7), 157.8 (C-8a), 131.7 (C-4′), 130.8 (C-1′), 129.1 (C-3′, C-5′), 126.2 (C-2′, C-6′), 123.2 (C-5), 120.4 (C-6), 118.1 (C-4a), 107.5 (C-3), 104.1 (C-8), 94.0 (CH_2_), 92.5 (C-2″), 78.8 (C-1″), 56.1 (OCH_3_), 4.7 (C-3″). IR (ATR): *ṽ* (cm^−1^) 2912, 1647, 1597, 1375, 1342, 1164, 1076, 991, 917, 843, 763. HRMS (EI): calcd. for [C_20_H_17_O_4_]^+•^: 321.1121, found: 321.1122. M.p.: 123 °C.

*tert-Butyl (3-(7-(methoxymethoxy)-4-oxo-2-phenyl-4H-chromen-5-yl)prop-2-yn-1-yl)carbamate (**10e**).* Prepared according to General Procedure A using 861 mg (2.00 mmol) 7-(methoxymethoxy)-4-oxo-2-phenyl-4*H*-chromen-5-yl trifluoromethanesulfonate (**9b**) and 372 mg (2.40 mmol) *N*-(*tert*-butoxycarbonyl)propargylamine. Purification by FSC (isohexane/ethyl acetate 1:1). Yield: 420 mg (0.964 mmol, 48%) orange-red solid. ^1^H-NMR (500 MHz, (CD_3_)_2_SO) *δ* 8.10 (dt, *J* = 6.5, 1.7 Hz, 2H, 2′-H, 6′-H), 7.59 (tdd, *J* = 8.8, 7.3, 4.7 Hz, 3H, 3′-H, 4′-H, 5′-H), 7.40 (d, *J* = 2.4 Hz, 1H, 8-H), 7.35 (d, *J* = 6.2 Hz, 1H, NH), 7.12 (d, *J* = 2.4 Hz, 1H, 6-H), 6.90 (s, 1H, 3-H), 5.38 (s, 2H, OCH_2_O), 4.06 (d, *J* = 5.7 Hz, 2H, 3″-H), 3.43 (s, 3H, OCH_3_), 1.42 (s, 9H, *tert*-butyl). ^13^C-NMR (126 MHz, (CD_3_)_2_SO) *δ* 176.0 (C-4), 161.5 (C-2), 160.1 (C-7), 158.3 (C-8a), 155.8 (N-C=O), 132.2 (C-4′), 131.2 (C-1′), 129.6 (C-3′, C-5′), 126.7 (C-2′, C-6′), 122.7 (C-5), 121.1 (C-6), 118.5 (C-4a), 108.0 (C-3), 105.1 (C-8), 94.5 (OCH_2_O), 93.5 (C-2″), 81.2 (C-1″), 78.8 (C-7″), 31.1 (C-3″), 28.7 (3 CH_3_, *tert*-butyl). IR (ATR): *ṽ* (cm^−1^) 338, 1681, 1648, 1598, 1520, 1377, 1342, 1266, 1249, 1161, 1148, 1080, 1045, 992, 950, 844, 764. HRMS (EI): calcd. for [C_25_H_26_NO_6_]^+•^: 436.1755, found: 436.1763. M.p.: 156 °C.

*5-(3-Hydroxyprop-1-yn-1-yl)-7-(methoxymethoxy)-2-phenyl-4H-chromen-4-one (**10f**).* Prepared according to General Procedure A using 861 mg (2.00 mmol) 7-(methoxymethoxy)-4-oxo-2-phenyl-4*H*-chromen-5-yl trifluoromethanesulfonate (**9b**) and 0.142 mL (135 mg, 2.40 mmol) propargyl alcohol. Purification by FSC (isohexane/ethyl acetate 2:1). Yield: 451 mg (1.34 mmol, 67%) orange solid. ^1^H-NMR (400 MHz, (CD_3_)_2_SO) *δ* 8.09 (dd, *J* = 8.1, 1.7 Hz, 2H, 2′-H, 6′-H), 7.61–7.55 (m, 3H, 3′-H, 4′-H, 5′-H), 7.40 (d, *J* = 2.4 Hz, 1H, 8-H), 7.14 (d, *J* = 2.4 Hz, 1H, 6-H), 6.90 (s, 1H, 3-H), 5.38 (s, 2H, OCH_2_O), 4.37 (d, *J* = 4.8 Hz, 2H, CH_2_), 3.44 (s, 1H, OH), 3.43 (s, 3H, CH_3_). ^13^C-NMR (101 MHz, (CD_3_)_2_SO) *δ* 176.0 (C-4), 161.4 (C-2), 160.1 (C-7), 158.3 (C-8a), 132.2 (C-4′), 131.2 (C-1′), 129.6 (C-3′, C-5′), 126.7 (C-2′, C-6′), 122.8 (C-5), 121.0 (C-6), 118.4 (C-4a), 108.0 (C-3), 105.2 (C-8), 95.7 (C-2″), 94.5 (OCH_2_O), 83.1 (C-1″), 56.6 (CH_3_), 50.3 (C-3″). IR (ATR): *ṽ* (cm^−1^) 3272, 1645, 1595, 1451, 1377, 1344, 1150, 1102, 1075, 1045, 1015, 916, 842, 763. HRMS (EI): calcd. for [C_16_H_10_IN_3_O_4_]^+•^: 336.0992, found: 336.0987. M.p.: 290 °C

*5-(4-Hydroxybut-1-yn-1-yl)-7-(methoxymethoxy)-2-phenyl-4H-chromen-4-one (**10g**).* Prepared according to General Procedure A using 861 mg (2.00 mmol) 7-(methoxymethoxy)-4-oxo-2-phenyl-4*H*-chromen-5-yl trifluoromethanesulfonate (**9b**) and 0.187 mL (173 mg, 2.40 mmol) but-3-yn-1-ol. Purification by FSC (isohexane/ethyl acetate 2:1). Yield: 412 mg (1.18 mmol, 59%) orange solid. ^1^H-NMR (400 MHz, (CD_3_)_2_SO) *δ* 7.96–7.79 (m, 2H, 2′-H, 6′-H), 7.57–7.48 (m, 3H, 3′-H, 4′-H, 5′-H), 7.07 (d, *J* = 1.6 Hz, 1H, 6-H), 6.93 (d, *J* = 1.6 Hz, 1H, 8-H), 6.73 (s, 1H, 3-H), 5.18 (s, 2H, OCH_2_O), 3.77 (td, *J* = 7.1, 5.7 Hz, 2H, 4″-H), 3.51 (s, 3H, OCH_3_), 2.61 (t, *J* = 7.1 Hz, 2H, 3″-H). ^13^C-NMR (101 MHz, (CD_3_)_2_SO) *δ* 179.6 (C-4), 161.9 (C-2), 161.0 (C-7), 155.4 (C-8a), 131.6 (C-4′), 131.1 (C-1′), 129.0 (C-3′, C-5′), 126.6 (C-2′, C-6′), 125.6 (C-5), 121.2 (C-4a), 115.8 (C-6), 107.4 (C-3), 106.7 (C-8), 94.3 (OCH_2_O), 86.6 (C-2″), 81.3 (C-1″), 60.1 (C-4″), 56.1 (OCH_3_), 23.6 (C-3″). IR (ATR): *ṽ* (cm^−1^) 3448, 1940, 1636, 1598, 1382, 1347, 1152, 1075, 1045, 993, 920, 767. HRMS (EI): calcd. for [C_21_H_18_O_5_]^+•^: 350.1149, found: 350.1150. M.p.: 111 °C.

*7-(Methoxymethoxy)-2-(4-(methoxymethoxy)phenyl)-5-(prop-1-yn-1-yl)-4H-chromen-4-one (**10h**).* Prepared according to General Procedure A using 981 mg (2.00 mmol) 7-(methoxymethoxy)-2-(4-(methoxymethoxy)phenyl)-4-oxo-4*H*-chromen-5-yl trifluoromethanesulfonate (**9c**) and propyne (1M in DMF, 2.4 mL, 2.4 mmol). Purification by FSC (isohexane/ethyl acetate 2:1). Yield: 8.63 g (28.4 mmol, 62%) beige crystals. ^1^H-NMR (400 MHz, CD_2_Cl_2_) *δ* 7.90–7.83 (m, 2H 2′-H, 6′-H), 7.17–7.13 (m, 2H, 3′-H, 5′-H), 7.11 (q, *J* = 2.5 Hz, 3H, 6-H, 8-H), 6.57 (s, 1H, 3-H), 5.27 (s, 2H, CH_2_), 5.25 (s, 2H, CH_2_), 3.50 (s, 3H, OCH_3_), 3.48 (s, 3H, OCH_3_), 2.16 (s, 3H, 3′-H). ^13^C-NMR (101 MHz, CD_2_Cl_2_) *δ* 176.5 (C-4), 161.4 (C-2), 159.9 (C-8a or C-4′), 159.9 (C-8a or C-4′), 158.3 (C-7), 127.6 (C-2′, C-6′), 124.8 (C-1′), 124.0 (C-5), 120.8 (C-6), 118.8 (C-4a), 116.4 (C-3′, 5′), 106.9 (C-3), 103.6 (C-8), 94.4 (CH_2_), 94.3 (CH_2_), 92.2 (C-2′), 78.6 (C-1′), 56.3 (OCH_3_), 56.1 (OCH_3_), 4.7 (C-3′). IR (ATR): *ṽ* (cm^−1^) 2910, 1636, 1595, 1508, 1375, 1344, 1241, 1142, 1075, 991, 950, 916, 836. HRMS (EI): calcd. for [C_22_H_21_O_6_]^+•^: 381.1333, found: 381.1338. M.p.: 123 °C.

*5-(Aminomethyl)-2-methylpyrano[2,3,4-ij]isoquinolin-8-ol (**11a**).* Prepared according to General Procedure B using 512 mg (1.37 mmol) crude *tert*-butyl (3-(7-(methoxymethoxy)-2-methyl-4-oxo-4*H*-chromen-5-yl)prop-2-yn-1-yl)carbamate (**10b**). Purification by FSC (dichloromethane/methanol 8:1 containing 0,1% triethylamine). Yield: 32 mg (0.14 mmol, 7% over 2 steps) yellow solid. ^1^H-NMR (500 MHz, (CD_3_)_2_SO) *δ* 10.68 (s, 1H, OH), 8.43 (s, 2H, NH_2_), 7.12 (s, 1H, 6-H), 6.66 (d, *J* = 2.3 Hz, 2H, 7-H, 9-H), 6.17 (s, 1H, 3-H), 3.96 (s, 2H, CH_2_), 2.23 (d, *J* = 0.9 Hz, 3H, CH_3_). ^13^C-NMR (126 MHz, CD_3_)_2_SO) *δ* 161.2 (C-8), 159.8 (C-2), 154.8 (C-9a), 150.9 (C-3a), 148.4 (C-5), 137.3 (C-6a), 113.1 (C-3a^1^), 112.1 (C6), 105.4 (C-3), 101.6 (C-7), 100.1 (C-9), 43.2 (CH_2_), 19.5 (CH_3_). IR (ATR): *ṽ* (cm^−1^) 3354, 2918, 1654, 1607, 1425, 1396, 1367, 1160, 1082, 833. HRMS (EI): calcd. for [C_13_H_12_N_2_O_2_]^+•^: 228.0893, found: 28.0892. M.p.: 120 °C. HPLC purity: 97% (210 nm), 98% (254 nm).

*2,5-Diphenylpyrano[2,3,4-ij]isoquinolin-8-ol (**11b**).* Prepared according to General procedure B using 191 mg (0.500 mmol) 7-(methoxymethoxy)-2-phenyl-5-(phenylethynyl)-4*H*-chromen-4-one (**10c**). Purification by FSC (dichloromethane/methanol 8:1). Yield: 38 mg (0.11 mmol, 23%) yellow solid. ^1^H-NMR (500 MHz, (CD_3_)_2_SO) *δ* 10.46 (s, 1H, OH), 8.16–8.08 (m, 2H, 2″-H, 6″-H), 8.02 (dd, *J* = 7.6, 2.2 Hz, 2H, 2′-H, 6′-H), 7.71 (s, 1H, 6-H), 7.59–7.37 (m, 6H, 3′-H, 4′-H, 5′-H, 3″-H, 4″-H, 5″-H), 7.11 (s, s1H, 3-H), 6.79–6.73 (m, 2H, 7-H, 9-H). ^13^C-NMR (126 MHz, (CD_3_)_2_SO) *δ* 160.9 (C-8), 156.7 (C-2), 154.4 (C-9a), 151.7 (C-5), 150.4 (C-3a), 139.1 (C-6a), 138.1 (C-1″), 131.7 (C-1′), 130.5 (C-4′, C4″), 128.9 (C-3′, C-5′), 128.5 (C-3″, C-5″), 126.5 (C-2″, C-6″), 125.3 (C-2′, C-6′), 112.7 (C-3a^1^), 111.3 (C-6), 104.7 (C-3), 102.1 (C-7), 100.0 (C-9). IR (ATR): *ṽ* (cm^−1^) 2168, 1655, 1608, 1468, 1299, 834, 764. HRMS (EI): calcd. for [C_15_H_14_N_2_O)^+•^: 337.1097, found: 337.1103. M.p.: 149 °C. HPLC purity: >99% (210 nm), >99% (254 nm).

*5-Methyl-2-phenylpyrano[2,3,4-ij]isoquinolin-8-ol (**11c**).* Prepared according to General Procedure B using 160 mg (0.500 mmol) 7-(methoxymethoxy)-2-phenyl-5-(prop-1-yn-1-yl)-4*H*-chromen-4-one (**10d**). Purification by FSC (dichloromethane/methanol 10:1). Yield: 44 mg (0.16 mmol, 32%) yellow solid. ^1^H-NMR (400 MHz, (CD_3_)_2_SO) *δ* 10.32 (s, 1H, OH), 8.00–7.92 (m, 2H, 2′-H, 6′-H), 7.58–7.47 (m, 3H, 3′-H, 4′-H, 5′-H), 6.98 (s, 1H, 3-H), 6.93 (d, *J* = 0.9 Hz, 1H, 6-H), 6.66 (d, *J* = 2.0 Hz, 1H, 7-H), 6.56 (d, *J* = 2.0 Hz, 1H, 9-H), 2.41–2.36 (m, 3H, CH_3_). ^13^C-NMR (101 MHz, (CD_3_)_2_SO) *δ* 160.7 (C-8), 156.2 (C-2), 154.5 (C-9a), 153.3 (C-5), 149.9 (C-3a), 138.0 (C-6a), 131.8 (C-4′), 130.3 (C-1′), 128.9 (C-3′, C-5′), 125.1 (C-2′, C-6′), 112.9 (C-6), 112.0 (C-3a^1^), 104.5 (C-7), 100.6 (C-3), 99.1 (C-9), 24.1 (CH_3_). IR (ATR): *ṽ* (cm^−1^) 2453, 1654, 1640, 1614, 1417, 1329, 1280, 1161, 896, 836, 768. HRMS (EI): calcd. for [C_18_H_13_NO_2_]^+•^: 275.0941, found: 275.0938. M.p.: 290 °C. HPLC purity: >99% (210 nm), >99% (254 nm).

*5-(Aminomethyl)-2-phenylpyrano[2,3,4-ij]isoquinolin-8-ol (**11d**).* Prepared according to General Procedure B using 218 mg (0.500 mmol) *tert*-butyl (3-(7-(methoxymethoxy)-4-oxo-2-phenyl-4*H*-chromen-5-yl)prop-2-yn-1-yl)carbamate (**10e**). Purification by FSC (dichloromethane/methanol 8:1 containing 0.1% triethylamine). Yield: 22 mg (14.9 mmol, 15%) yellow solid. ^1^H-NMR (400 MHz, CD_3_OD) *δ* 7.92 (dd, *J* = 6.7, 2.9 Hz, 2H, 2′-H, 6′-H), 7.53 (dd, *J* = 5.2, 2.0 Hz, 3H, 3′-H, 4′-H, 5′-H), 7.09 (s, 1H, 6-H), 6.88 (s, 1H, 3-H), 6.78 (d, *J* = 1.9 Hz, 1H, 9-H), 6.68 (d, *J* = 2.0 Hz, 1H, 7-H), 4.05 (s, 2H, CH_2_). ^13^C-NMR (101 MHz, CD_3_OD) *δ* 163.7 (C-8), 159.8 (C-2), 156.5 (C-9a), 152.9 (C-3a), 150.6 (C-5), 139.5 (C-6a), 133.4 (C-1′), 131.8 (C-4′), 130.0 (C-3′, C-5′), 126.4 (C-2′, C-6′), 114.5 (C-3a^1^ or C-6), 114.4 (C-3a^1^ or C-6), 104.6 (C-3), 103.4 (C-7), 101.7 (C-9), 45.6 (CH_2_). IR (ATR): *ṽ* (cm^−1^) 2729, 1644, 1598, 1574, 1451, 1391, 1290, 1208, 1171, 1146, 1005, 849, 770. HRMS (EI): calcd. for [C_18_H_14_N_2_O_2_]^+•^: 290.1050, found: 290.1052. M.p.: 280 °C. HPLC purity: 98% (210 nm), 96% (254 nm).

*5-(Hydroxymethyl)-2-phenylpyrano[2,3,4-ij]isoquinolin-8-ol (**11e**).* Prepared according to General Procedure B using 168 mg (0.500 mmol) 5-(3-hydroxyprop-1-yn-1-yl)-7-(methoxymethoxy)-2-phenyl-4*H*-chromen-4-one (**10f**). Purification by FSC (dichloromethane/methanol 8:1). Yield: 21 mg (0.072 mmol, 14%) yellow solid. ^1^H-NMR (500 MHz, CD_3_OD) *δ* 8.10–8.06 (m, 2H, 2′-H, 6′-H), 7.71–7.61 (m, 3H, 3′-H, 4′-H, 5′-H), 7.28 (t, *J* = 1.1 Hz, 1H, 6-H), 7.25 (s, 1H, 3-H), 7.15 (d, *J* = 2.0 Hz, 1H, 9-H), 7.01 (d, *J* = 2.0 Hz, 1H, 7-H), 4.70 (d, *J* = 1.0 Hz, 2H, CH_2_). ^13^C-NMR (126 MHz, CD_3_OD) *δ* 167.9 (C-8), 165.7 (C-2), 157.5 (C-9a), 150.0 (C-3a), 145.5 (C-5), 139.2 (C-6a), 134.0 (C-4′), 131.7 (C-1′), 130.5 (C-3′, C-5′), 127.7 (C-2′, C-6′), 114.1 (C-6), 112.0 (C-3a^1^), 106.8 (C-7), 103.3 (C-9), 97.1 (C-3), 61.3 (CH_2_). IR (ATR): *ṽ* (cm^−1^) 3322, 3063, 1652, 1634, 1608, 1598, 1434, 1377, 1353, 1162, 1137, 1098, 979, 848, 770. HRMS (EI): calcd. for [C_18_H_13_NO_3_]^+•^: 291.0890, found: 291.0892. M.p.: 290 °C. HPLC purity: 97% (210 nm), >99% (254 nm).

*5-(2-Hydroxyethyl)-2-phenylpyrano[2,3,4-ij]isoquinolin-8-ol (**11f**).* Prepared according to General Procedure B using 175 mg (0.500 mmol) 5-(4-hydroxybut-1-yn-1-yl)-7-(methoxymethoxy)-2-phenyl-4*H*-chromen-4-one (**10g**). Purification by FSC (dichloromethane/methanol 8:1). Yield: 18 mg (0.061 mmol, 12%) yellow solid. ^1^H-NMR (400 MHz, CD_3_OD) *δ* 7.75 (dd, *J* = 7.4, 2.3 Hz, 2H, 2′-H, 6′-H), 7.47–7.34 (m, 3H, 3′-H, 4′-H, 5′-H), 6.80 (s, 1H, 6-H), 6.69 (s, 1H, 3-H), 6.58 (d, *J* = 1.8 Hz, 1H, 9-H), 6.49 (d, *J* = 1.9 Hz, 1H, 7-H), 3.81 (t, *J* = 6.4 Hz, 2H, 2″-H), 2.76 (t, *J* = 6.5 Hz, 2H, 1″-H). ^13^C-NMR (101 MHz, CD_3_OD) *δ* 165.3 (C-8), 161.1 (C-2), 156.6 (C-9a), 151.0 (C-5), 150.7 (C-3a), 139.5 (C-6a), 132.7 (C-1′), 132.3 (C-4′), 130.1 (C-3′, C-5′), 126.7 (C-2′, C-6′), 115.7 (C-6), 112.8 (C-3a^1^), 104.0 (C-7), 101.6 (C-9), 101.5 (C-3), 62.0 (C-2″), 40.2 (C-1″). IR (ATR): *ṽ* (cm^−1^) 3387, 3087, 2930, 1653, 1636, 1608, 1597, 1439, 1373, 1278, 1163, 1075, 1027, 853, 774. HRMS (EI): calcd. for [C_19_H_15_NO_3_]^+•^: 305.1046, found: 305.1046. M.p.: 280 °C. HPLC purity: 98% (210 nm), >99% (254 nm). 

*2-(4-Hydroxyphenyl)-5-methylpyrano[2,3,4-ij]isoquinolin-8-ol (**11g**).* Prepared according to General Procedure B using 190 mg (0.500 mmol) 7-(methoxymethoxy)-2-(4-(methoxymethoxy)phenyl)-5-(prop-1-yn-1-yl)-4*H*-chromen-4-one (**10h**). Purification by FSC (dichloromethane/methanol 8:1). Yield: 27 mg (0.235 mmol, 19%) yellow solid. ^1^H-NMR (500 MHz, (CD_3_)_2_SO) *δ* 11.58 (s, 1H, 8-OH), 10.62 (s, 1H, 4′-OH), 7.92–7.85 (m, 2H, 2′-H, 6′-H), 7.13 (d, *J* = 1.2 Hz, 1H, 6-H), 7.07 (d, *J* = 2.0 Hz, 1H, 9-H), 7.06 (s, 1H, 3-H), 7.03–6.98 (m, 2H, 3′-H, 5′-H), 6.89 (d, *J* = 2.0 Hz, 1H, 7-H), 2.45 (s, 3H, CH_3_). ^13^C-NMR (126 MHz, (CD_3_)_2_SO) *δ* 165.4 (C-8), 163.0 (C-2), 161.9 (C-4′), 155.3 (C-9a), 147.9 (C-3a), 141.1 (C-5), 137.4 (C-6a), 128.6 (C-2′, C-6′), 120.4 (C-1′), 116.3 (C-3′, C-5′), 113.7 (C6), 109.2 (C-3a^1^), 104.3 (C-7), 101.3 (C-9), 93.6 (C-3), 18.7 (CH_3_). IR (ATR): *ṽ* (cm^−1^) 3092, 2932, 1650, 1636, 1600, 1581, 1556, 1500, 1429, 1368, 1294, 1220, 1168, 847, 838. HRMS (EI): calcd. for [C_17_H_15_N_3_O_2_]^+•^: 291.0890, found: 291.0895. M.p.: 280 °C. HPLC purity: >99% (210 nm), >99% (254 nm).

*1-(2-Isocyano-2-tosylpropyl)-3,5-dimethoxybenzene (**14**).* To a solution of 3,5-dimethoxybenzylbromide (**12**; 4.35 g, 18.8 mmol) in 100 mL, dichloromethane was added tetrabutylammonium bromide (1.39 g, 3.76 mmol) and tosylmethylisocyanide (**13**; 3.67 g, 18.8 mmol) and the mixture was cooled to 0 °C. Then, 100 mL sodium hydroxide solution (40% in water) was added, and the mixture was stirred without cooling for 1 h. Then, 7.03 mL (16.0 g, 113 mmol) iodomethane was added and the mixture was stirred vigorously for 18 h. Then, 150 mL water was added, and the organic layer was separated. The aqueous layer was extracted with 3 × 50 mL dichloromethane, and the combined organic layers were washed with 2 × 25 mL brine, dried over sodium sulphate and evaporated. The residue was purified by FCC (isohexane/ethyl acetate 5:1). Yield: 2.45 g (6.82 mmol, 36%) colourless solid. ^1^H-NMR (400 MHz, CD_2_Cl_2_) *δ* 7.91 (d, *J* = 8.4 Hz, 2H, 2″-H, 6″-H), 7.52–7.45 (m, 2H, 3″-H, 5″-H), 6.44–6.42 (m, 1H, 4-H), 6.41 (d, *J* = 2.2 Hz, 2H, 2-H, 6-H), 3.77 (s, 6H, OCH_3_), 3.16 (q, *J* = 13.4 Hz, 2H, 1′-H), 2.50 (s, 4H, 4″-CH_3_), 1.53 (s, 3H, 3′-H). ^13^C-NMR (101 MHz, CD_2_Cl_2_) *δ* 165.5 (NC), 161.3 (C-3, C-5), 147.3 (C-4″), 134.7 (C-1), 131.7 (C-3″, C-5″), 130.4 (C-2″, C-6″), 129.8 (C-1″), 109.2 (C-2, C-6), 100.2 (C-4), 79.2 (C-2′), 55.7 (OCH_3_), 39.9 (C-1′), 22.0 (4″-CH_3_), 20.8 (C-3′). IR (ATR): *ṽ* (cm^−1^) 2940, 2128, 1697, 1596, 1456, 1429, 1322, 1295, 1208, 1154, 1071, 1053, 832, 816, 706. HRMS (EI): calcd. for [C_19_H_21_NO_4_S]^+•^: 359.1186, found: 359.1189. M.p.: 108 °C.

*1-Iodo-6,8-dimethoxy-3-methylisoquinoline (**15**).* A solution of 1-(2-isocyano-2-tosylpropyl)-3,5-dimethoxybenzene (**14**; 2.00 g, 5.57 mmol) and *N*-iodosuccinimide (2.58 g, 11.1 mmol) in 100 mL dichlormethane in a Schlenk flask was stirred under nitrogen for 22 h at ambient temperature, then cooled to 0 °C. A LiHMDS solution (1M in THF, 22.3 mL, 22.3 mmol) was added slowly and the mixture stirred for another 30 min at 0 °C and 7 h at ambient temperature. Then, 75 mL water was added carefully, the organic layer was separated, and the aqueous layer was extracted with 2 × 25 mL dichloromethane. The combined organic layers were washed with 2 × 25 mL brine, dried over sodium sulphate and evaporated. The residue was purified by FCC (isohexane/ethyl acetate 5:1). Yield: 1.21 g (3.69 mmol, 66%) pale yellow solid. ^1^H-NMR (500 MHz, CDCl_3_) *δ* 7.17 (s, 1H, 4-H), 6.51 (d, *J* = 2.4 Hz, 1H, 5-H or 7-H), 6.50 (d, *J* = 2.4 Hz, 1H, 5-H or 7-H), 3.93 (s, 3H, OCH_3_), 3.89 (s, 3H, OCH_3_), 2.55 (d, *J* = 0.8 Hz, 3H, CH_3_). ^13^C-NMR (126 MHz, CDCl_3_) *δ* 161.5 (C-6), 156.1 (C-8), 153.1 (C3), 141.1 (C4a), 118.4 (C4), 117.0 (C-8a), 112.4 (C1), 99.7 (C-7), 97.4 (C-5), 55.6 (OCH_3_), 54.9 (OCH_3_), 23.7 (CH_3_). IR (ATR): *ṽ* (cm^−1^) 2920, 1619, 1557, 1391, 1378, 1275, 1210, 1148, 1057, 862, 932, 817. HRMS (EI): calcd. for [C_12_H_12_INO_2_]^+•^: 328.9907, found: 328.9916. M.p.: 110 °C.

*4-(2-(2-Ethoxyethoxy)ethoxy)-phenylacetylene (**16c**).* To a solution of ((4-(2-(2-ethoxyethoxy)ethoxy)phenyl)ethynyl)trimethylsilane [29] (505 mg, 1.65 mmol) in 20 mL methanol was added potassium carbonate (300 mg, 1.82 mmol), the flask was closed, and the mixture stirred for 16 h at ambient temperature. Then, 20 mL water was added, followed by extraction with 3 × 25 mL dichloromethane. The combined organic layers were washed with 2 × 25 mL brine, dried over sodium sulphate and evaporated. The residue was purified by FCC (isohexane/ethyl acetate 5:2). Yield: 309 mg (1.32 mmol, 80%) pale yellow oil. ^1^H-NMR (400 MHz, CD_2_Cl_2_]: *δ* 7.46–7.37 (m, 2H, 3-H, 5-H), 6.91–6.82 (m, 2H, 2-H, 6-H), 4.15–4.08 (m, 2H, 2′-H), 3.84–3.77 (m, 2H, 3′-H), 3.69–3.62 (m, 2H, 5′-H or 6′-H), 3.60–3.53 (m, 2H, 5′-H or 6′-H), 3.49 (qd, *J* = 7.0, 0.7 Hz, 2H, 8′-H), 3.04 (s, 1H, 2″-H), 1.17 (td, *J* = 7.0, 0.8 Hz, 3H, 9′-H). ^13^C-NMR (126 MHz, CD_2_Cl_2_) *δ* 159.9 (C-1), 134.1 (C3, C-5), 115.1 (C-2, C-6), 114.8 (C-4), 84.0 (C-1″), 76.2 (C-2″), 71.4 (C-5′ or C-6′), 70.4 (C-5′ or C-6′), 70.1 (C-3′), 68.2 (C-2′), 67.0 (C-8′), 15.6 (C-9′). IR (ATR): *ṽ* (cm^−1^) 3284, 2870, 1605, 1506, 1288, 1246, 1101, 1058, 832. HRMS (EI): calcd. for [C_14_H_18_O_3_]^+•^: 234.1250, found: 234.1250. HPLC purity: >99% (210 nm), >99% (254 nm).

*6,8-Dimethoxy-3-methyl-1-(prop-1-yn-1-yl)isoquinoline (**17a**).* Prepared according to General Procedure C using 1.22 mL (1.22 mmol) propyne (1 M in THF). Purification by FSC (isohexane/ethyl acetate 1:1). Yield: 136 mg (0.564 mmol, 93%) colourless solid. ^1^H-NMR (400 MHz, CD_2_Cl_2_) *δ* 7.23 (s, 1H, 4-H), 6.57 (d, *J* = 2.3 Hz, 1H, 7-H), 6.47 (d, *J* = 2.3 Hz, 1H, 5-H), 3.95 (s, 3H, OCH_3_), 3.89 (s, 3H, OCH_3_), 2.53 (d, *J* = 0.7 Hz, 3H, CH_3_), 2.16 (s, 3H, 3′-H). ^13^C-NMR (101 MHz, CD_2_Cl_2_) *δ* 162.0 (C-6), 158.6 (C-8), 152.9 (C-3), 141.3 (C-4a), 140.3 (C-1), 118.0 (C-4), 116.6 (C-8a), 99.4 (C-5), 97.1 (C-7), 89.3 (C-2′), 82.1 (C-1′), 56.4 (OCH_3_), 56.0 (OCH_3_), 24.3 (CH_3_), 5.0 (C-3′). IR (ATR): *ṽ* (cm^−1^) 2914, 1612, 1558, 1459, 1396, 1363, 1258, 1204, 1160, 1119, 1049, 911, 936, 858, 825, 790. HRMS (EI): calcd. for [C_15_H_15_NO_2_]^+•^: 241.1097, found: 241.1093. M.p.: 125–126 °C.

*1-(Hex-1-yn-1-yl)-6,8-dimethoxy-3-methylisoquinoline (**17b**).* Prepared according to General Procedure C using 144 µL (102 mg, 1.22 mmol) hex-1-yne (**16b**). Purification by FSC (isohexane/ethyl acetate 1:1). Yield: 158 mg (0.558 mmol, 92%) yellow solid. ^1^H-NMR (400 MHz, CD_2_Cl_2_) *δ* 7.24 (s, 1H, 4-H), 6.57 (d, *J* = 2.3 Hz, 1H, 5-H), 6.47 (d, *J* = 2.2 Hz, 1H, 7-H), 3.94 (s, 3H, OCH_3_), 3.89 (s, 3H, OCH_3_), 2.58–2.50 (m, 5H, 3′H, CH_3_), 1.77–1.60 (m, 2H, 4′-H), 1.61–1.47 (m, 2H, 5′-H), 0.98 (t, *J* = 7.3 Hz, 3H, 6′-H). ^13^C-NMR (101 MHz, CD_2_Cl_2_) *δ* 162.1 (C-6), 158.6 (C-8), 152.7 (C-3), 141.3 (C-1), 140.4 (C-4a), 118.1 (C-4), 116.5 (C-8a), 99.4 (C-5), 97.1 (C-7), 94.3 (C-2′), 82.9 (C-1′), 56.1 (OCH_3_), 56.0 (OCH_3_) 31.4 (C-4′), 24.2 (CH_3_), 22.7 (C-5′), 20.1 (C-3′), 14.0 (C-6′). IR (ATR): *ṽ* (cm^−1^) 1957, 2932, 2871, 1617, 1561, 1452, 1397, 1365, 1201, 1161, 1119, 826, 723. HRMS (EI): calcd. for [C_18_H_21_NO_2_]^+•^: 283.1567, found: 283.1568. M.p.: 137 °C.

*1-((4-(2-(2-Ethoxyethoxy)ethoxy)phenyl)ethynyl)-6,8-dimethoxy-3-methylisoquinoline (**17c**).* Prepared according to General Procedure C using 285 mg (1.22 mmol) 4-(2-(2-ethoxyethoxy)ethoxy)-phenylacetylene (**16c**). Purification by FSC (isohexane/ethyl acetate 1:1). Yield: 229 mg (0.526 mmol, 87%) yellow solid. ^1^H-NMR (400 MHz, CD_2_Cl_2_) *δ* 7.62–7.54 (m, 2H, 2″-H, 6″-H), 7.31–7.25 (m, 1H, 4-H), 7.00–6.91 (m, 2H, 3″-H, 5″-H), 6.61 (d, *J* = 2.3 Hz, 1H, 5-H), 6.52 (d, *J* = 2.2 Hz, 1H, 7-H), 4.20–4.13 (m, 2H, 2‴-H), 4.02 (s, 3H, 8-OCH_3_), 3.91 (s, 3H, 6-OCH_3_), 3.87–3.80 (m, 2H, 3‴-H), 3.71–3.64 (m, 2H, 5‴-H or 6‴-H), 3.61–3.54 (m, 2H, 5‴-H or 6‴-H), 3.55–3.45 (dq, *J* = 7.0, 0.7 Hz, 2H, 8‴-H), 2.58 (d, *J* = 0.7 Hz, 3H, CH_3_), 1.23–1.13 (td, *J* = 7.0, 0.8 Hz, 3H, 9‴-H). ^13^C-NMR (101 MHz, CD_2_Cl_2_) *δ* 162.1 (C-6), 159.9 (C-4″), 158.5 (C-8), 153.1 (C-3), 141.3 (C-4a), 140.1 (C-1), 134.1 (C-2″, C-6″), 118.2 (C-4), 116.6 (C-8a), 116.1 (C-1″), 115.3 (C-3″, C-5″), 99.6 (C7), 97.2 (C-5), 91.8 (C-2′), 91.0 (C-1′), 71.5 (C-5‴ or C-6‴), 70.4 (C-5‴ or C-6‴), 70.1 (C-3‴), 68.2 (C-2‴), 67.1 (C-8‴), 56.4 (8-OCH_3_), 56.0 (6-OCH_3_), 24.3 (CH_3_), 15.6 (C-9‴). IR (ATR): *ṽ* (cm^−1^) 2868, 2199, 1621, 1604, 1558, 1507, 1454, 1398, 1367, 1245, 1208, 1160, 1101, 1062, 1036, 856, 837, 822, 794. HRMS (EI): calcd. for [C_26_H_29_NO_5_]^+•^: 435.2040, found: 435.2039. M.p.: 85 °C.

*Methyl 4-((6,8-dimethoxy-3-methylisoquinolin-1-yl)ethynyl)benzoate (**17d**).* Prepared according to General Procedure C using 329 mg (1.00 mmol) methyl 4-ethynylbenzoate (**16d**). Purification by FSC (isohexane/ethyl acetate 1:1). Yield: 258 mg (0.714 mmol, 71%) orange solid. ^1^H-NMR (400 MHz, CD_2_Cl_2_) δ 8.07–8.02 (m, 2H, 3″-H, 5″-H), 7.73–7.68 (m, 2H, 2″-H, 6″-H), 7.32 (s, 1H, 4-H), 6.61 (d, *J* = 2.3 Hz, 1H, 5-H), 6.53 (d, *J* = 2.2 Hz, 1H, 7-H), 4.01 (s, 3H, OCH_3_), 3.91 (s, 3H, COOCH_3_), 3.91 (s, 3H, OCH_3_), 2.60 (d, *J* = 0.7 Hz, 3H, CH_3_). ^13^C-NMR (101 MHz, CD_2_Cl_2_) δ 166.9 (COO), 162.2 (C-6), 158.3 (C-8), 153.2 (C-3), 141.3 (C-1), 139.3 (C-4a), 132.4 (C-2″, C6″), 130.6 (C-1″), 130.0 (C-3″, C-5″), 128.5 (C-4″), 119.0 (C-4), 116.9 (C-8a), 99.8 (C-7), 97.2 (C-5), 94.5 (C-1′), 90.4 (C-2′), 56.4 (OCH_3_), 56.1 (OCH_3_), 52.7 (COOCH_3_), 24.3 (CH_3_). IR (ATR): *ṽ* (cm^−1^) = 2939, 1722, 1558, 1399, 1271, 1204, 1161, 1115, 1105, 879, 856, 724, 692. HRMS (EI): (calcd. for [C_22_H_19_NO_4_]^+•^: 361.1313, found: 361.1314. M.p.: 167–169 °C.

*2-Butyl-5-methylpyrano[2,3,4-ij]isoquinolin-8-ol (**18a**).* To a solution of 1-(hex-1-yn-1-yl)-6,8-dimethoxy-3-methylisoquinoline (**17b**) (198 mg, 0.700 mmol) in 7 mL dichloromethane in a Schlenk flask under nitrogen was added dropwise boron tribromide (1.82 mL, 1.58 g, 6.31 mmol) and then the mixture refluxed for 6 h. After cooling 6 mL methanol and 5 mL 10% aqueous ammonia were added and the mixture was stirred vigorously for 15 min. The mixture was evaporated to dryness and the residue was purified by FSC (dichloromethane/methanol 10:1). Yield: 113 mg (0.443 mmol, 63%) yellow solid. ^1^H-NMR (400 MHz, (CD_3_)_2_SO) *δ* 10.25 (s, 1H, OH), 6.85 (d, *J* = 0.9 Hz, 1H, 6-H), 6.50 (d, *J* = 2.0 Hz, 1H, 9-H), 6.47 (d, *J* = 2.0 Hz, 1H, 7-H), 6.10 (s, 1H, 3-H), 2.46 (t, *J* = 7.5 Hz, 2H, 1′-H), 2.33 (d, *J* = 0.8 Hz, 3H, CH_3_), 1.61 (p, *J* = 7.5 Hz, 2H, 2′-H), 1.44–1.33 (m, 2H, 3′-H), 0.93 (t, *J* = 7.3 Hz, 3H, 4′-H). ^13^C-NMR (101 MHz, (CD_3_)_2_SO) *δ* 162.7 (C-2), 161.1 (C-9a), 155.4 (C-8), 153.2 (C-5), 150.5 (C-3a), 138.5 (C-6a), 112.9 (C-6), 111.9 (C-3a^1^), 105.5 (C-3), 101.0 (C-7), 99.2 (C-9), 32.9 (C-1′), 28.8 (C-2′), 24.4 (CH_3_), 22.0 (C-3′), 14.1 (C-4′). IR (ATR): *ṽ* (cm^−1^) 2920, 2191, 1648, 1614, 1428, 1394, 1375, 1320, 1165, 834, 722. HRMS (EI): calcd. for [C_14_H_18_O_3_]^+•^: 256.1332, found: 255.1335. M.p.: 230 °C.

*2-(4-(8-Methoxy-5-methylpyrano[2,3,4-ij]isoquinolin-2-yl)phenoxy)ethan-1-ol (**18b**).* To a solution of 1-((4-(2-(2-ethoxyethoxy)ethoxy)phenyl)ethynyl)-6,8-dimethoxy-3-methylisoquinoline (**17c**; 135 mg, 0.311 mmol) in 2 mL dichloromethane in a Schlenk flask under nitrogen was added dropwise boron tribromide (65.9 µL, 0.684 mmol) and the mixture was stirred at ambient temperature for 16 h. Then, 1.5 mL methanol and 3 mL 10% aqueous ammonia were added, and the mixture was stirred vigorously for 15 min. The mixture was evaporated to dryness and the residue was purified by FSC (dichloromethane/methanol 10:1). Yield: 13 mg (0.037 mmol, 12%) yellow solid. ^1^H-NMR (400 MHz, CD_2_Cl_2_) *δ* 7.83–7.75 (m, 2H, 2′-H, 6′-H), 7.05–6.99 (m, 2H, 3′-H, 5′-H), 6.90 (d, *J* = 0.9 Hz, 1H, 6-H), 6.71 (s, 1H, 3-H), 6.66 (d, *J* = 2.1 Hz, 1H, 7-H or 9-H), 6.58 (d, *J* = 2.2 Hz, 1H, 7-H or 9-H), 4.14 (dd, *J* = 5.1, 4,0 Hz, 2H, 1″-H or 2″-H), 3.96 (dd, *J* = 5.2, 3.9 Hz, 2H, 1″-H or 2″-H), 3.89 (s, 3H, OCH_3_), 2.46 (d, *J* = 0.8 Hz, 3H, CH_3_). ^13^C-NMR (101 MHz, CD_2_Cl_2_) *δ* 163.1 (C-8), 161.0 (C-4′), 157.8 (C-2), 155,7 (C-3a), 155.7 (C-5), 151.4 (C-9a), 139.1 (C-6a), 127.4 (C-2′, C-6′), 125.8 (C-1′), 115.3 (C-3′, C-5′), 113.8 (C-6), 113.7 (C-3a^1^), 103.8 (C-3), 99.1 (C-7 or C-9), 98.5 (C-7 or C-9), 70.2 (C-2″ or C-3″), 61.8 (C-2″ or C-3″), 56.2 (OCH_3_), 24.7 (CH_3_). IR (ATR): *ṽ* (cm^−1^) 3172, 2920, 1648, 1607, 1573, 1512, 1496, 1450, 1413, 1363, 1258, 1165, 1053, 1034, 920, 850, 812, 793. HRMS (EI): calcd. for [C_14_H_18_O_3_]^+•^: 349.1309, found: 349.1305. M.p.: 216 °C. HPLC purity: >99% (210 nm), >99% (254 nm).

*6,8-Bis(methoxymethoxy)-3-methyl-1H-isochromen-1-one (**20**).* 6,8-Dihydroxy-3-methyl-1*H*-isochromen-1-one (**19**; 500 mg, 2.60 mmol) and cesium carbonate (2.54 g, 7.80 mmol) in 20 mL DMF were stirred for 30 min at ambient temperature. Then, chloromethyl methylether (494 µL, 523 mg, 6.50 mmol) was added, the flask was closed and the mixture stirred for 16 h. After addition of 50 mL water and 50 mL ethyl acetate the organic layer was separated and the aqueous layer extracted with 2 × 30 mL ethyl acetate. The combined organic layers were washed with 2 × 25 mL satd. lithium chloride solution, dried over sodium sulphate and evaporated. The residue was purified by FCC (isohexane/ethyl acetate 2:1). Yield: 725 mg (2.59 mmol, >99%) colourless solid. ^1^H-NMR (400 MHz, (CD_3_)_2_SO) *δ* 6.74 (d, *J* = 2.2 Hz, 1H, 5-H or 7-H), 6.70 (d, *J* = 2.3 Hz, 1H, 5-H or 7-H), 6.37 (d, *J* = 1.2 Hz, 1H, 4-H), 5.30 (d, *J* = 2.6 Hz, 4H, OCH_2_O), 3.42 (s, 3H, OCH_3_), 3.40 (s, 3H, OCH_3_), 2.16 (d, *J* = 1.0 Hz, 3H, CH_3_). ^13^C-NMR (101 MHz, (CD_3_)_2_SO) *δ* 162.3 (C-6), 160.1 (C-8), 157.7 (C-1), 155.1 (C-3), 141.7 (C-4a), 103.4 (C-7), 103.3 (C-8a), 103.2 (C-4), 103.0 (C-5), 94.7 (OCH_2_O), 93.8 (OCH_2_O), 56.1 (OCH_3_), 56.1 (OCH_3_), 19.0 (CH_3_). IR (ATR): *ṽ* (cm^−1^) 2921, 1721, 1672, 1599, 1570, 1462, 1357, 1145, 1032, 1015, 922, 910, 848, 831. HRMS (EI): (M)^•+^ not found. M.p.: 78 °C).

*6,8-Bis(methoxymethoxy)-3-methylisoquinolin-1(2H)-one (**21**).* To a solution of 6,8-bis(methoxymethoxy)-3-methyl-1*H*-isochromen-1-one (**20**; 561 mg, 2.00 mmol) in 10 mL DMF was added 10 mL aqueous ammonia solution (25%) and the mixture was heated with stirring at 80 °C for 16 h. After cooling, 30 mL water was added, followed by extraction with 3 × 25 mL ethyl acetate. The combined organic layers were washed with 2 × 30 mL satd. lithium chloride solution, dried over sodium sulphate and evaporated. The residue was purified by FCC (dichloromethane/methanol 30:1). Yield: 510 mg (1.83 mmol, 91%) colourless solid. ^1^H-NMR (400 MHz, (CD_3_)_2_SO) *δ* 10.78 (s, 1H, NH), 6.69 (d, *J* = 2.3 Hz, 1H, 5-H or 7-H), 6.60 (d, *J* = 2.3 Hz, 1H, 5-H or 7-H), 6.11 (d, *J* = 1.3 Hz, 1H, 4-H), 5.25 (s, 2H, OCH_2_O), 5.21 (s, 2H, OCH_2_O), 3.43 (s, 3H, OCH_3_), 3.39 (s, 3H, OCH_3_), 2.11 (s, 3H, CH_3_). ^13^C-NMR (101 MHz, (CD_3_)_2_SO) *δ* 160.4 (C-1), 159.7 (C-8), 159.1 (C-6), 142.7 (C-4a), 139.5 (C-3), 109.9 (C-8a), 103.3 (C-7), 103.1 (C-5), 102.5 (C-4), 95.4 (OCH_2_O), 93.7 (OCH_2_O), 55.9 (OCH_3_), 55.9 (OCH_3_), 18.4 (CH_3_). IR (ATR): *ṽ* (cm^−1^) 2904, 1639, 1603, 1556, 1352, 1144, 1109, 1057, 1033, 964, 898, 866, 756. HRMS (EI): calcd. for [C_14_H_17_NO_5_]^+•^: 279.1101, found: 279.1100. M.p.: 156 °C).

*6,8-Bis(methoxymethoxy)-3-methylisoquinolin-1-yl trifluoromethanesulfonate (**22**).* An ice-cooled solution of 6,8-bis(methoxymethoxy)-3-methylisoquinolin-1(2*H*)-one (**21**; 475 mg, 1.70 mmol) 10 mL DMF was treated with 81.6 mg (2.04 mmol) sodium hydride (60% dispersion in paraffin) and stirred for 15 min. Then, *N*-phenyl-bis-(trifluoromethanesulfonimide) (911 mg, 2.55 mmol) was added, and the mixture was stirred at ambient temperature for 16 h. Then, 50 mL diethyl ether was added, the solution was washed with 25 mL water and 25 mL brine dried over sodium sulphate and evaporated. The residue was purified by FCC (isohexane/ethyl acetate 2:1). Yield: 504 mg (1.23 mmol, 72%) colourless solid. ^1^H-NMR (400 MHz, CDCl_3_) *δ* 7.33 (d, *J* = 1.0 Hz, 1H, 4-H), 6.96 (d, *J* = 2.1 Hz, 1H, 5-H or 7-H), 6.94 (d, *J* = 2.1 Hz, 1H, 5-H or 7-H), 5.39 (s, 2H, CH_2_), 5.28 (s, 2H, CH_2_), 3.56 (s, 3H, OCH_3_), 3.51 (s, 3H, OCH_3_), 2.54 (d, *J* = 0.9 Hz, 3H, CH_3_). ^13^C-NMR (101 MHz, CDCl_3_) *δ* 160.1 (C-6), 154.4 (C-8), 150.4 (C-1), 150.3 (C-3), 144.0 (C-4a), 119.9 (C-4), 118.8 (q, *J* = 320.3 Hz, CF_3_), 107.8 (C-8a), 104.0 (C-5), 101.3 (C-7), 94.7 (CH_2_), 94.5 (CH_2_), 56.9 (OCH_3_), 56.6 (OCH_3_), 23.4 (CH_3_). IR (ATR): *ṽ* (cm^−1^) 3352, 2228, 1593, 1507, 1264, 1201, 1170, 1028, 903, 878, 801. HRMS **(EI):** calcd. for [C_15_H_16_F_3_NO_7_S]^+•^: 411.0594, found: 411.0596. M.p.: 85–86 °C).

*Methyl 4-((6,8-bis(methoxymethoxy)-3-methylisoquinolin-1-yl)ethynyl)benzoate (**23**).* In a Schlenk flask 6,8-bis(methoxymethoxy)-3-methylisoquinolin-1-yl trifluoromethanesulfonate (**22**) (411 mg, 1.00 mmol), bis(triphenylphosphane)palladium(II) dichloride (35.1 mg, 0.0500 mmol) and copper(I)iodide (19 mg, 0.10 mmol) were suspended in 7 mL THF and set under nitrogen. The flask was closed with a rubber septum and methyl 4-ethynylbenzoate (0.294 mL, 327 mg, 2 mmol) and 7 mL diisopropylamine were added with syringes. The mixture was stirred for 18 h, then evaporated to dryness. The residue was purified by FCC (isohexane/ethyl acetate 2:1). Yield: 84 mg (0.25 mmol, 84%) yellow solid. ^1^H-NMR (400 MHz, CDCl_3_) *δ* 8.09–8.01 (m, 2H, 3″-H, 5″-H), 7.75–7.67 (m, 2H, 2″-H, 6″-H), 7.32 (s, 1H, 4-H), 6.90 (d, *J* = 2.2 Hz, 1H, 5-H), 6.83 (d, *J* = 2.2 Hz, 1H, 7-H), 5.38 (s, 2H, CH_2_), 5.28 (d, *J* = 0.9 Hz, 2H, CH_2_), 3.93 (d, *J* = 0.8 Hz, 3H, COOCH_3_), 3.52 (s, 3H, OCH_3_), 3.51 (s, 3H, OCH_3_), 2.65 (d, *J* = 0.8 Hz, 3H, CH_3_). ^13^C-NMR (101 MHz, CDCl_3_) *δ* 166.7 (COO), 159.0 (C-6), 155.2 (C-8), 152.6 (C-3), 140.5 (C-4a), 139.3 (C-1), 132.0 (C-2″, C6″), 130.0 (C-1″), 129.6 (C-3″, C-5″), 128.2 (C-4″), 119.0 (C-4), 117.1 (C-8a), 103.5 (C-7), 101.5 (C-5), 95.1 (CH_2_), 94.5 (CH_2_), 94.0 (C-1′), 90.5 (C-2′), 56.8 (OCH_3_), 56.6 (OCH_3_), 52.4 (COOCH_3_), 24.3 (CH_3_). IR (ATR): *ṽ* (cm^−1^) 3435, 2923, 1721, 1619, 1561, 1278, 1153, 1018, 927, 767. HRMS (EI): calcd. for [C_24_H_23_NO_6_]^+•^: 421.1520, found: 421.1519. M.p.: 120 °C.

*Methyl 4-(8-hydroxy-5-methylpyrano[2,3,4-ij]isoquinolin-2-yl)benzoate (**18c**).* To a solution of methyl 4-((6,8-bis(methoxymethoxy)-3-methylisochinolin-1-yl)ethynyl)benzoat (**23**; 126 mg, 0.300 mmol) in 10 mL methanol was added slowly and under stirring 5 mL hydrochloric acid (10%). Then, the flask was closed and the mixture heated with stirring to 40 °C for 36 h, then evaporated to dryness. The residue was purified by FCC (dichloromethane/methanol 20:1). Yield: 84 mg (0.25 mmol, 84%) yellow solid. ^1^H-NMR (500 MHz, (CD_3_)_2_SO) *δ* 10.40 (s, 1H, OH), 8.18–8.02 (m, 4H, 2′-H, 3′-H, 5′-H, 6′-H), 7.15 (s, 1H, 6-H), 6.98 (s, 1H, 9-H), 6.69 (s, 1H, 7-H), 6.59 (s, 1H, 3-H), 3.90 (s, 3H, COOCH_3_), 2.40 (s, 3H, CH_3_). ^13^C-NMR (126 MHz, (CD_3_)_2_SO) *δ* 166.1 (COO), 161.3 (C-2), 155.6 (C-8), 154.8 (C-9a), 149.9 (C-3a), 138.5 (C-5), 136.5 (C-1′), 135.1 (C-6a), 131.2 (C-4′), 130.1 (C-3′, C-5′), 125.9 (C-2′, C-6′), 113.9 (C-6), 112.7 (C-C-3a^1^), 106.8 (C-3), 101.3 (C-7), 99.8 (C-9), 52.9 (COOCH_3_), 24.4 (CH_3_). IR (ATR): *ṽ* (cm^−1^) 3435, 1733, 1613, 1423, 1280, 1171, 1108, 834, 768. HRMS (EI): calcd. for [C_20_H_15_NO_4_]^+•^: 333.0996, found: 333.0988. M.p.: 248 °C (decomp.). HPLC purity: >99% (210 nm), >99% (254 nm).

*5-Methyl-2-phenyl-8-(prop-2-in-1-yloxy)pyrano[2,3,4-ij]isoquinoline (**24**).* To a solution of 5-methyl-2-phenylpyrano[2,3,4-*ij*]isoquinolin-8-ol (**11c**) (12.4 mg, 45.0 µmol) in 1 mL DMF was added cesium carbonate (29.3 mg, 90.0 µmol) and the mixture was stirred at ambient temperature for 15 min. Then, propargyl bromide (4.8 µL, 6.4 mg, 54 µmol) was added. The mixture was stirred at ambient temperature for 16 h, then evaporated to dryness. The residue was purified by FCC (dichloromethane/methanol 10:1). Yield: 13 mg (42 µmol, 92%) yellow solid. ^1^H-NMR (500 MHz, CD_2_Cl_2_) *δ* 7.91–7.82 (m, 2H, 2′-H, 6′-H), 7.48 (qt, *J* = 5.1, 2.2 Hz, 3H, 3′-H, 4′-H, 5′-H), 6.93 (s, 1H, 6-H), 6.81 (s, 1H, 3-H), 6.72 (d, *J* = 2.1 Hz, 1H, 9-H), 6.66 (d, *J* = 2.1 Hz, 1H, 7-H), 4.80 (d, *J* = 2.3 Hz, 2H, 1″-H), 2.64 (t, *J* = 2.4 Hz, 1H, 3″-H), 2.47 (s, 3H, CH_3_). ^13^C-NMR (126 MHz, CD_2_Cl_2_) *δ* 160.8 (C-8), 157.8 (C-2), 155.8 (C-9a), 155.1 (C-5), 151.2 (C-3a), 138.9 (C-6a), 133.0 (C-1′), 130.7 (C-4′), 129.3 (C-2′, C-5′), 125.8 (C-2′, C-6′), 114.3 (C-3a^1^), 114.2 (C-6), 105.6 (C-3), 99.7 (C-7), 99.4 (C-9), 78.6 (C-2″), 76.3 (C-3″), 56.6 (C-2″), 24.9 (CH_3_). IR (ATR): *ṽ* (cm^−1^) = 3123, 2104, 1646, 1612, 1573, 1418, 1361, 1268, 1159, 1105, 841, 759. HRMS (EI): calcd. for [C_14_H_18_O_3_]^+•^: 314.1176, found: 314.1176. M.p.: 201 °C.

### 4.2. Biological Investigations

#### 4.2.1. Parasite Culture

The chloroquine-sensitive *Plasmodium falciparum* strain 3D7 and the chloroquine-resistant strain Dd2 were cultivated in vitro at 5% hematocrit in complete medium comprising RPMI 1640/HEPES medium supplemented with 0.5% *w*/*w* Albumax II, 10 µg/mL gentamicin, and 50 µg/mL hypoxanthine. Cultures were maintained at 37 °C in an atmosphere of 5% CO_2_, 5% O_2_, and 90% N_2_. To enrich parasites in the ring-stage, an asexual blood stage culture was synchronized. The culture was centrifuged at 1000× *g* for 3 min, and the supernatant was discarded. The cell pellet was resuspended in 5% sorbitol and incubated at room temperature for 10 min. Subsequently, the culture was centrifuged, and the pellet was washed with RPMI 1640/HEPES medium. After a final centrifugation step, the cell pellet was resuspended in fresh complete medium and returned to standard culture conditions for further cultivation. Human serum and erythrocyte concentrate were obtained from the Department of Transfusion Medicine, University Hospital Aachen, Germany. Donor sera and blood samples were pooled and kept anonymous. The work with human blood was approved by the Ethics Commission of the RWTH University Hospital (EK 007/13).

#### 4.2.2. Malstat Assay

Synchronized ring-stage cultures of the *P. falciparum* strains 3D7 and Dd2 mixed with the compound were plated in triplicate into 96-well plates (200 µL/well) at a parasitemia of 1%. Compounds were dissolved in DMSO and tested at concentrations ranging from 200 µM to 2.3 nM, utilizing an 8-point serial dilution for each compound, whereby the final DMSO concentration did not exceed 0.5% DMSO. In addition, a dilution series with chloroquine with concentrations between 500 nM and 4 nM was used as a positive control and 0.5% *v*/*v* DMSO was used as a negative control. The cultures were then incubated under standard culture conditions for 72 h. Following incubation, 20 µL of the culture was transferred in triplicate to a new 96-well plate and mixed with 100 µL of Malstat reagent (0.1% *v*/*v* Triton X-100, 1 g L-lactate, 0.33 g Tris and 33 mg of 3-acetylpyridine adenine dinucleotide in 100 mL distilled water, pH 9.0) and 20 µL mixture of NBT and diaphorase (1:1 *v*/*v*; 1 mg/mL stock each). After colour development in the wells, absorbance was measured at OD_630nm_ using a Tecan Plate Reader. The IC_50_ and IC_90_ values of the compounds were then determined using the GraphPad Prism software.

#### 4.2.3. Hemolysis Assay

Non-infected erythrocytes were adjusted to a hematocrit of 5% and mixed with the respective IC_90_ concentrations of the compounds. The detergent saponin at 0.15% *w*/*v* saponin and the solvent DMSO at 0.5% *v*/*v* served as positive and negative controls, respectively. A total of 150 µL of each sample was transferred in triplicate into a 96-well plate and incubated for 48 h at 37 °C under a controlled atmosphere (5% O_2_, 5% CO_2_, 90% N_2_). After incubation, the samples were centrifuged at 800 g for 2 min, and 100 µL of the supernatant from each well was transferred to a new 96-well plate. Hemoglobin release was quantified by measuring the optical density at 405 nm (OD_405_) using a Tecan plate reader. Data were analyzed using GraphPad Prism software.

#### 4.2.4. Cytotoxicity Assay

Human umbilical vein endothelial cells (HUVECs) were purchased from Promocell (Heidelberg, Germany) and cultivated with ECGM Kit enhanced (PELO Biotech, Planegg, Germany) supplemented with 10% (FCS) (PAA Laboratories, Cölbe, Germany) and 1% penicillin/streptomycin/amphotericin B (all purchased from PAN Biotech). Cells were cultured at 37 °C with 5% CO_2_ with constant humidity. Cell proliferation was assessed using the CellTiter-Blue cell viability assay (Promega, Madison, WI, USA). Cells were treated as indicated for 72 h and 4 h before termination CellTiter-Blue reagent was added in a ratio of 1:10. Fluorescence intensity was determined by using the SpectraFluor Plus plate reader (Tecan, Männedorf, Switzerland) at 550 nm excitation and 595 nm emission wavelength. Fluorescence intensity is proportional to the cell number. Non-linear regression was carried out using the log(inhibitor) vs. response–variable slope (four parameters) function of GraphPad Prism (San Diego, CA, USA).

## 5. Conclusions

Since 2009, more than ten cassiarin alkaloids with an unprecedented tricyclic skeleton have been isolated from *Cassia* species (Leguminosae), and among them, cassiarin A (**5**) was reported to exhibit outstanding antimalarial activity. Although a couple of synthetic approaches to cassiarins have been published, only limited data on structure–activity relationships for antimalarial activity are available. Notably, such investigations focused on *N*-alkylation of the pyridine ring and *O*-alkylation/acylation of the phenolic group, two structural elements of cassiarins which were identified to be essential for antimalarial activity.

Here we performed the first systematic variation in both methyl groups of cassiarin A (**5**) and obtained 12 analogs on three different synthesis routes. With this work we significantly extended the level of knowledge on structure–activity relationships of antiplasmodial cassiarin derivatives. While variations in the methyl group at C-5 led to a marked decrease in activity, replacement of the 2-methyl group by benzenoid residues resulted in increased antiplasmodial activity. Testing against the chloroquine-resistant *P. falciparum* strain Dd2 demonstrated low resistance index values for cassiarin A (RI = 1.4) and analogs **11d** and **18c** (both RI = 2.0). This underlines that especially by the introduction of an aromatic ring at C-2 the in vitro antiplasmodial profile of cassiarins can be improved significantly. In order to explore the therapeutic potential of these new analogs further, we investigated the cytotoxicity of representative compounds. Unfortunately, the selected compounds cassiarin A, **11c** and **11d** showed significant cytotoxicity against the human cell line HUVEC. These results suggest that cytotoxicity against non-cancerous human cells might be a serious obstacle to the future development of this chemotype for antimalarial therapy.

## Figures and Tables

**Table 1:** Antiplasmodial activity of cassiarin A and analogs against the chloroquine-sensitive *P. falciparum* strain 3D7.

Compound	*P. falciparum* 3D7 *^a^* IC_50_ *^b^* [µM]	*P. falciparum* 3D7 *^a^* IC_90_ *^c^* [µM]
**5** (cassiarin A)	9.6 ± 0.63	16.4 ± 3.07
**11a**	31.9 ± 11.47	53.3 ± 10.49
**11b**	2.0 ± 0.18	2.9 ± 0.53
**11c**	0.4 ± 0.04	0.5 ± 0.11
**11d**	0.6 ± 0.11	1.0 ± 0.28
**11e**	2.1 ± 0.38	2.7 ± 0.22
**11f**	1.7 ± 0.20	3.4 ± 1.28
**11g**	0.4 ± 0.03	0.9 ± 0.54
**18a**	1.9 ± 0.19	2.5 ± 0.49
**18b**	2.2 ± 0.58	3.2 ± 0.68
**18c**	0.2 ± 0.05	0.4 ± 0.14
**24**	≥100	≥100
**2** (Chloroquine)	0.03 ± 0.01	0.05 ± 0.01

*^a^ *3D7 = *P. falciparum* chloroquine-sensitive strain. *^b^ *IC_50_, half-maximal inhibitory concentration. *^c^ *IC_90_, 90% of the maximal inhibitory concentration.

**Table 2:** Antiplasmodial activity of cassiarin A and analogs against the chloroquine-resistant *P. falciparum* strain Dd2.

Compound	IC_50_ *^b^* [µM] *P. falciparum* Dd2 *^a^*	IC_90_ *^c^* [µM] *P. falciparum* Dd2 *^a^*
**5** (cassiarin A)	13.6 ± 3.95	20.5 ± 11.88
**11c**	1.7 ± 0.74	2.1 ± 1.08
**11d**	1.2 ± 0.32	1.8 ± 0.17
**11g**	1.9 ± 1.01	5.9 ± 3.42
**18c**	0.4 ± 0.21	0.8 ± 0.58
**2** (chloroquine)	0.16 ± 0.04	0.23 ± 0.13

*^a^ *Dd2, *P. falciparum* chloroquine-resistant strain. *^b^ *IC_50_, half-maximal inhibitory concentration. *^c^ *IC_90_, 90% of the maximal inhibitory concentration.

**Table 3:** Resistance indexes for the most active compounds.

Compound	RI ^a^
**5** (cassiarin A)	1.4
**11c**	4.3
**11d**	2.0
**11g**	4.8
**18c**	2.0
**2** (chloroquine)	5.3

^a^ RI, resistance index = IC_50_ (Dd2)/IC_50_ (3D7).

## Data Availability

The experimental data, spectra, and protocols are stored in an electronic lab journal by the authors.

## Supplementary Materials

The following supporting information can be downloaded at: https://www.mdpi.com/article/10.3390/ph18071018/s1: NMR data of cassiarin A, its synthetic analogs and precursors.
