# Supplementary material for: Synthetic Analogs of the Alkaloid Cassiarin A with Enhanced Antimalarial Activity
Source: Pharmaceuticals (Basel). 2025 Jul 9;18(7):1018. doi: 10.3390/ph18071018 (PMC12299140; doi:10.3390/ph18071018)

SUPPORTING INFORMATION

Synthetic analogues of the alkaloid cassiarin A with enhanced antimalarial activity

Thomas Klaßmüller[1], Timo Reiß[2], Florian Lengauer[3], Che Julius Ngwa[2], Karin Bartel[4], Gabriele Pradel[2] and Franz Bracher[1,*]

[1] Department of Pharmacy - Center for Drug Research, Pharmaceutical Chemistry, Ludwig-Maximilians University, Munich, Germany
[2] Division of Cellular and Applied Infection Biology, RWTH Aachen University, 52074 Aachen, Germany
[3] Department of Pharmacy - Center for Drug Research, Pharmaceutical Biology, Ludwig-Maximilians University, Munich, Germany

Content:

NMR data of cassiarin A, its synthetic analogues and precursors

^{1}H NMR spectrum of cassiarine A (5) (400 MHz, DMSO-d_6)

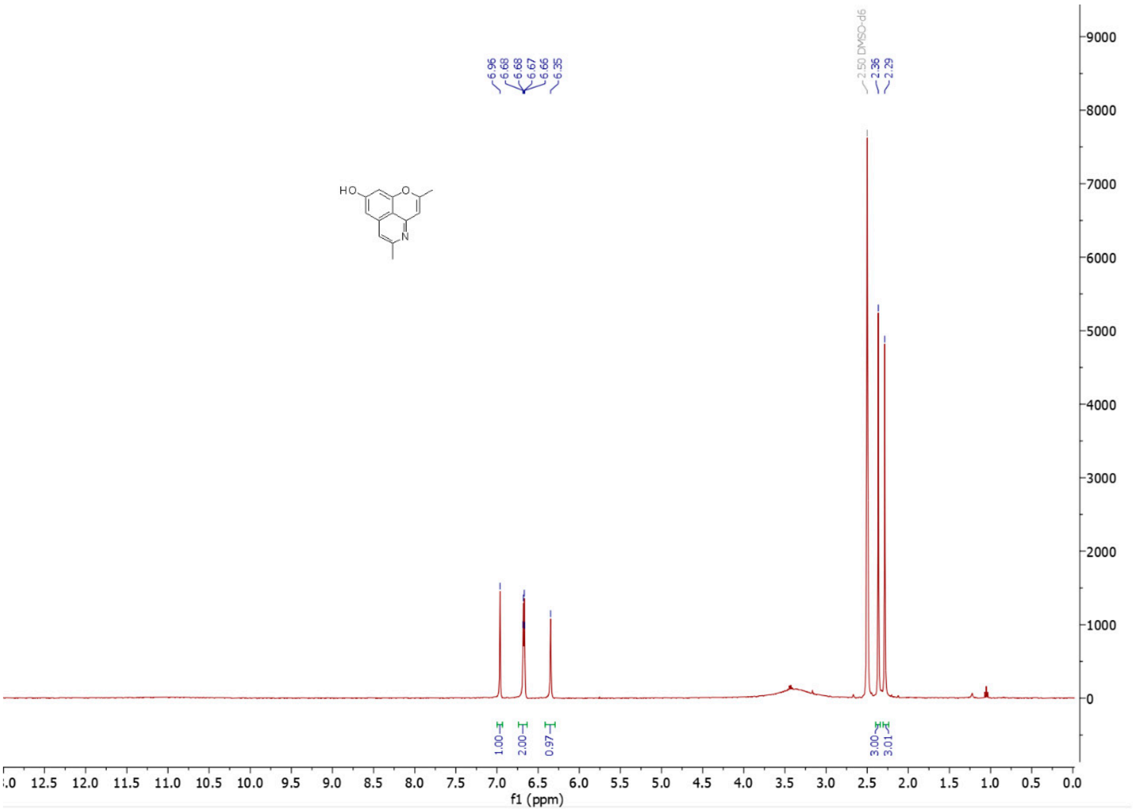

^{13}C NMR spectrum of cassiarine A (5) (101 MHz, DMSO-d_6)

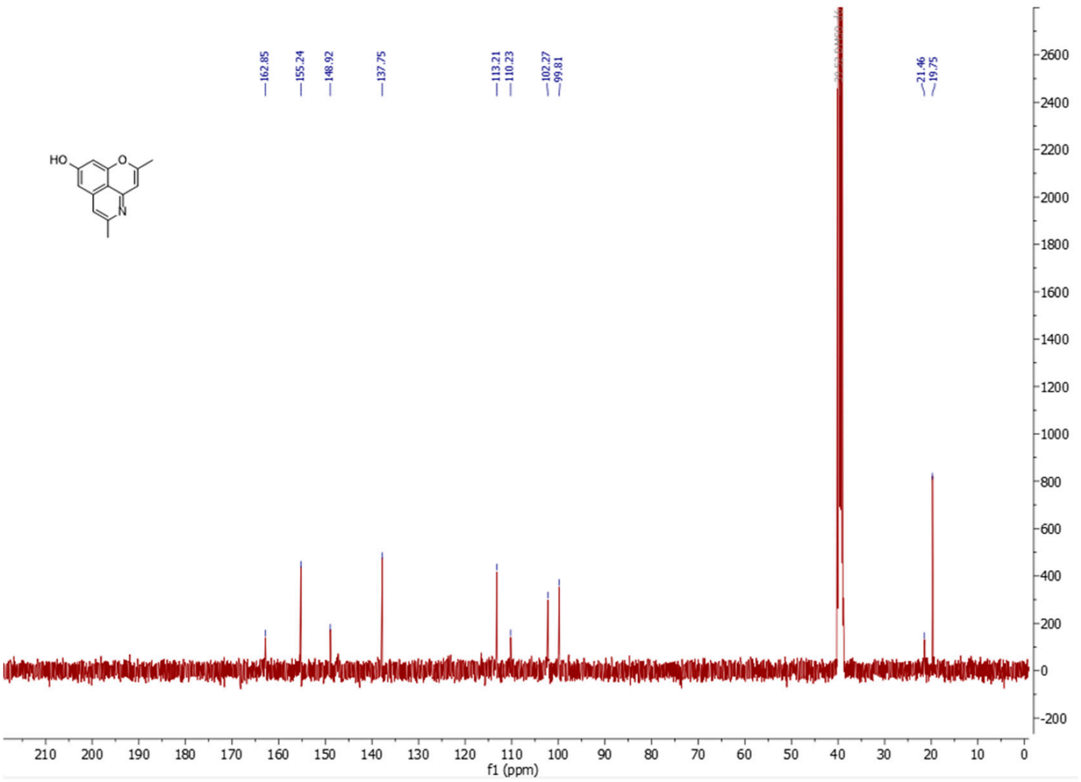

^{1}H NMR spectrum of compound 11a (400 MHz, DMSO-d_6)

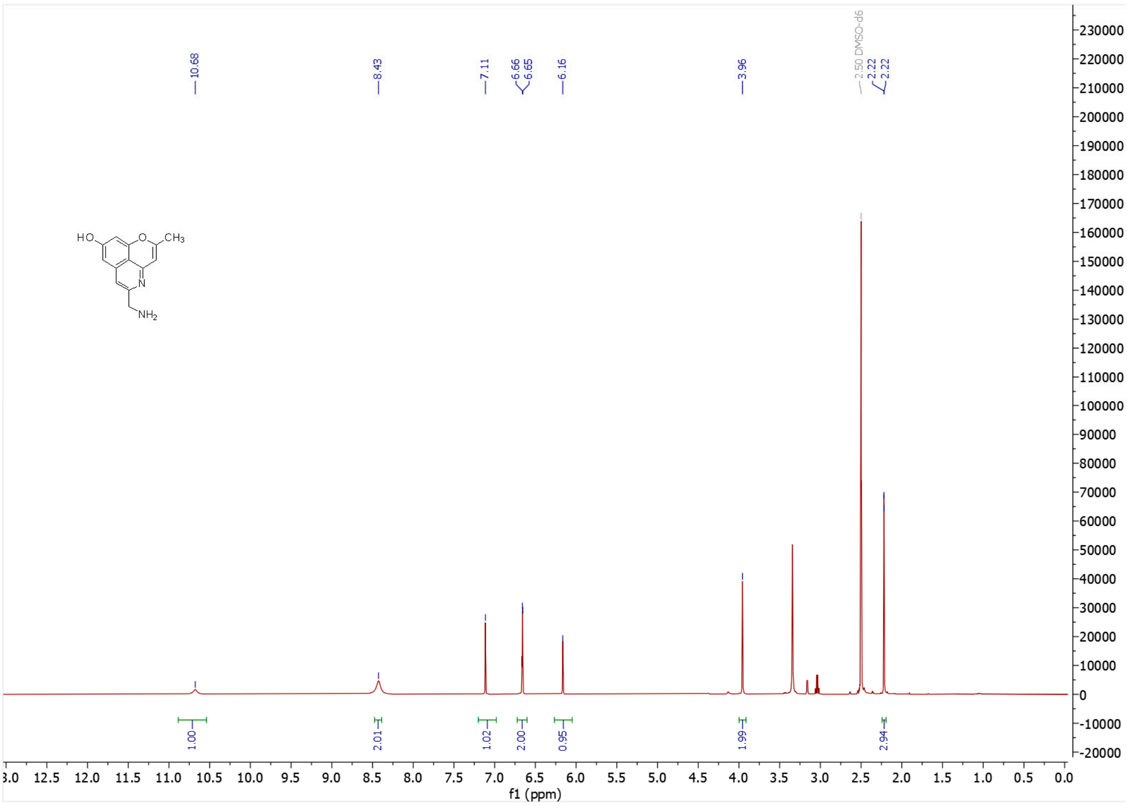

^{13}C NMR spectrum of compound 11a (101 MHz, DMSO-d_6)

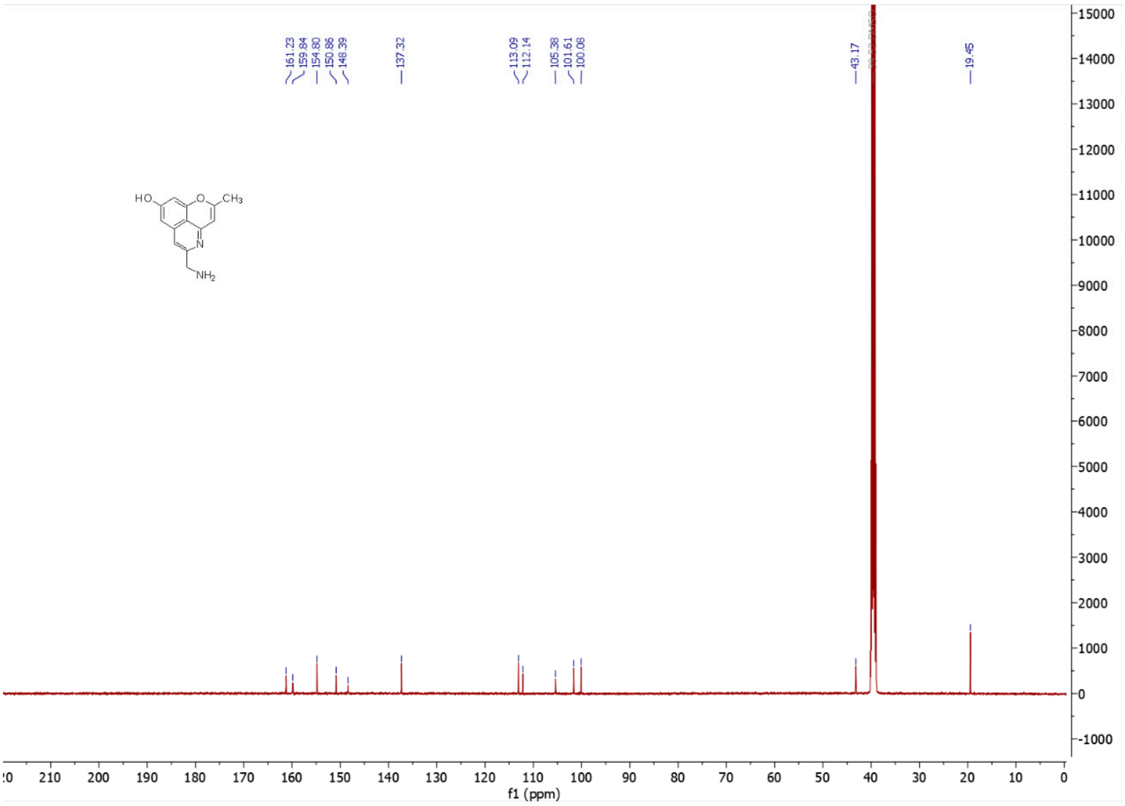

^{1}H NMR spectrum of compound 11b (400 MHz, DMSO-d_6)

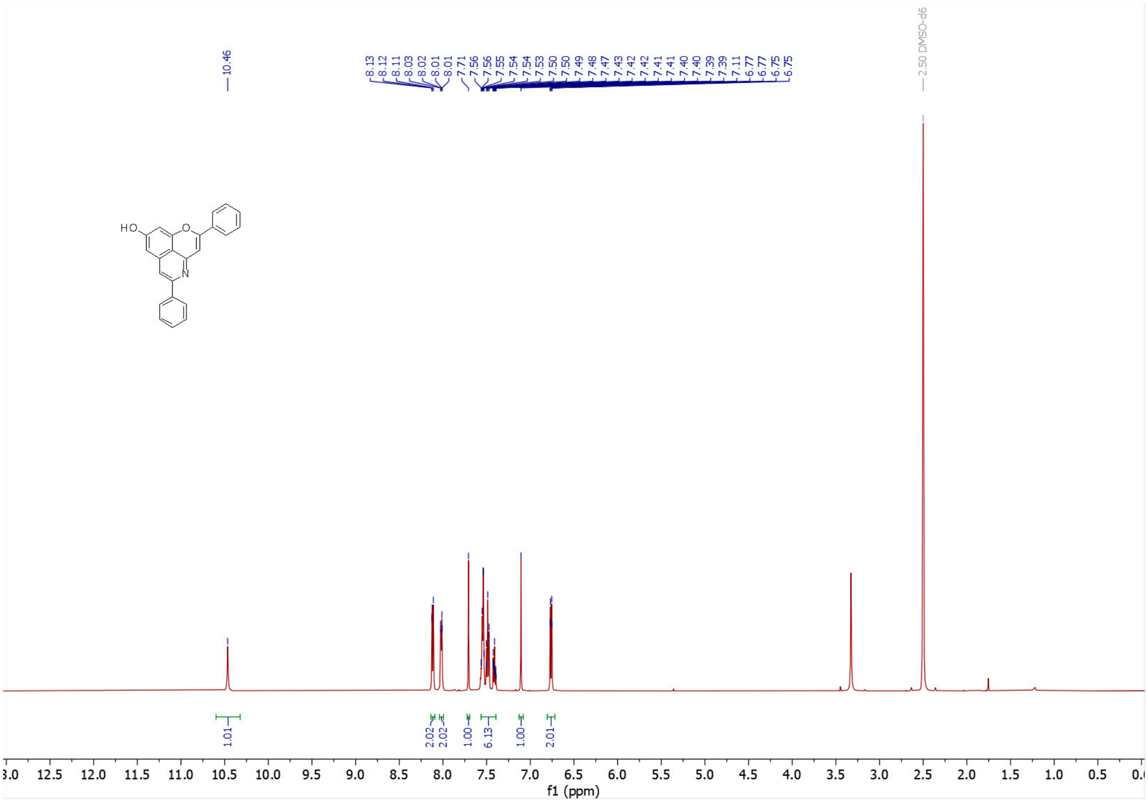

^{13}C NMR spectrum of compound 11b (101 MHz, DMSO-d_6)

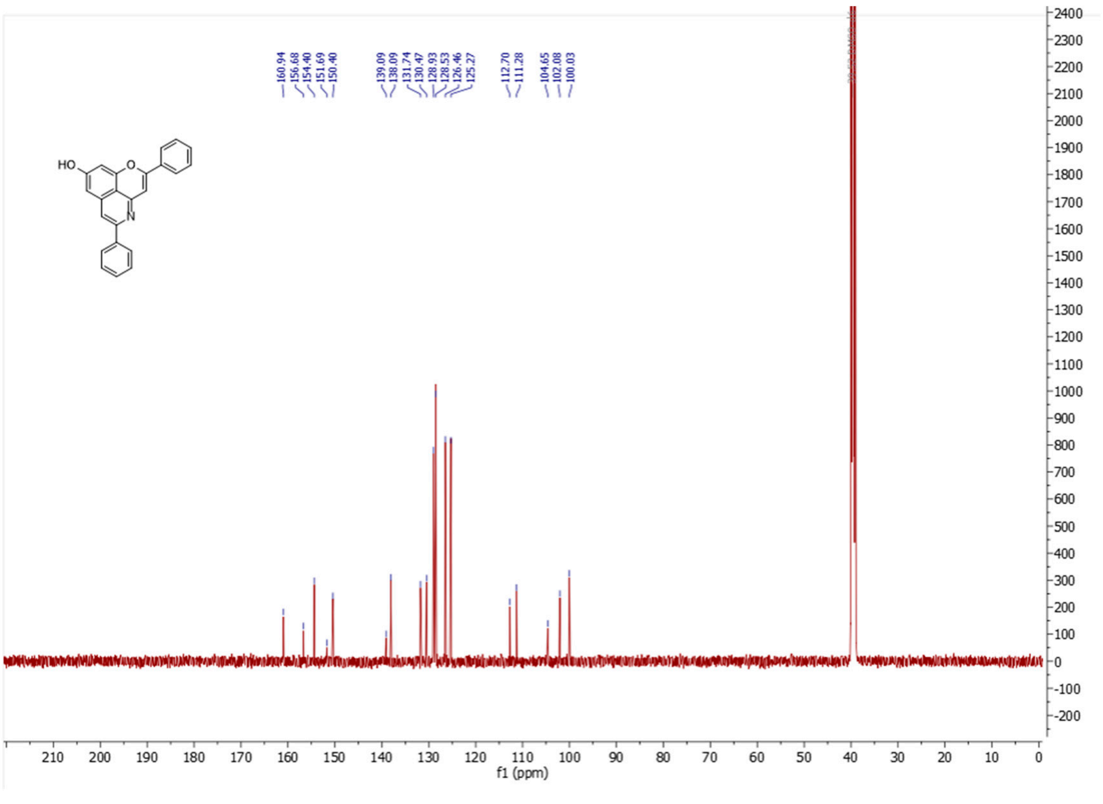

^{1}H NMR spectrum of compound 11c (400 MHz, DMSO-d_6)

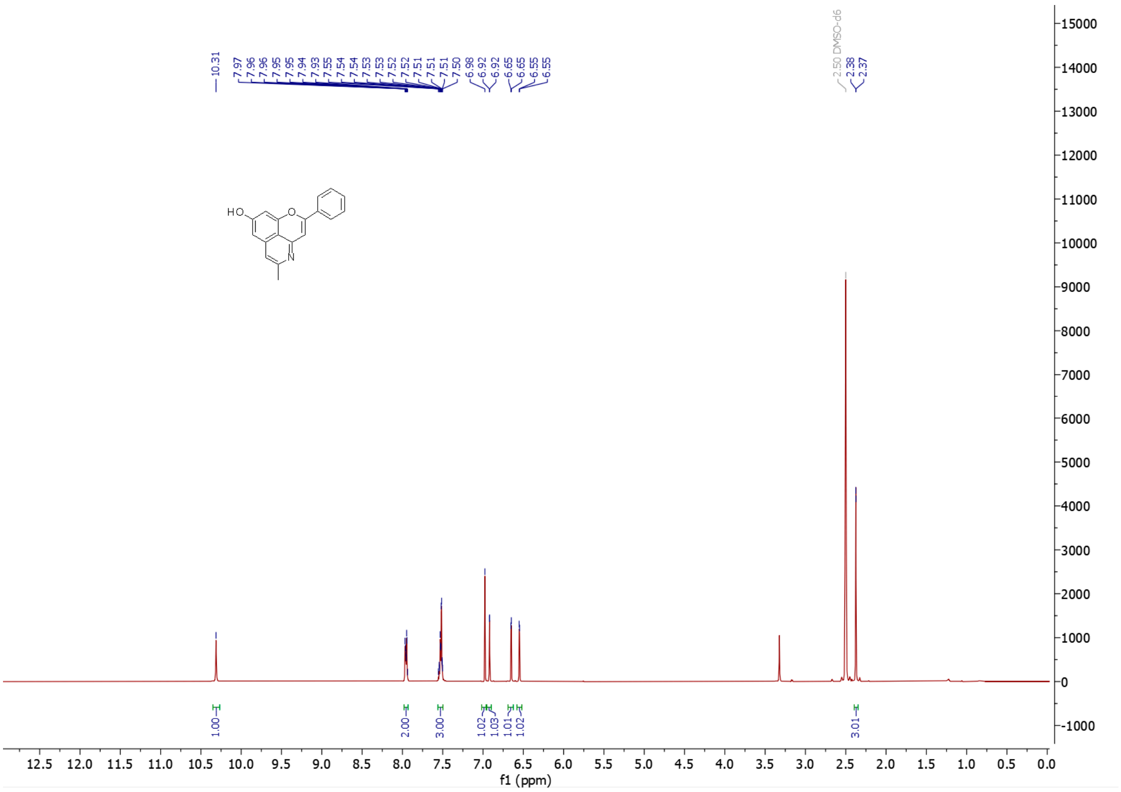

^{13}C NMR spectrum of compound 11c (101 MHz, DMSO-d_6)

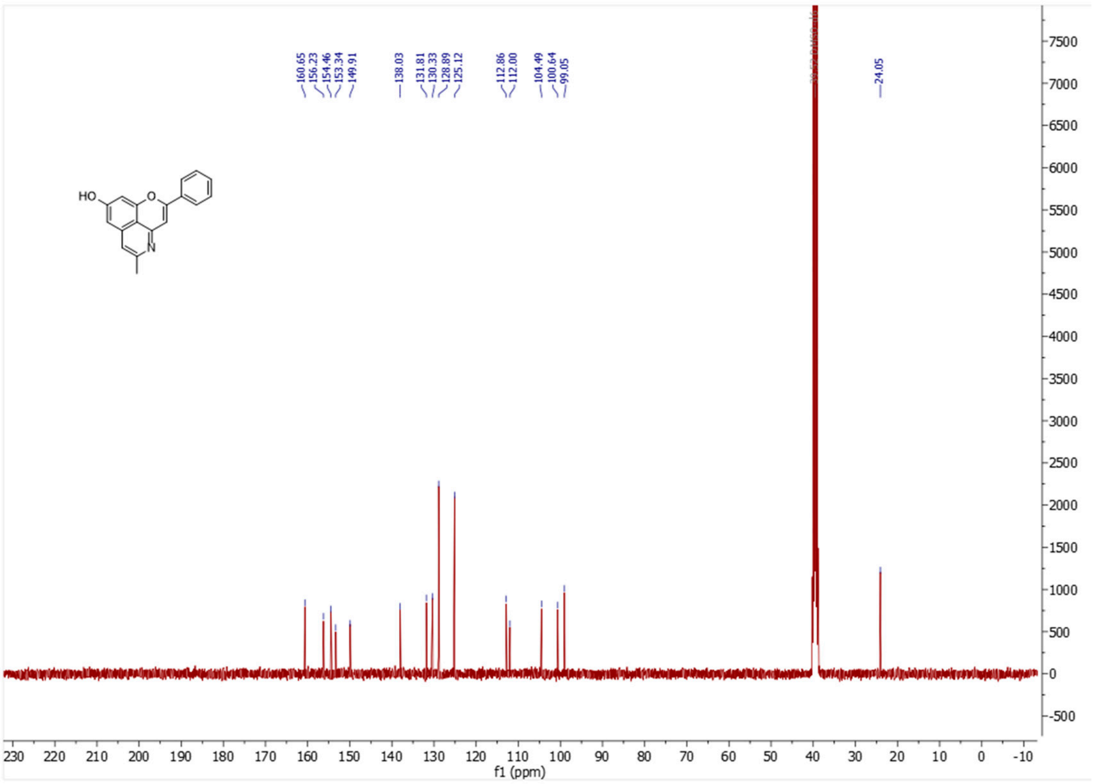

^{1}H NMR spectrum of compound 11d (400 MHz, CD_3OD)

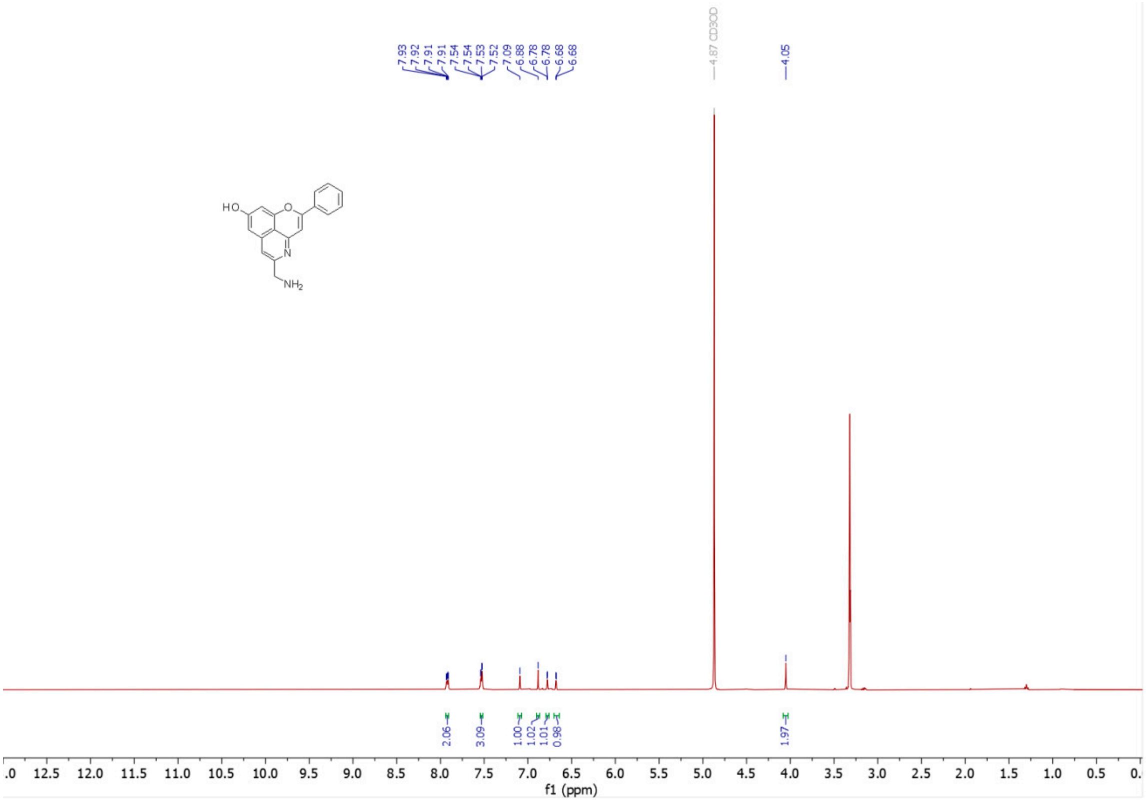

^{13}C NMR spectrum of compound 11d (101 MHz, CD_3OD)

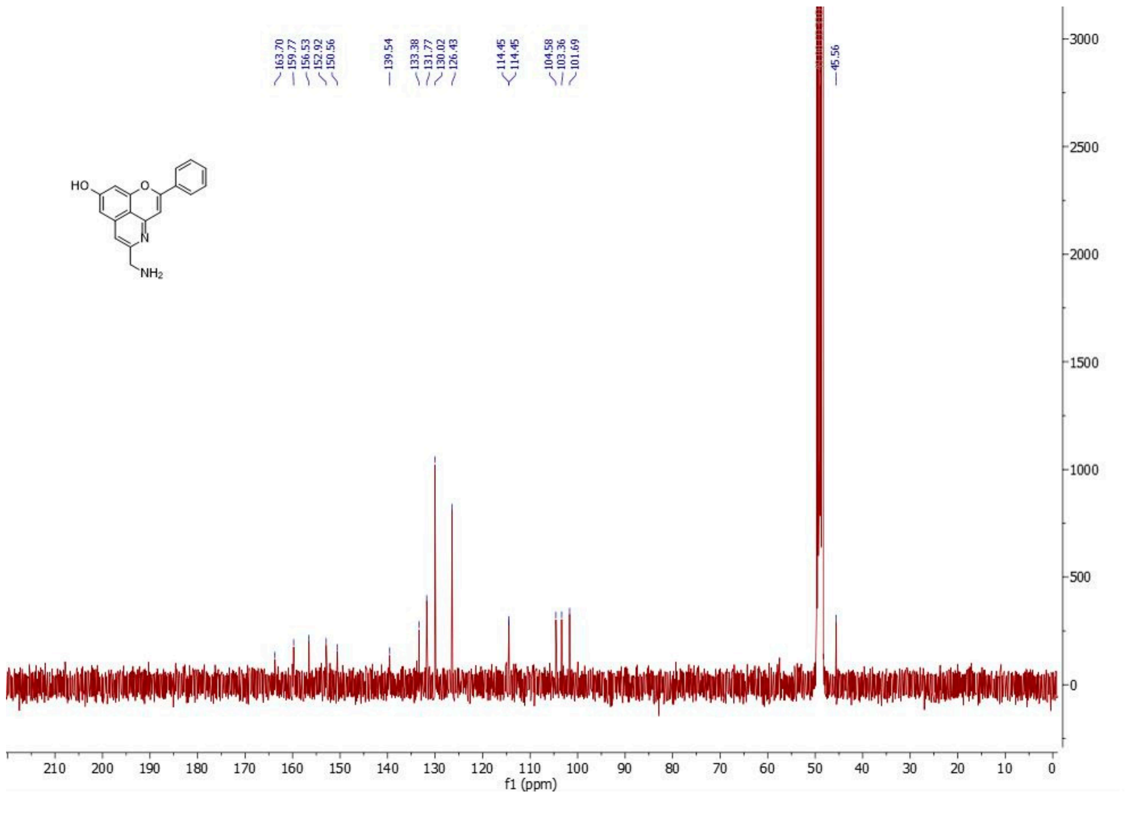

^{1}H NMR spectrum of compound 11e (400 MHz, CD_3OD)

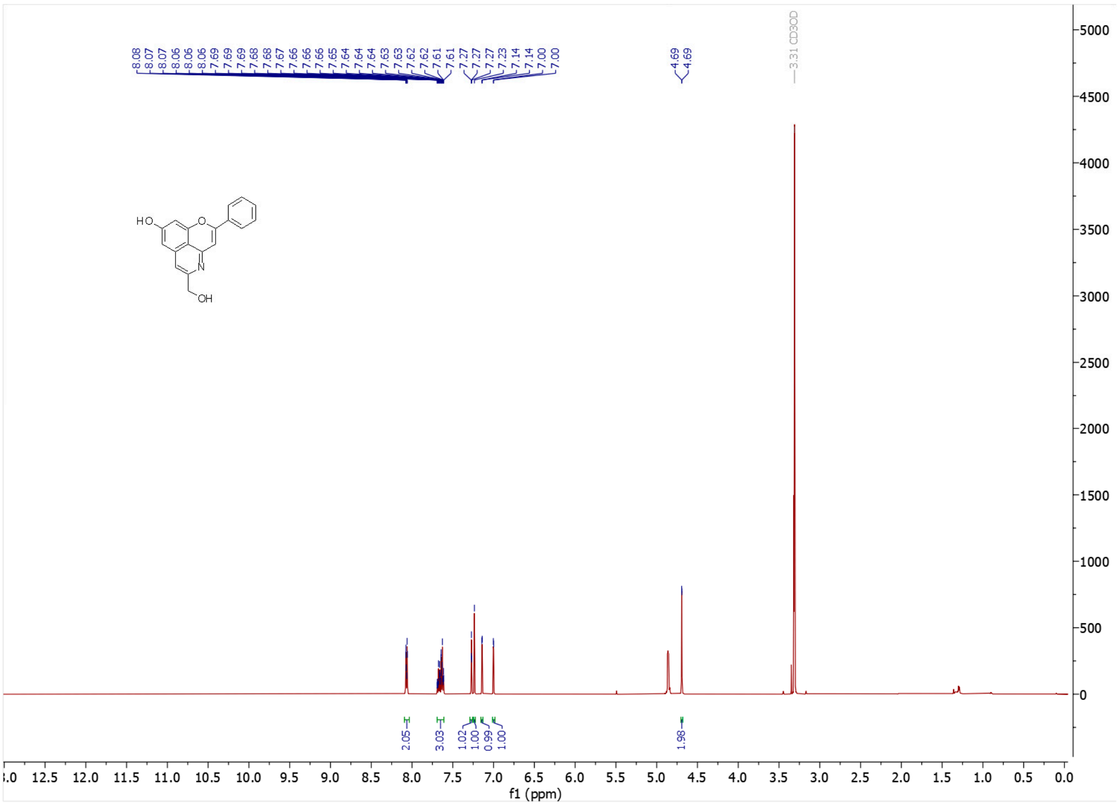

^{13}C NMR spectrum of compound 11e (101 MHz, CD_3OD)

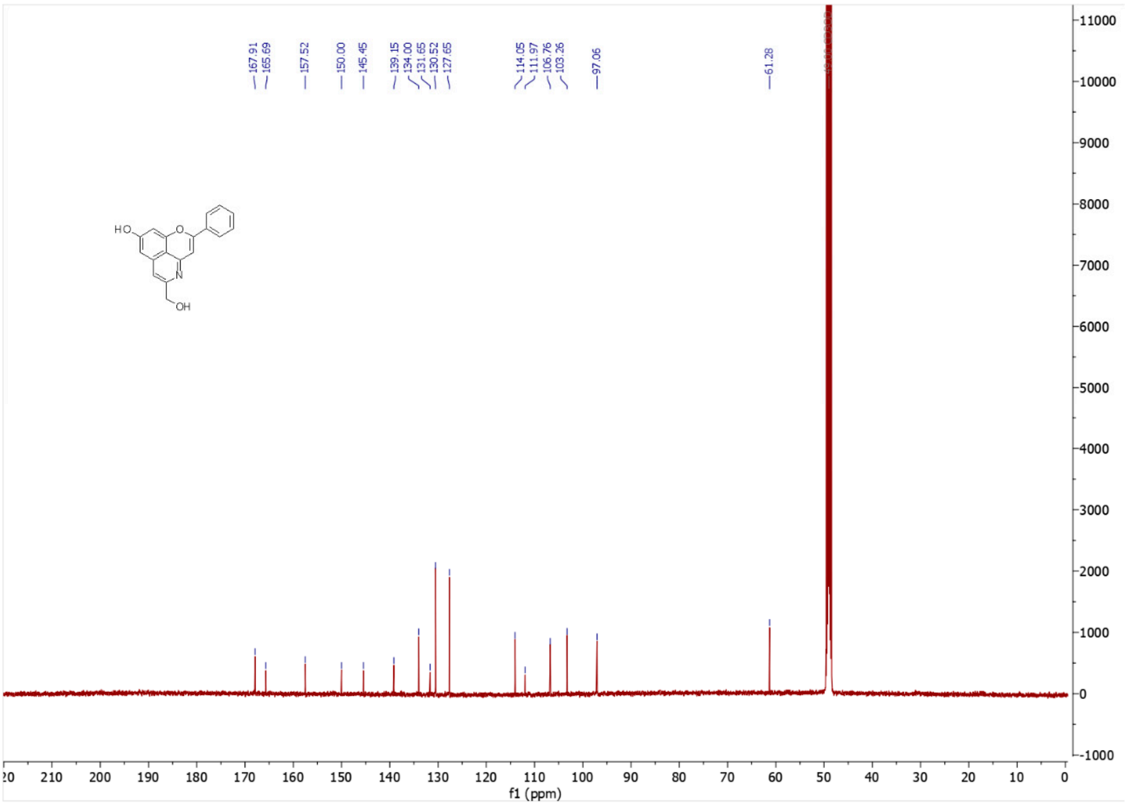

^{1}H NMR spectrum of compound 11f (400 MHz, CD_3OD)

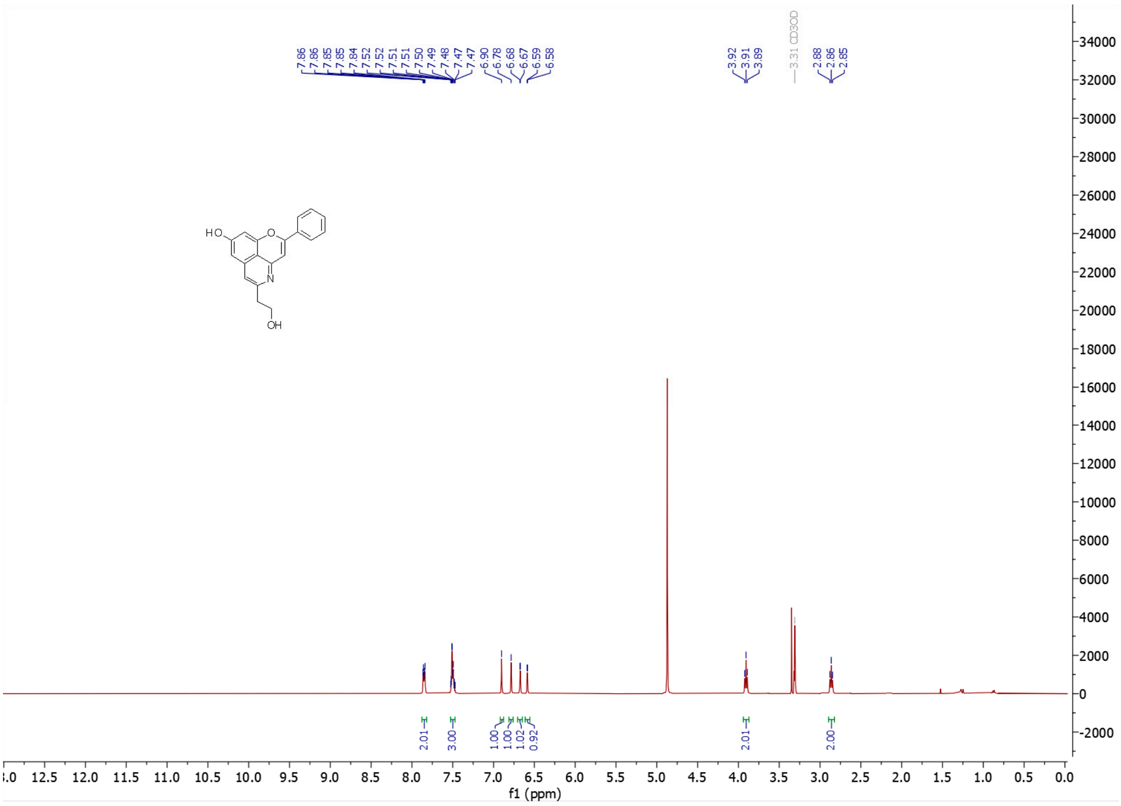

^{13}C NMR spectrum of compound 11f (101 MHz, CD_3OD)

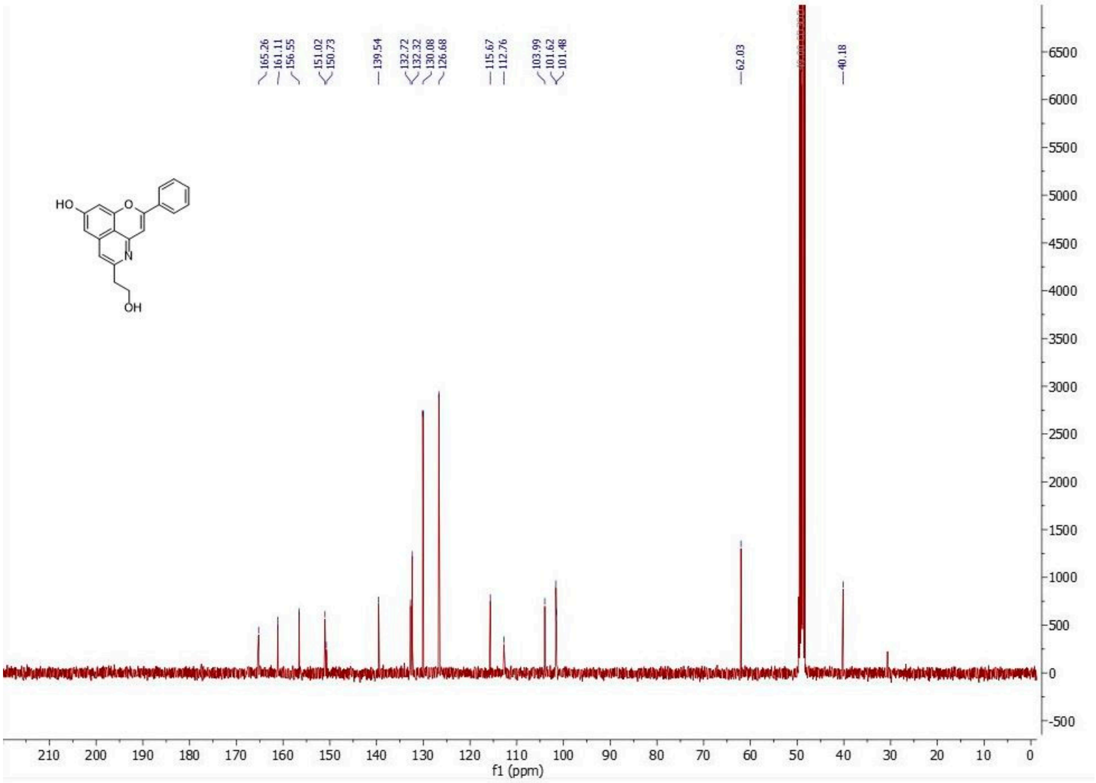

^{1}H NMR spectrum of compound 11g (400 MHz, DMSO-d_6)

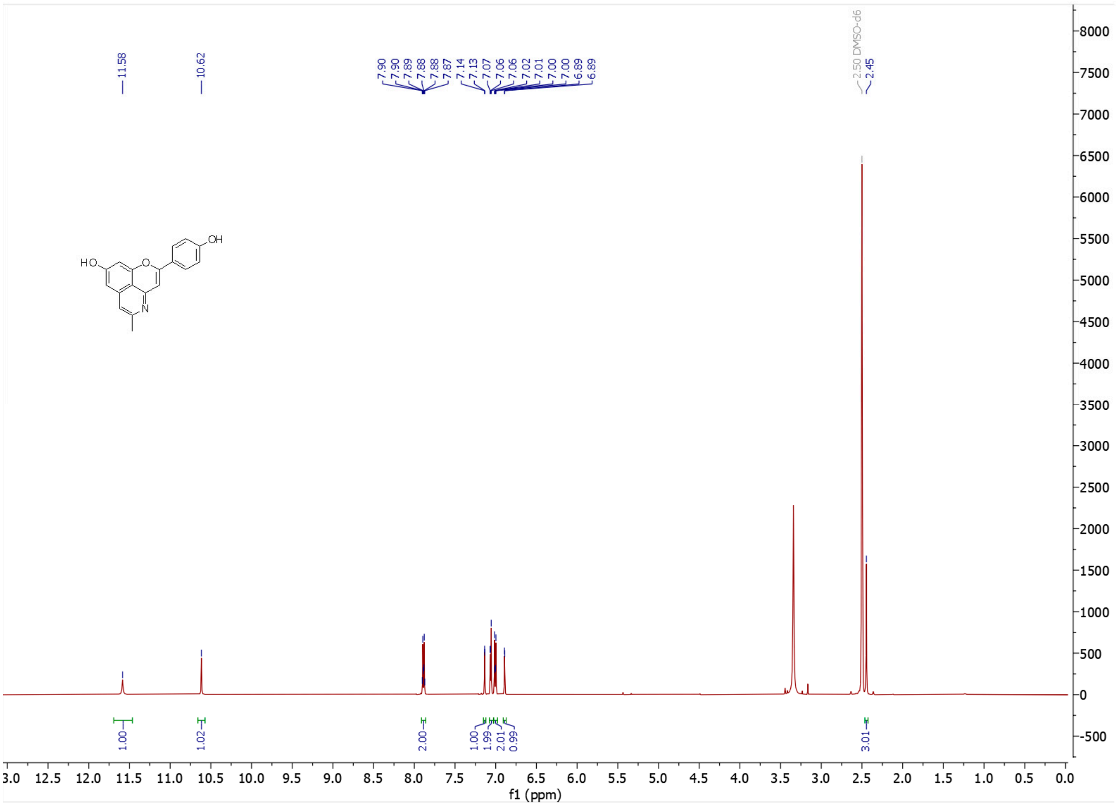

^{13}C NMR spectrum of compound 11g (101 MHz, DMSO-d_6)

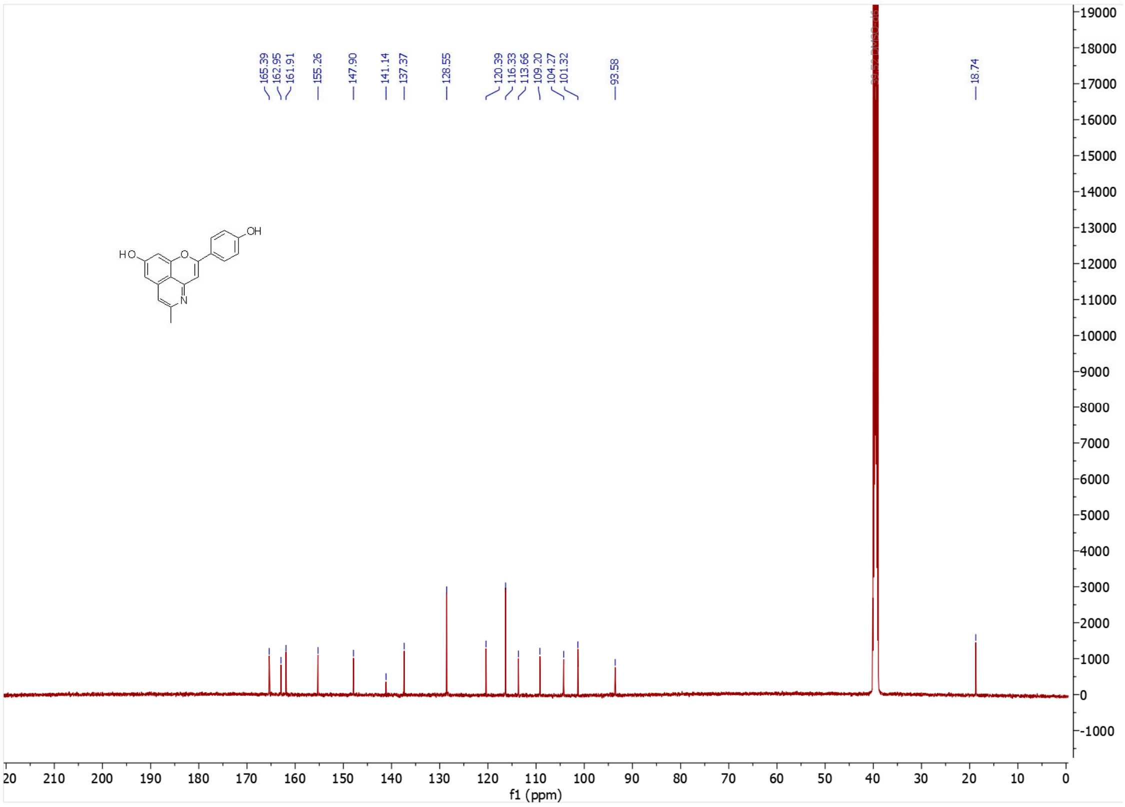

^{1}**H NMR spectrum of compound 18a** (400 MHz, DMSO-d_6)

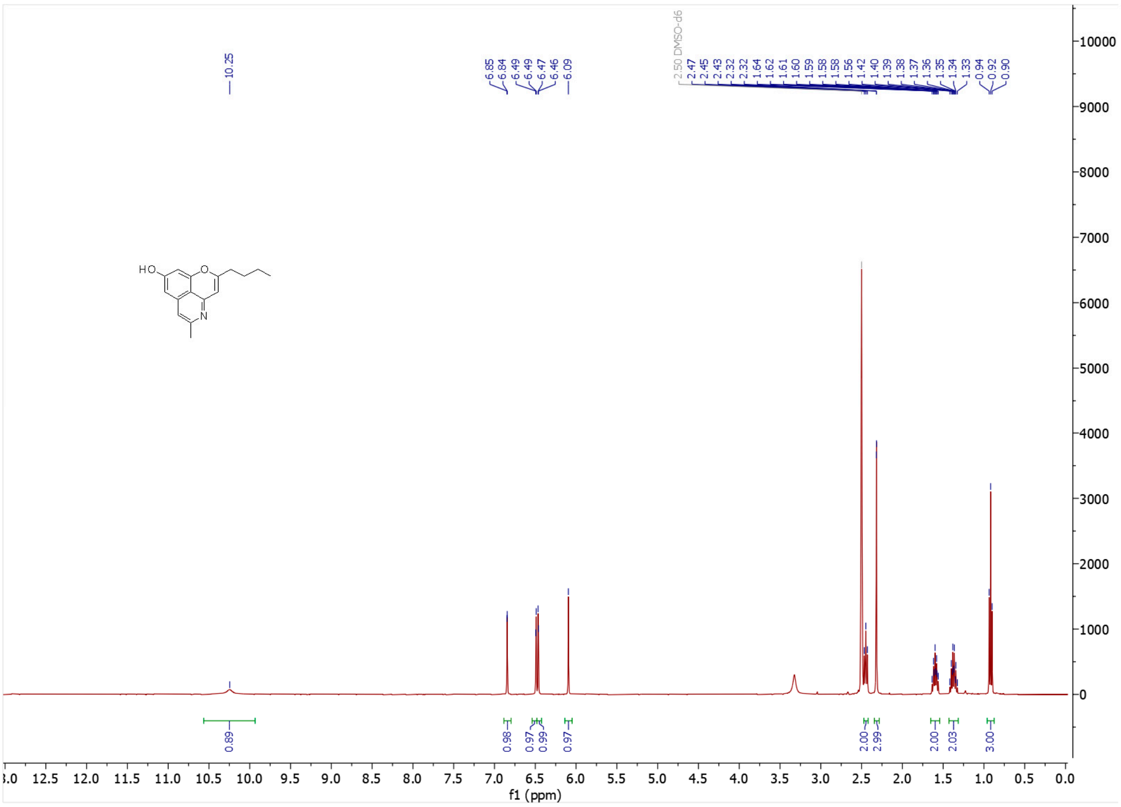

^{13}**C NMR spectrum of compound 18a** (101 MHz, DMSO-d_6)

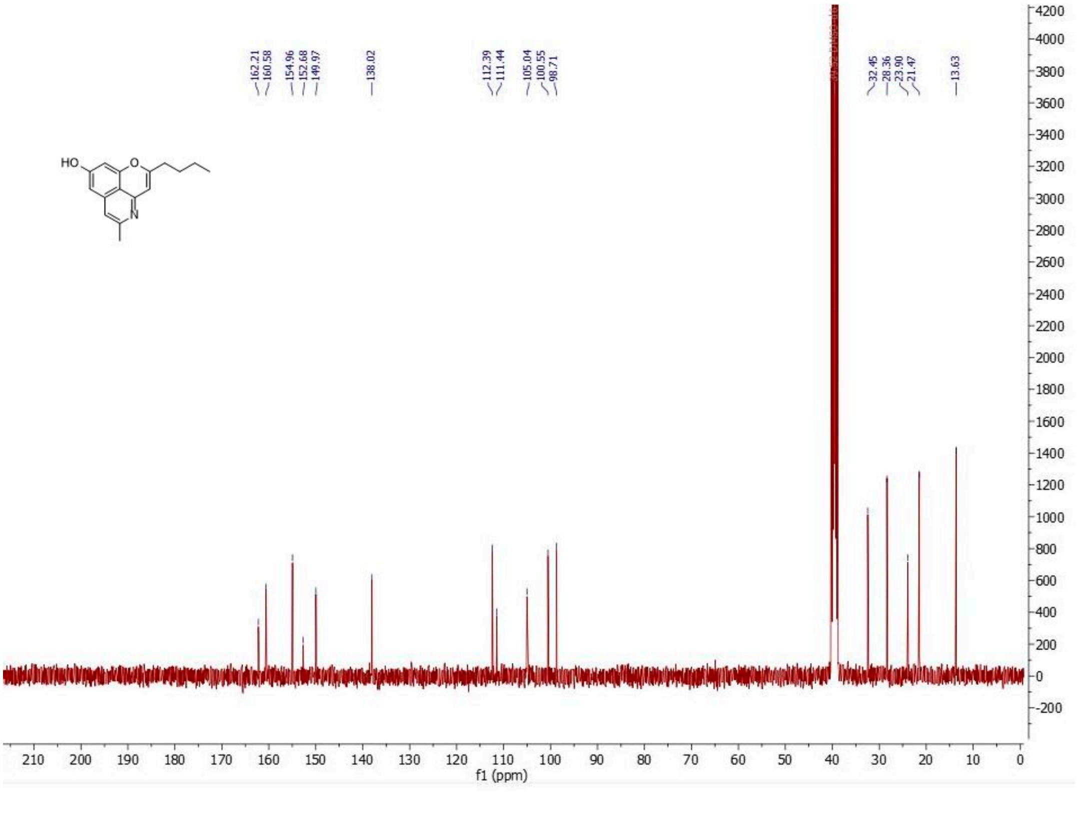

^{1}H NMR spectrum of compound 18b (400 MHz, CD_2Cl_2)

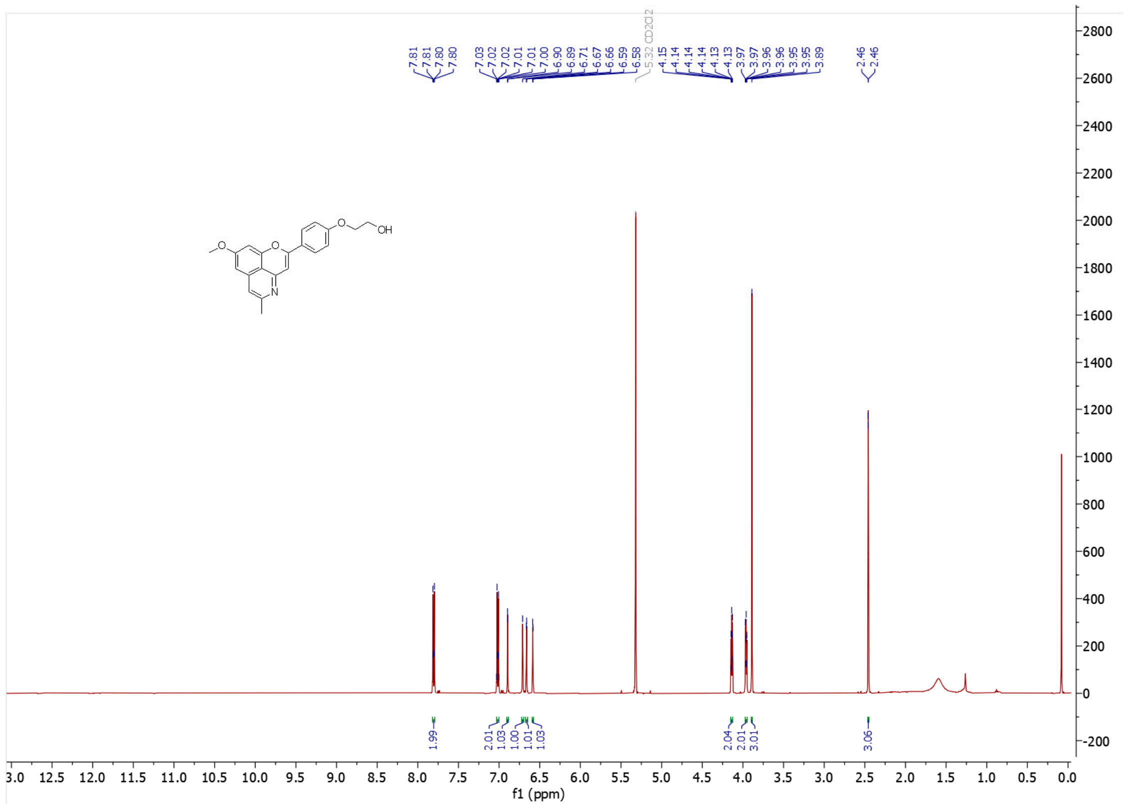

^{13}C NMR spectrum of compound 18b (101 MHz, CD_2Cl_2)

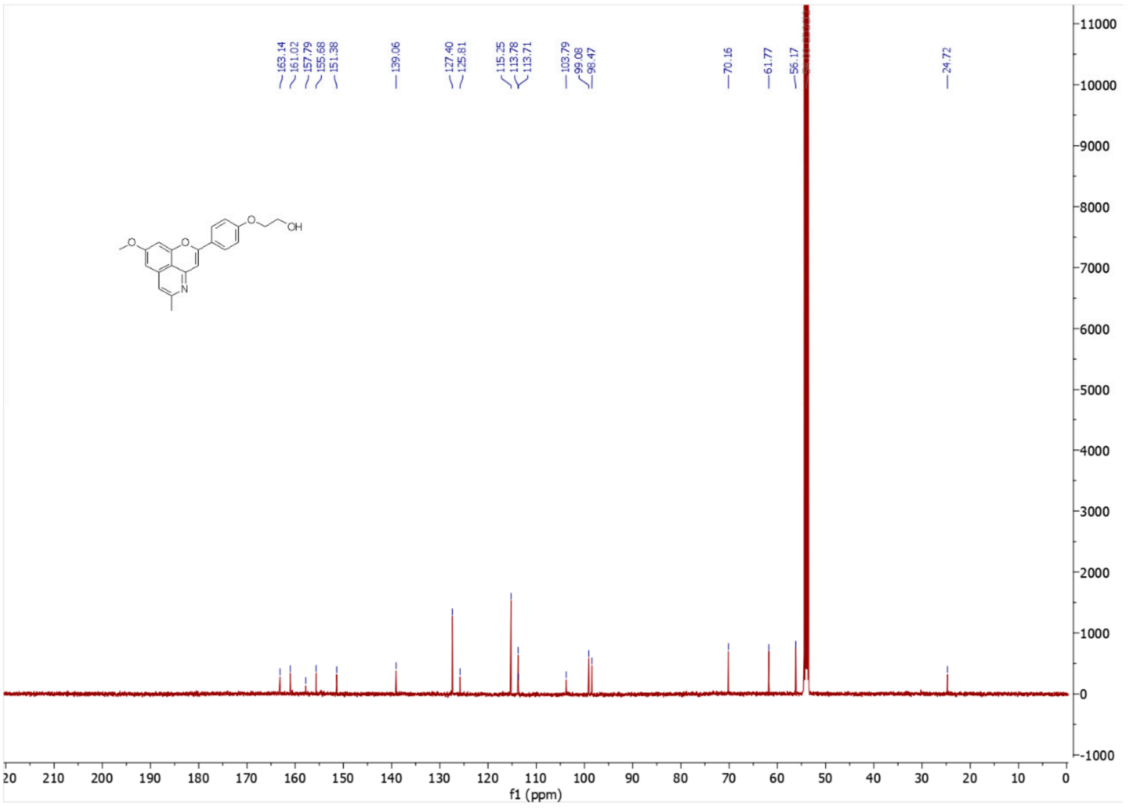

^{1}H NMR spectrum of compound 18c (400 MHz, DMSO-d_6)

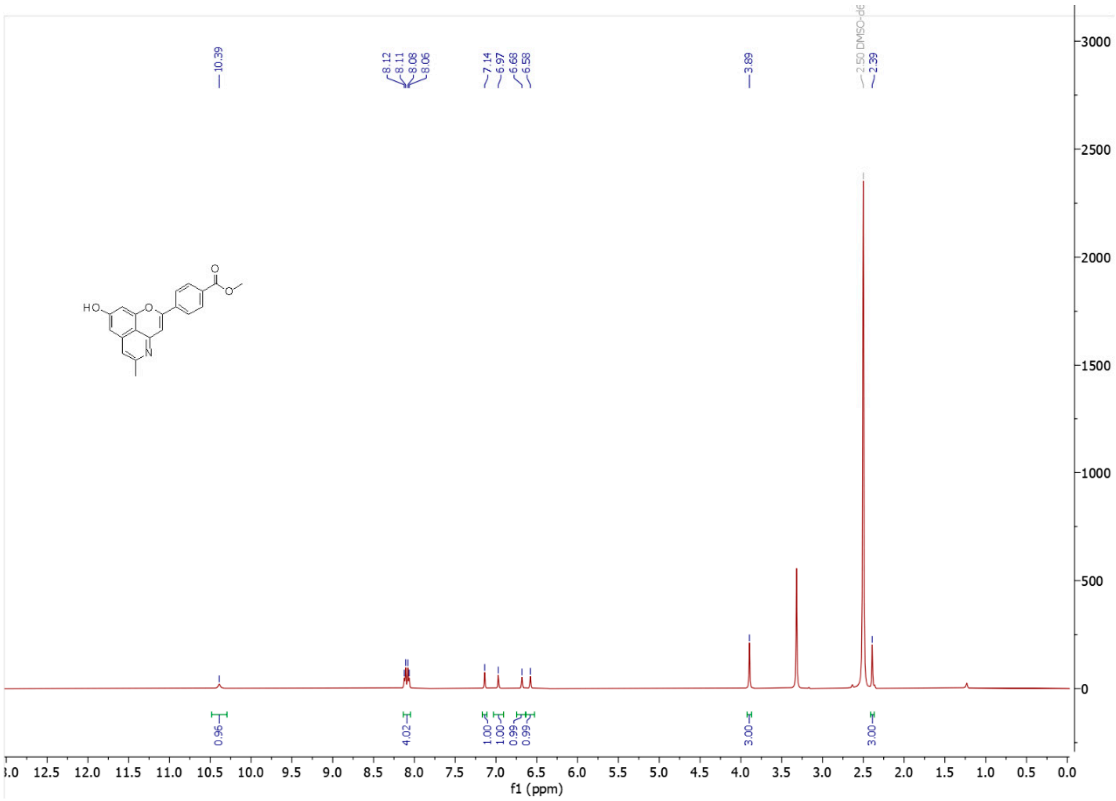

^{13}C NMR spectrum of compound 18c (101 MHz, DMSO-d_6)

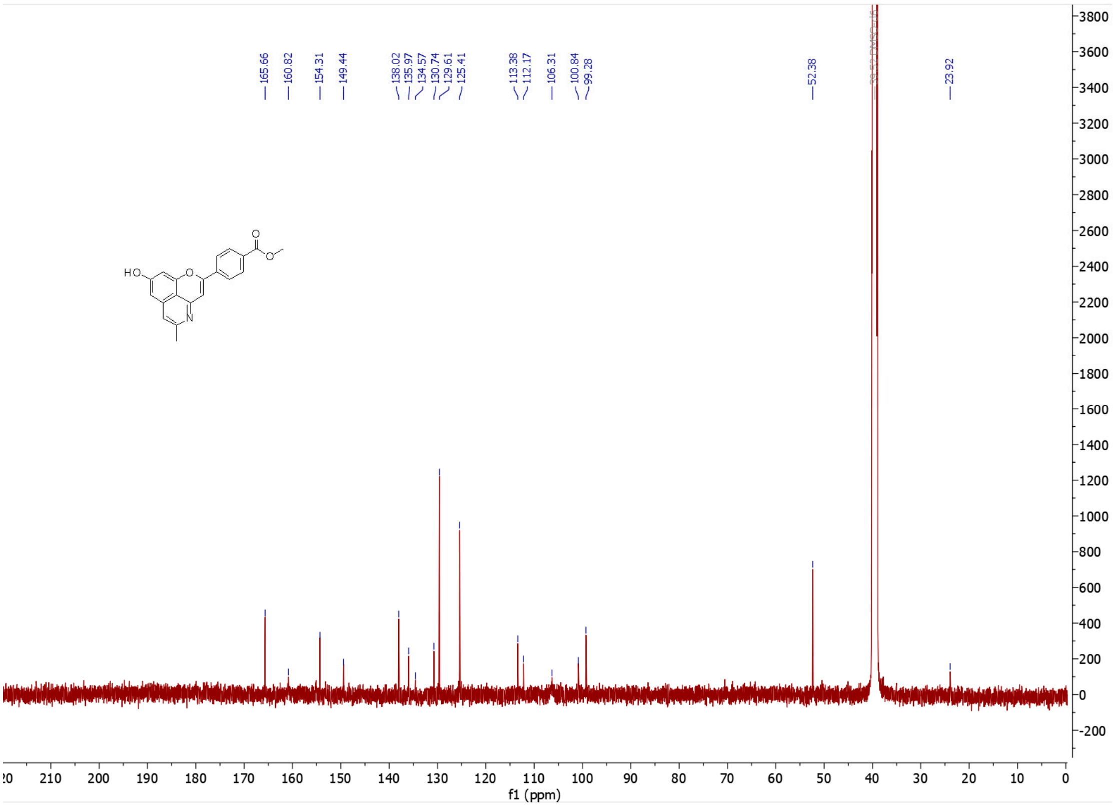

^{1}H NMR spectrum of compound 24 (400 MHz, CD_2Cl_2)

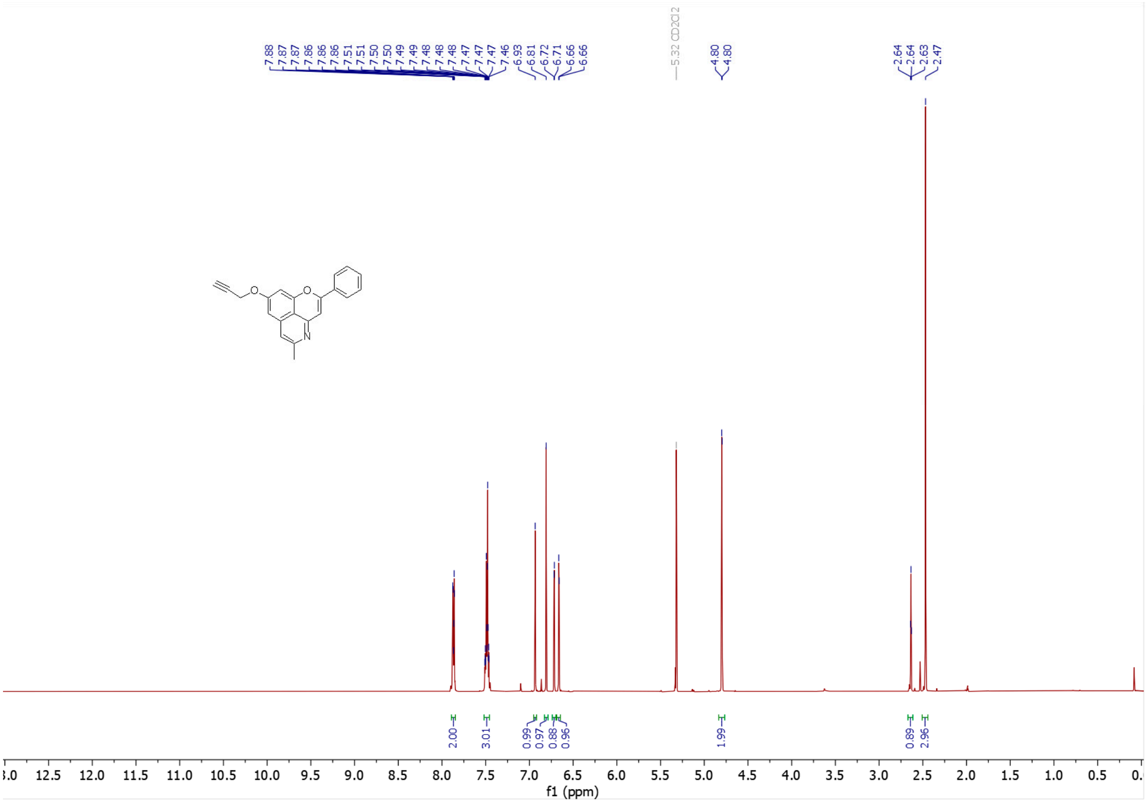

^{13}C NMR spectrum of compound 24 (101 MHz, CD_2Cl_2)

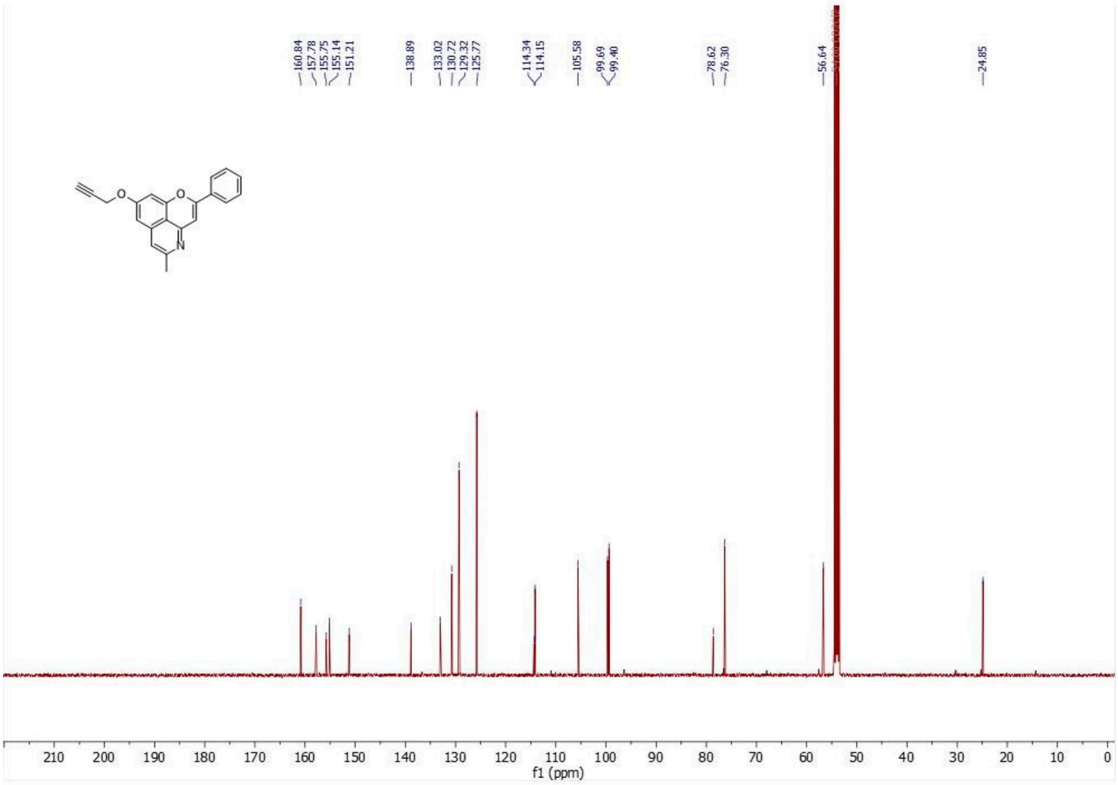

NMR spectra of precursors (not included are precursors which were directly used for the next step in crude form)

^{1}H NMR spectrum of compound 10a (400 MHz, $CDCl_3$)

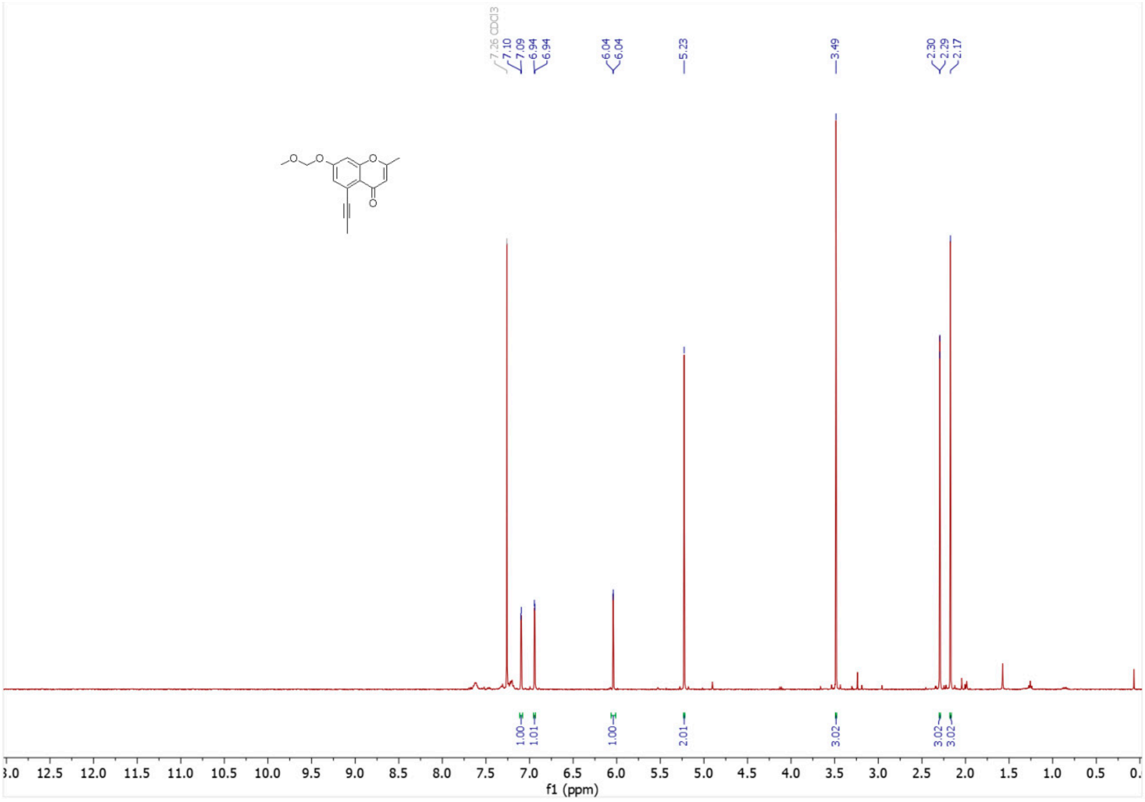

^{13}C NMR spectrum of compound 10a (101 MHz, $CDCl_3$)

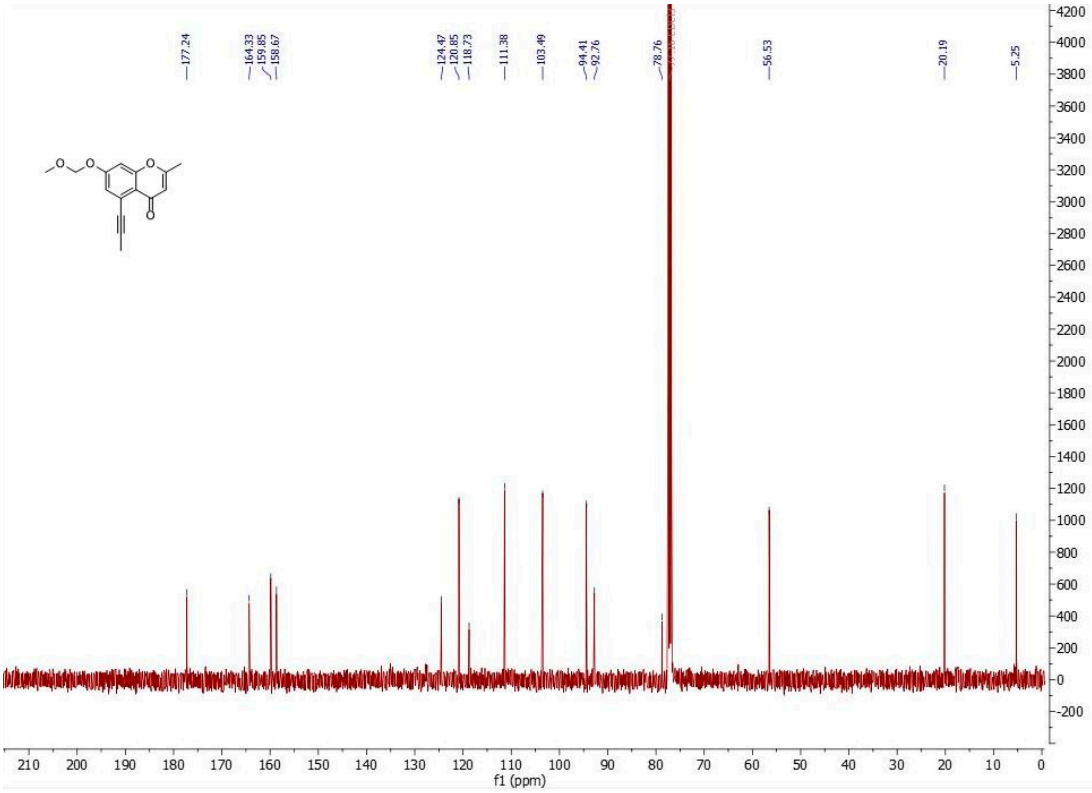

^{1}H NMR spectrum of compound 10c (400 MHz, $CDCl_3$)

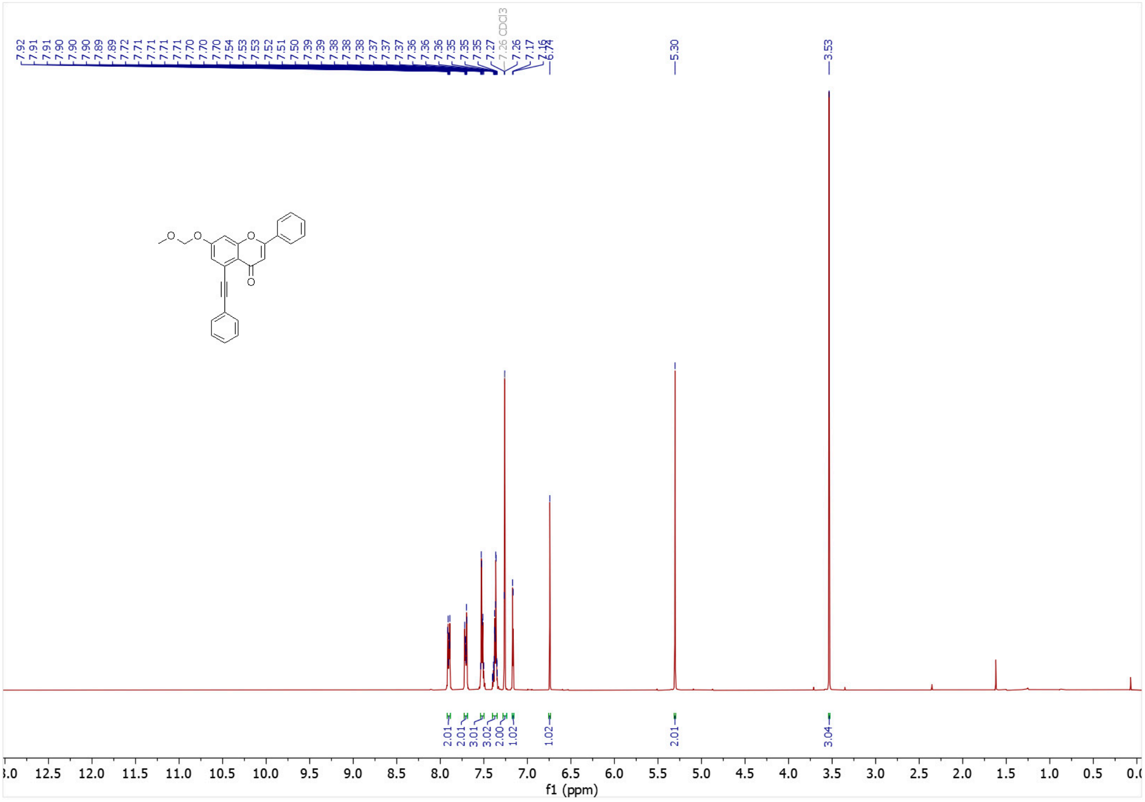

^{13}C NMR spectrum of compound 10c (101 MHz, $CDCl_3$)

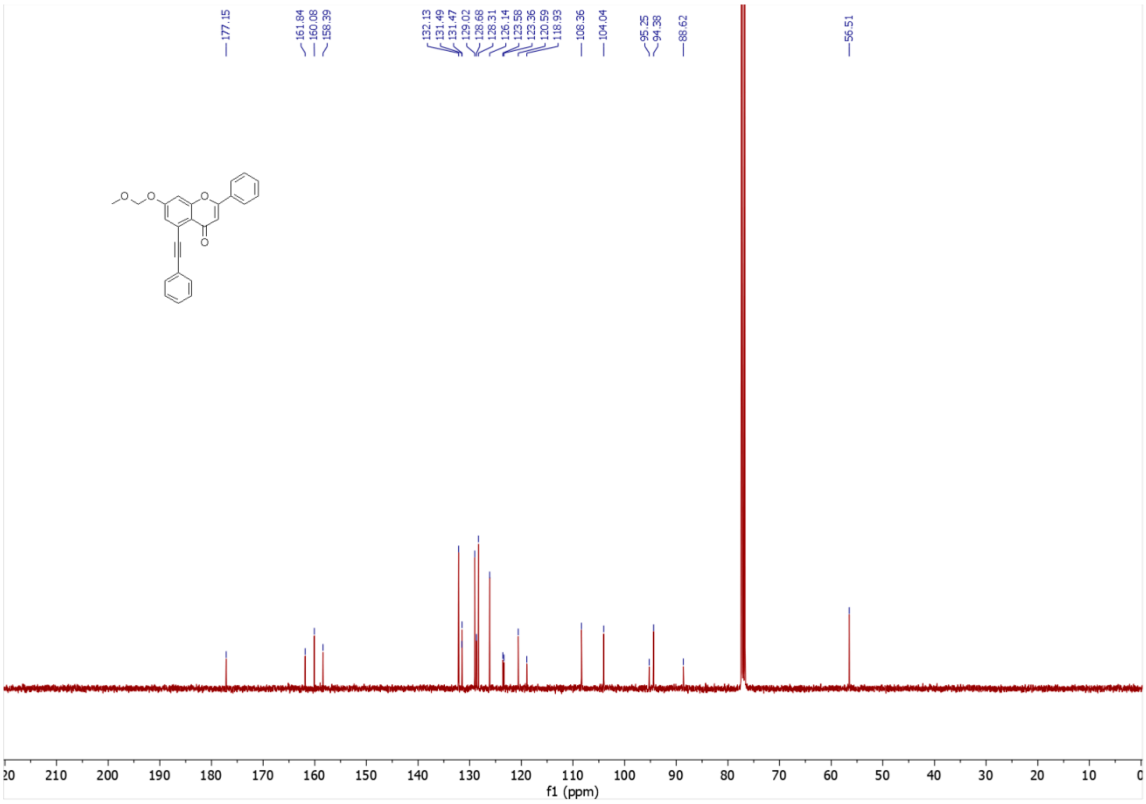

^{1}H NMR spectrum of compound 10d (400 MHz, DMSO-d_6)

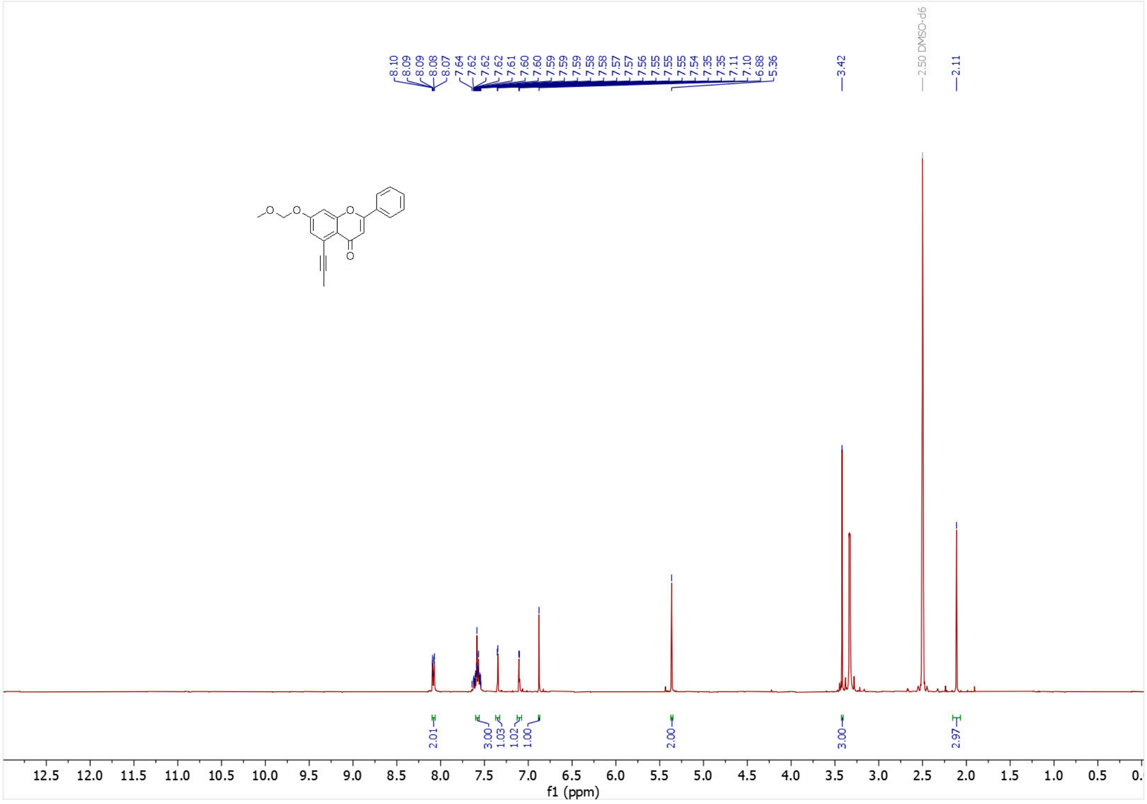

^{13}C NMR spectrum of compound 10d (101 MHz, DMSO-d_6)

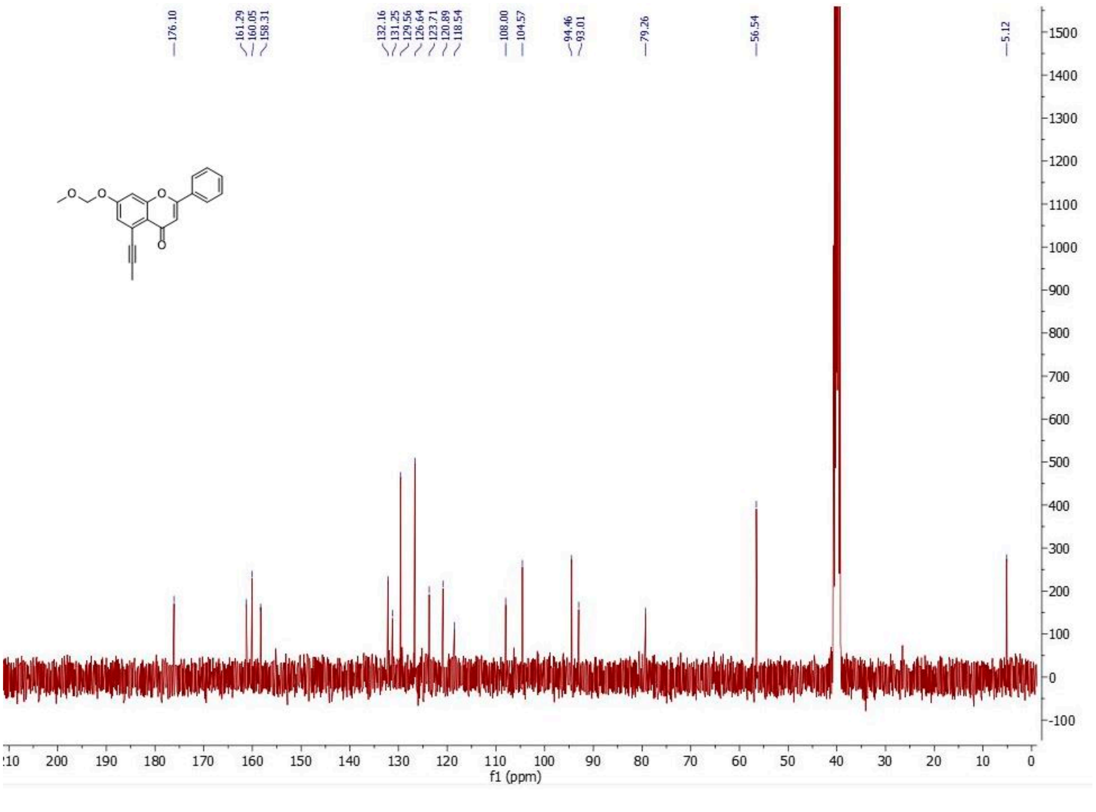

^{1}H NMR spectrum of compound 10e (400 MHz, DMSO-d_6)

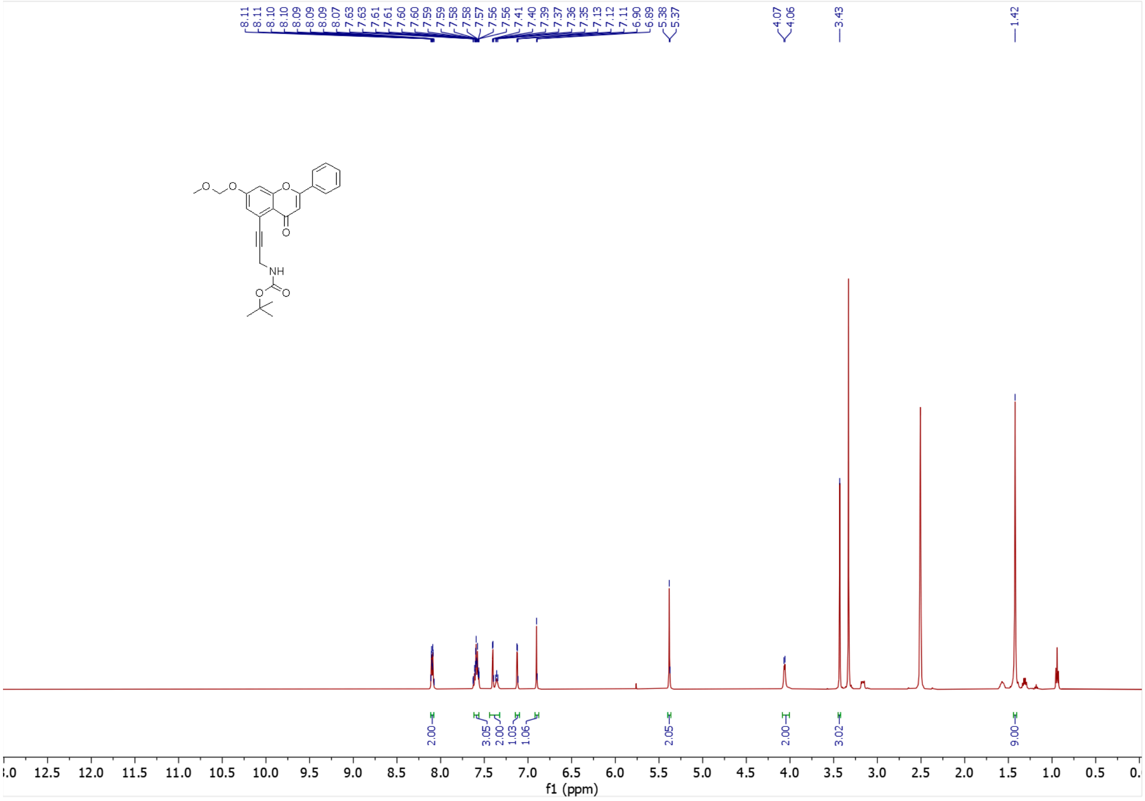

^{13}C NMR spectrum of compound 10e (101 MHz, DMSO-d_6)

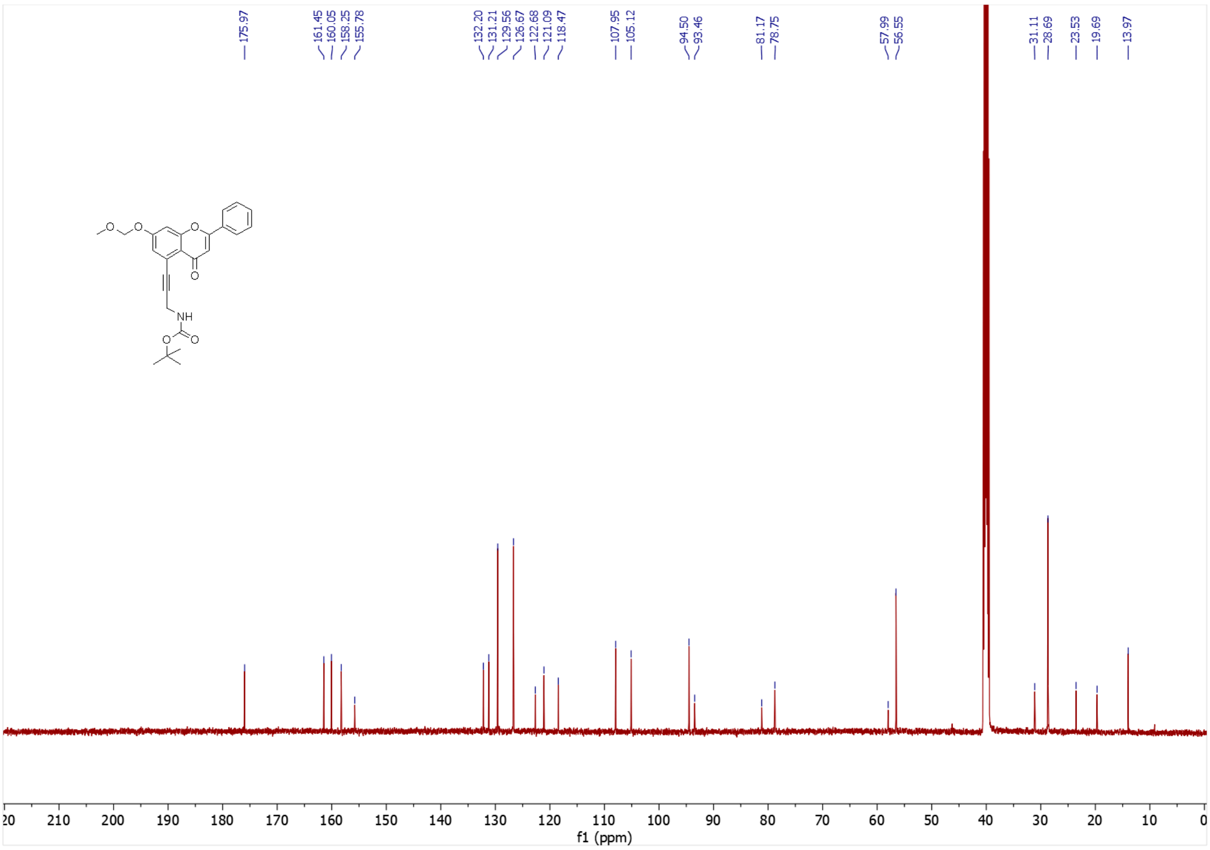

^{1}H NMR spectrum of compound 10f (400 MHz, DMSO-d_6)

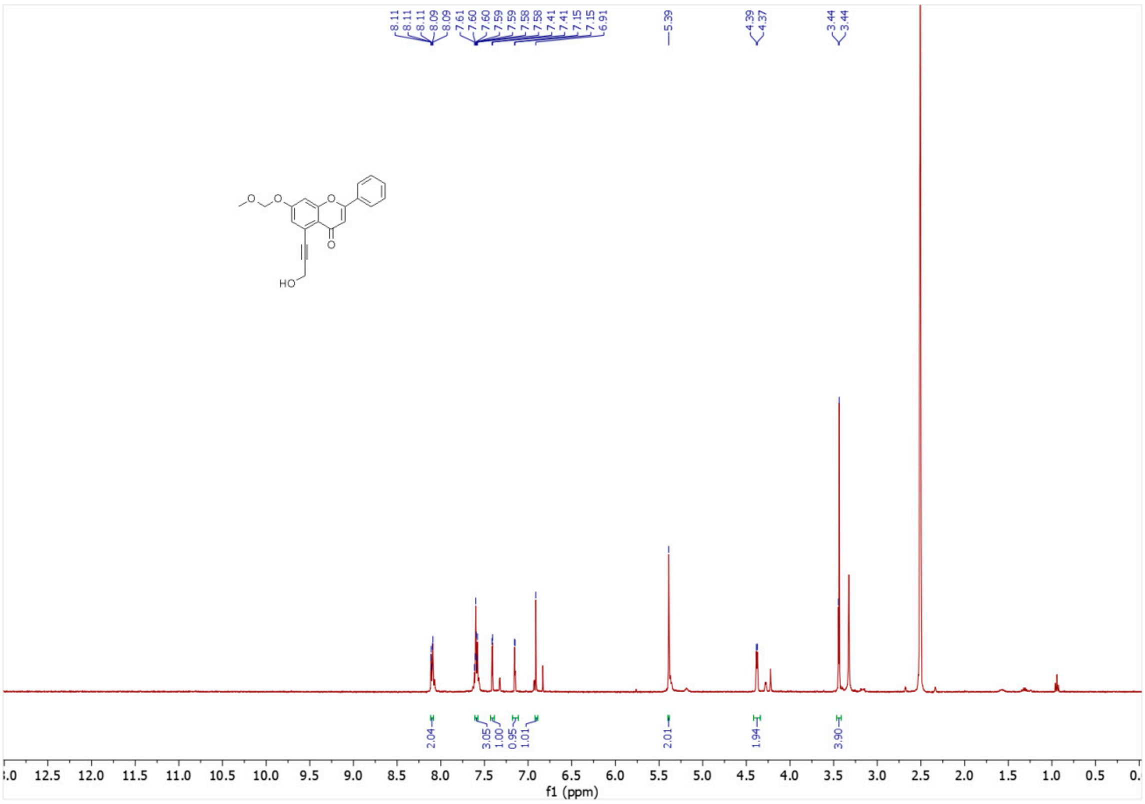

^{13}C NMR spectrum of compound 10f (101 MHz, DMSO-d_6)

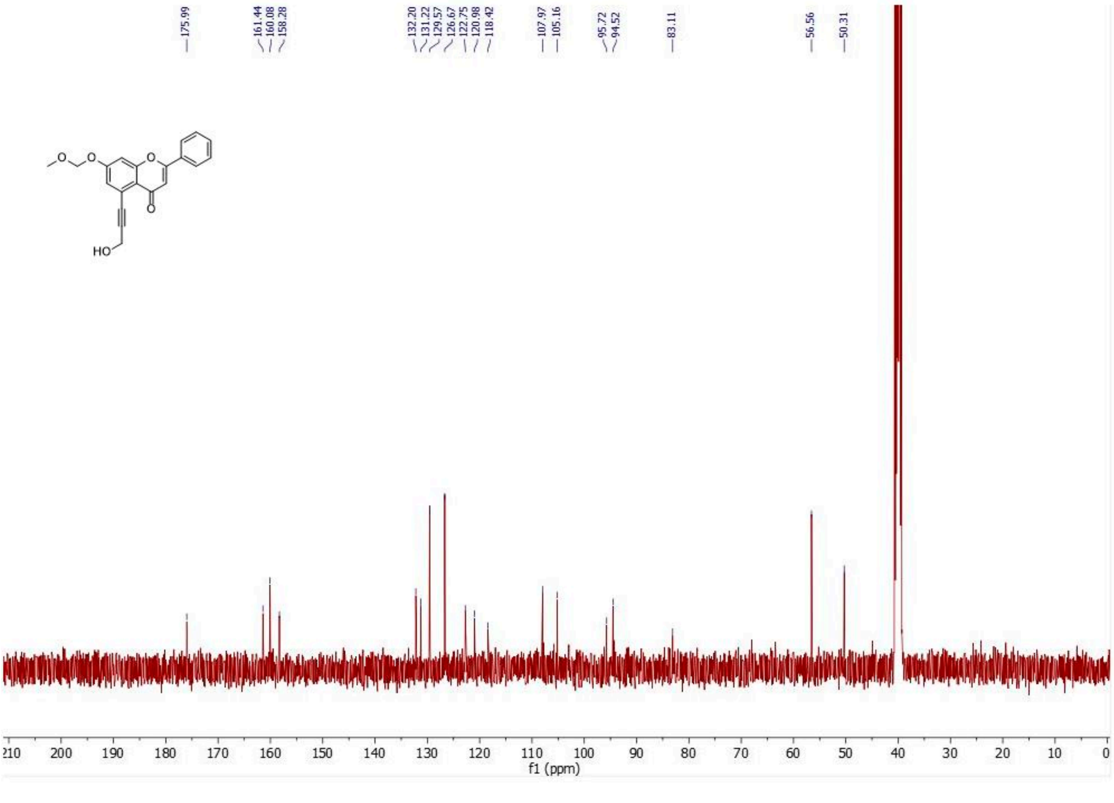

^{1}H NMR spectrum of compound 10h (400 MHz, CD_2Cl_2)

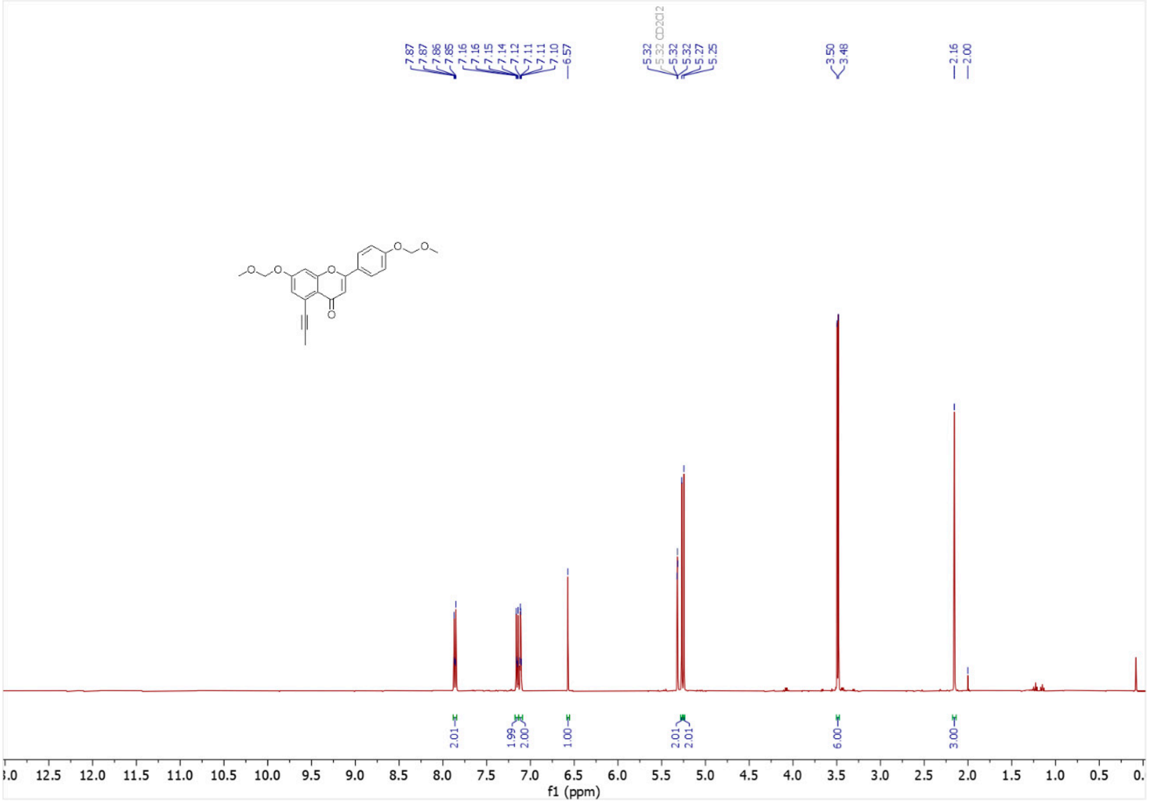

^{13}C NMR spectrum of compound 10h (101 MHz, CD_2Cl_2)

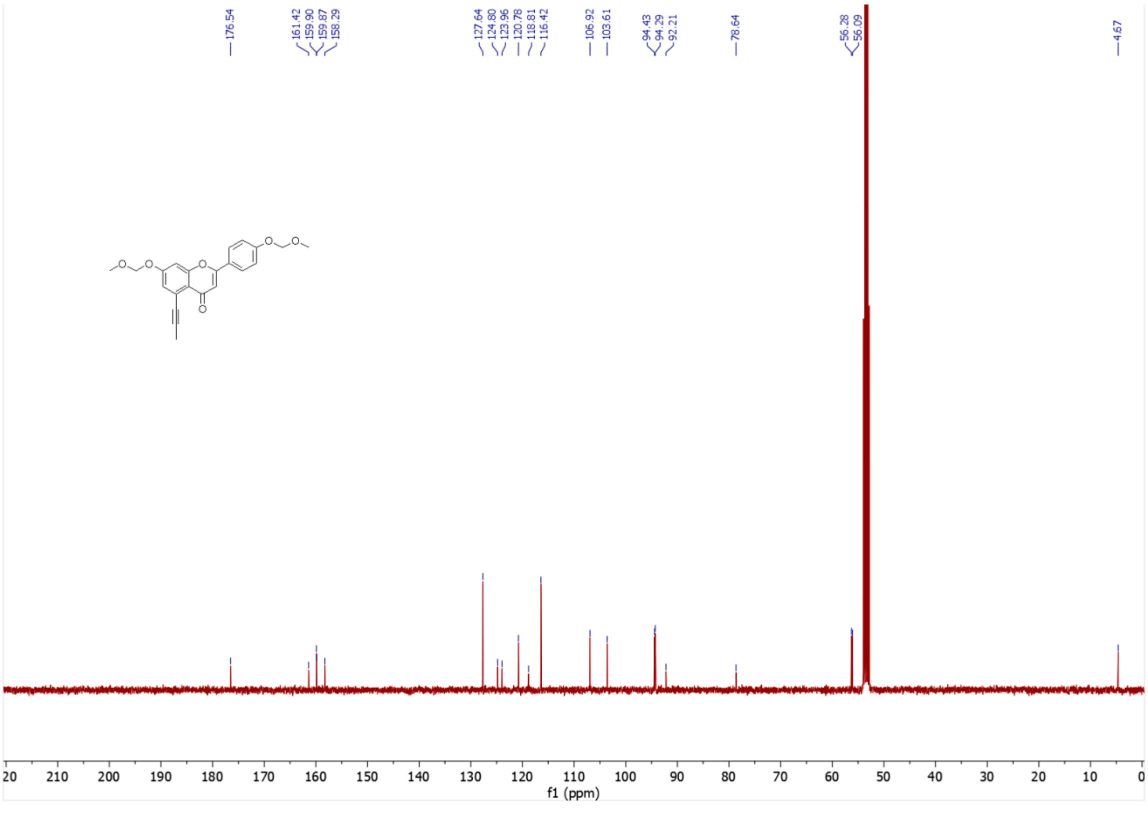

^{1}H NMR spectrum of compound 17b (400 MHz, CD_2Cl_2)

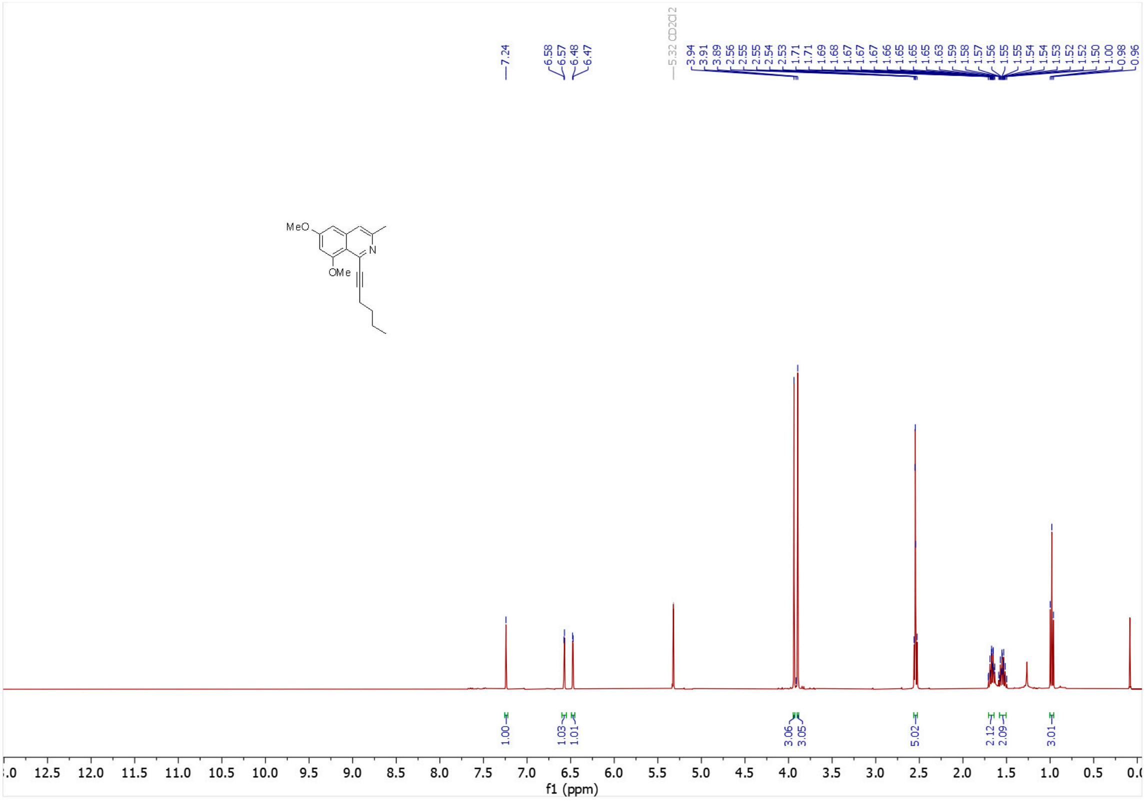

^{13}C NMR spectrum of compound 17b (101 MHz, CD_2Cl_2)

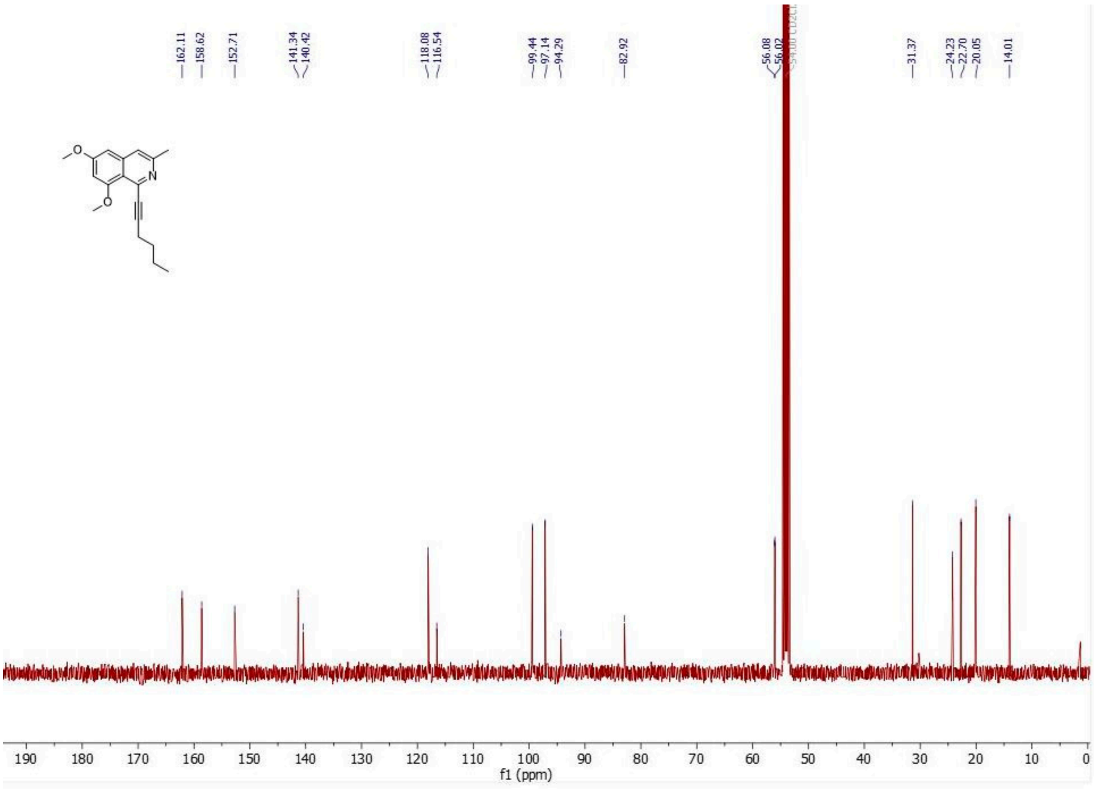

^{1}H NMR spectrum of compound 17c (400 MHz, CD_2Cl_2)

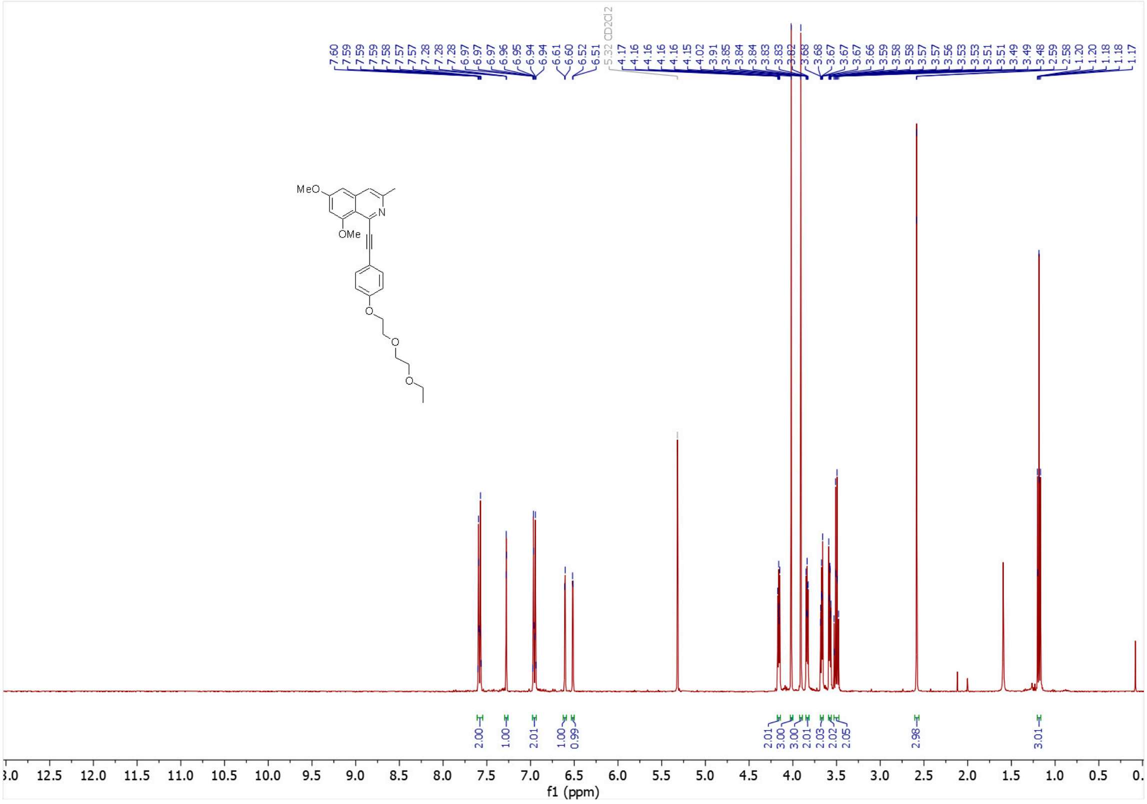

^{13}C NMR spectrum of compound 17c (101 MHz, CD_2Cl_2)

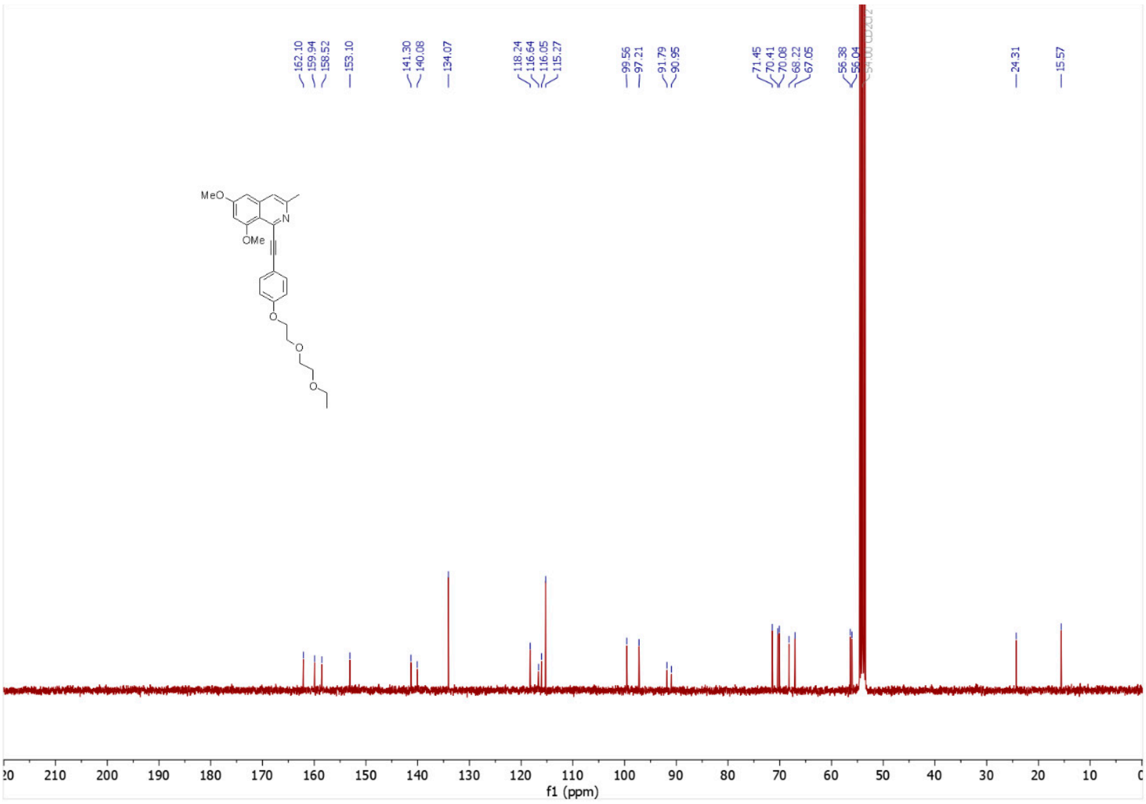

^{1}H NMR spectrum of compound 23 (400 MHz, DMSO-d_6)

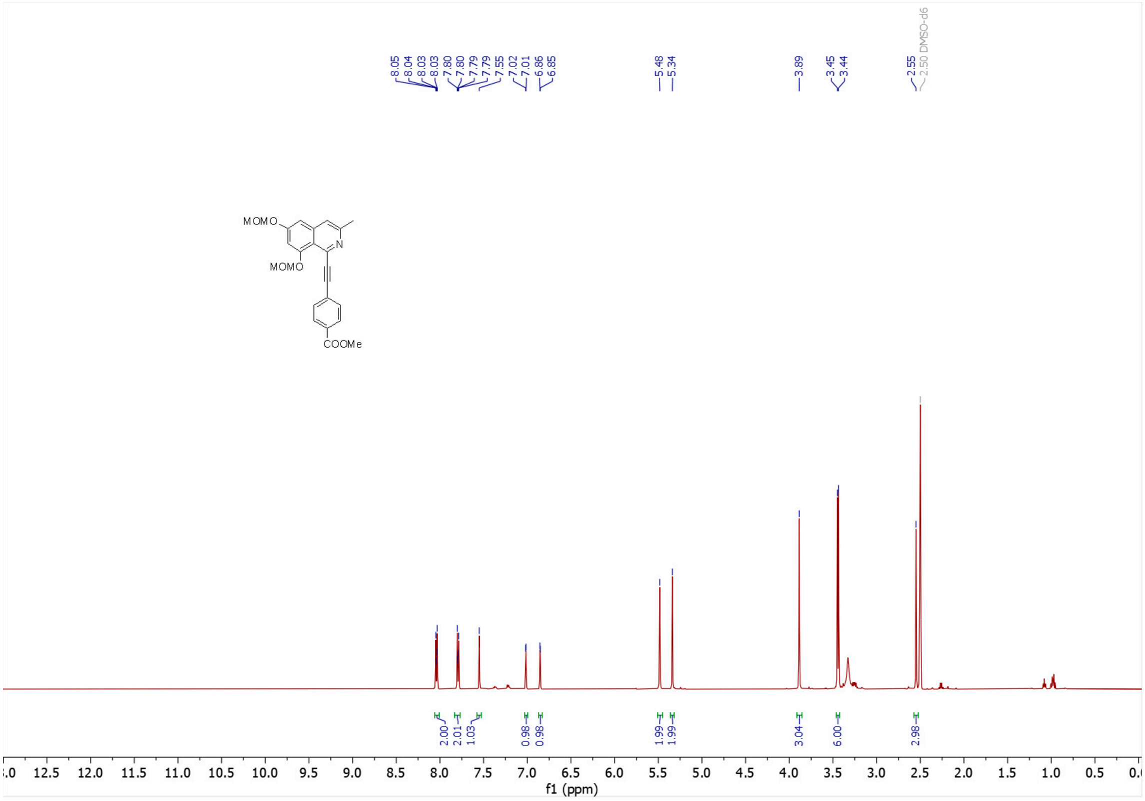

^{13}C NMR spectrum of compound 23 (101 MHz, DMSO-d_6)

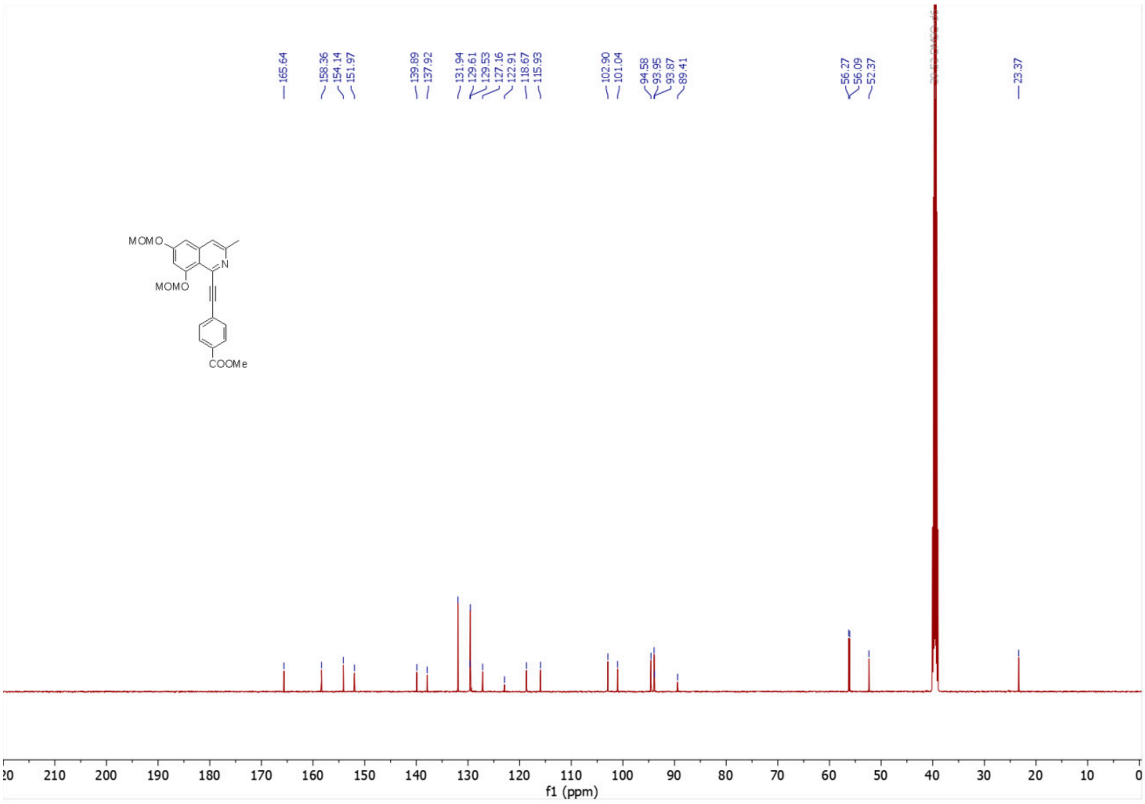

Supplement: Supplementary file 1 [file pharmaceuticals-18-01018-s001.zip › pharmaceuticals-3714233-supplementary.pdf]
